# Significance of LncRNA CASC8 genetic polymorphisms on the tuberculosis susceptibility in Chinese population

**DOI:** 10.1002/jcla.23234

**Published:** 2020-02-07

**Authors:** Guoye Liu, Rui Xia, Qian Wang, Zhiqiang Wang, Binwu Ying, Hong Yan

**Affiliations:** ^1^ Department of Laboratory medicine Affiliated Brain Hospital of Nanjing Medical University (Chest Branch) Nanjing China; ^2^ Department of Laboratory Medicine West China Hospital Sichuan University Chengdu China

**Keywords:** LncRNA CASC8, single nucleotide polymorphism (SNP), tuberculosis susceptibility

## Abstract

**Background:**

Tuberculosis remains an important disease threatening the security of public health, and no effective targets have been found for the immunological diagnosis or therapy of tuberculosis. The aim of this study was to explore the associations between lncRNA CASC8 genetic polymorphism and tuberculosis risk.

**Method:**

A total of 900 tuberculosis patients and 1534 healthy individuals in the Western Chinese Han population were recruited for our study. Candidate SNPs of CASC8 were initially filtered by importing the 1000 genomes database into Haploview, and subsequently genotyped using modified multiplex ligation detection reactions.

**Results:**

The lncRNA CASC8 genetic variant rs7836840 was associated with an increased tuberculosis risk with a *P*‐value of .034, but .134 after Bonferroni correction. Using subtype analysis, the C allele in rs7836840 showed a significant association with tuberculosis susceptibility (OR = 1.196, 95% CI = 1.05‐1.362, *P* = .02739 after Bonferroni correction). Patients carrying genotype AG and GG of rs7825118 and rs9297758 exhibited lower Hb concentrations (*P* = .006) and neutrophil counts (*P* = .015), respectively, while genotype AG and AA in rs6981424 demonstrated higher levels of ALT (*P* = .005) and AST (*P* = .033) in a dominant model, which were consistent with a tendency toward increased TB risk.

**Conclusions:**

This study was the first to explore the association between lncRNA CASC8 polymorphisms and TB infection risk and clinical manifestations. Our results provide evidence that CASC8 may act as a biomarker for the progression of clinical tuberculosis.

## 1 INTRODUCTION

Tuberculosis (TB) caused by *M tuberculosis (MTB)* is a chronic communicable disease that remains one of the top 10 causes of death in the world and is the leading cause of death by a single infectious agent. According to the World Health Organization (WHO), 10 million people are estimated to have developed TB in 2018, and an estimated 0.39 million had multidrug‐resistant TB (MDR‐TB). About 1.2 million people die from tuberculosis each year.^1^ Although an estimated 1.7 billion people are infected with *MTB* worldwide, only 5%‐10% of latent tuberculosis infection (LTBI) individuals will go on to develop active TB.^2^ This indicates that host genetic factors, mainly associated with “candidate” genes, including the genes encoding human leukocyte antigen (HLA), killer immunoglobulin‐like receptor (KIR), toll‐like receptors (TLRs), cytokine/chemokines and their receptors, vitamin D receptor (VDR) and SLC11A1 play important roles in determining individual susceptibility to infectious TB.^3^ However, the roles of genetic polymorphisms in the regulation of TB have rarely been described, and therefore, there is an urgent need to explore more genetic loci which affect the clinical phenotypes and might assist in the diagnosis of clinical TB.

Long non‐coding RNAs (lncRNAs) refer to types of ncRNA that are longer than 200 nucleotides and have no protein‐coding potential. Studies are increasingly focused on lncRNAs and their underlying roles in the occurrence and development of human diseases. It is well known that lncRNAs are involved in the regulation of mRNA translations, RNA maturation and transport, protein synthesis,^4^ cell differentiation, and tissue development. According to recent reports, lncRNAs have been shown to be involved in the dysregulation of cancer,^5^ cardiovascular diseases^6^ and especially infectious diseases.^7^ For instance, aberrantly expressed lncRNAs HEAL in T cells are involved in HIV infection.^8^ Additionally, many lncRNAs, like MEG3^9^ and PCED1B‐AS1^10^ in macrophages, lncRNAs CD244^11^ in CD8^+^T cells and some lncRNAs in CD4^+^T cells were differentially expressed in patients with TB. Furthermore, our research team previously reported the presence of differentially expressed lncRNA in peripheral blood mononuclear cells (PBMCs) in response to multidrug‐resistant TB (MDR‐TB) infection.^12^ These data reveal that lncRNAs play important roles in TB infection, and therefore, we selected to investigate lncRNA polymorphisms and their association with human susceptibility to clinical TB.

**Figure 1:**
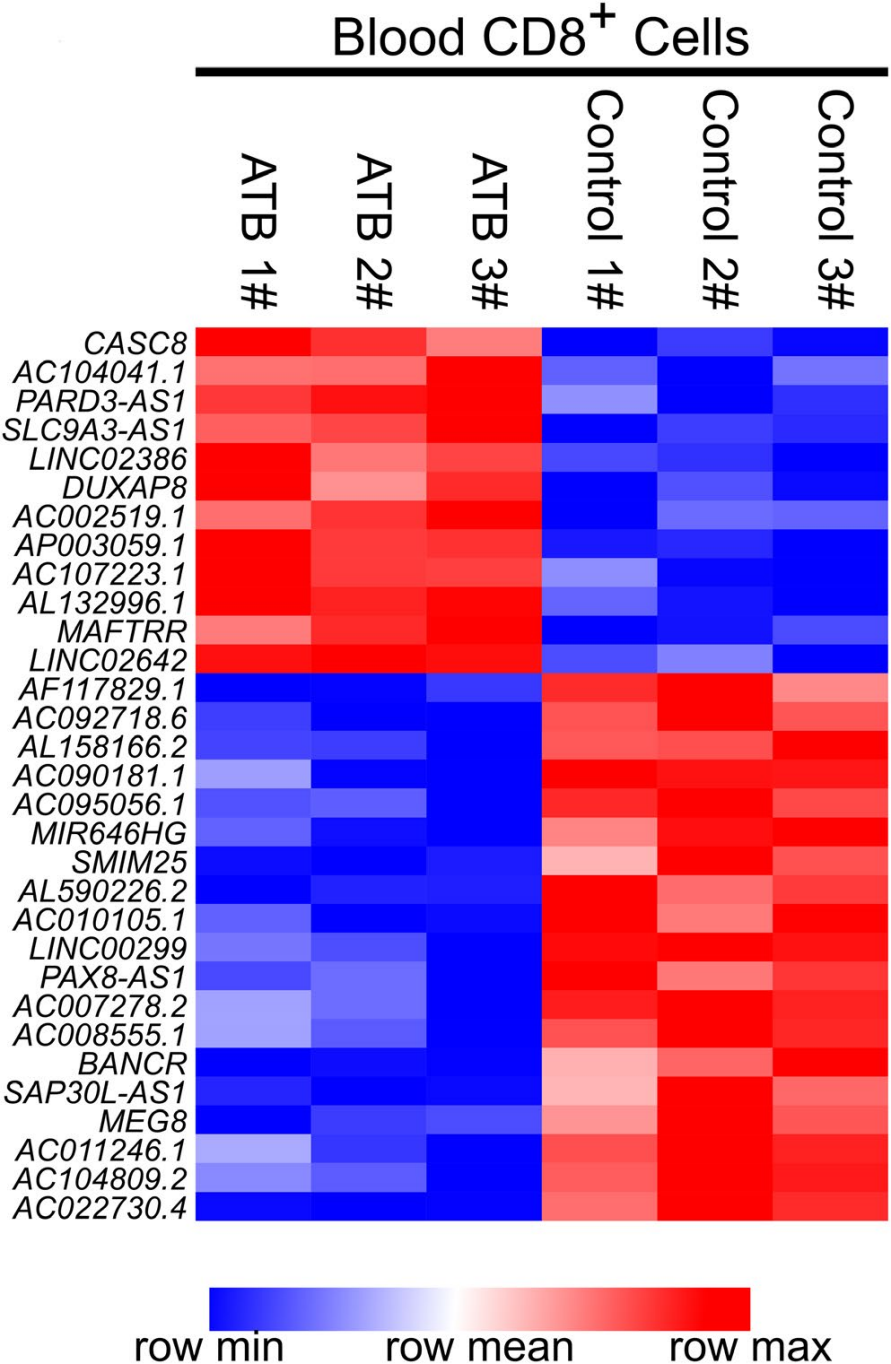
Differentially expressed LncRNA CASC8 between ATB and control group. High relative expression was indicated by red color, and low relative expression was indicated by blue color

In human TB, CD8+ T cells have been proven to contribute to host defenses by releasing Th1 cytokines or directly killing *Mtb*‐infected macrophages. Fu et al^13^ conducted microarray analysis to investigate differentially expressed lncRNA and mRNA genes in CD8+ T cells isolated from active TB group and healthy control group and recorded these profiles in the GEO database (GSE97530). For a more comprehensive lncRNA profile, we reannotated microarray probes with GENCODE Release 32 (GRCh38.p13) and re‐analyzed this microarray. Differentially expressed lncRNAs profile (Figure 1) showed that the lncRNA transcript CASC8 encoded by gene CASC8 (Ensembl ID ENSG00000246228) was upregulated in CD8+ T cells from active TB (ATB) when compared to healthy controls. It is possible that the lncRNA CASC8 gene may be involved in the establishment and progression of TB infection. CASC8 (also known as LINC00860), located at the 8q24.21, is a gene that is associated with an increased risk of cleft lip/palate^14^, ^15^ and several malignancies including colorectal cancer.^16^ Recent evidence has indicated that the development of colorectal cancer (CRC) requires a series of inflammatory and immunological factors to enable and shape a tumorigenic milieu.^17^ It is also reported that the expression of CASC8 is correlated with the SNPs inside the CASC8 gene body.^18^ For example, the rs10505477 variant in lncRNA CASC8 has a strong correlation with CRC susceptibility, the risk of lung cancer and the prognosis for gastric cancer, which suggests that upregulated CASC8 in CD8+ T cells may be explained partly by genetic polymorphisms. Considering this information, we hypothesized that the genetic polymorphisms of the CASC8 gene may be involved in TB susceptibility and may act as new biomarkers for clinicians in the diagnosis, therapy, and prognostic evaluation of TB.

China ranks second among 22 high TB burden countries, with an estimated 1.4 million Chinese patients suffering from TB every year.^1^ The high incidence of tuberculosis combined with limited tools for laboratory diagnosis of TB has led us to focus on studying new molecular biomarkers of TB. Thus, our study selected a set of SNPs within CASC8 among 900 tuberculosis cases and 1534 healthy controls in the Han Chinese population and systematically analyzed the associations between lncRNA polymorphisms and the occurrence and clinical characterization of TB infections.

## 2 METHODS AND MATERIALS

### 2.1 Study subjects

Our study enrolled 900 tuberculosis patients and 1534 healthy controls. All subjects were recruited from the West China Hospital of Sichuan University between January 2014 and February 2016. In the case study group, we recruited patients that had a primary diagnosis of TB that was confirmed by two independent experienced respiratory physicians. The criteria for all enrolled patients included: typical symptoms, recent radiographic evidence of TB cavitation, and positive laboratory data (smear/culture/TB‐DNA).

Patients with HIV infection, diseases associated with immunodeficiency, hepatitis virus infection, and other lung diseases were excluded from this study. The control subjects were selected from a pool of patients with no history of TB that had received a recent physical examination with normal laboratory and imaging data. All participants were unrelated Han Chinese. Demographic data and clinical information of the studied populations were reviewed from the medical record system of West China Hospital of Sichuan University. Two to 3 mL of EDTA‐anticoagulated whole blood samples were collected from all participants including healthy controls and newly admitted patients that meet enrolled criteria and stored in the Bio‐Bank of “Tuberculosis Research” in the Department of Laboratory Medicine, West China Hospital, Sichuan University, China. The ethics of this research project were approved by the Clinical Trial and Biomedical Ethics Committee of West China Hospital of Sichuan University, and written informed consent was obtained from all participants.

### 2.2 Single nucleotide polymorphisms (SNPs) selection and genotyping

Genetic variants of CASC8 were obtained from the dbSNP database (http://www.ncbi.nlm.nih.gov/projects/SNP/). Candidate SNPs were filtered as dbSNP of CASC8 from the 1000 genomes database into Haploview, and tagSNPs were selected if they were represented with a minor allele frequency (MAF) ≥ 0.05 in the Han Chinese population of Beijing (CHB). Then, the tagSNPs located in potentially functional regions like promoters, exons, untranslated regions (UTRs), and introns were preferentially selected. We selected 4 SNPs of the CASC8 gene including rs7825118, rs6981424, rs9297758, and rs7836840 for subsequent genotyping.

Our study used the QIAamp^®^ DNA Blood Mini Kit (Qiagen) to isolate genomic DNA from peripheral blood samples and candidate SNPs were genotyped using a modified multiplex ligation assay (Genesky Biotechnologies Inc). A 0.5 μL ligation product was fractionated and loaded in ABI 3730XL, and GeneMapper v4.1 software was employed to analyze the raw data. Fluorescent labels of allele‐specific oligonucleotide probe pairs distinguished specific alleles of each SNP, and the differentiated extension length of the different SNPs at the 3′ end site. Genotyping was performed as a blinded experiment so that the experimenter was unaware of the status of the case controls. Moreover, we introduced ddH_2_O as a negative control in the experiment to monitor genotyping quality. Furthermore, we randomly selected approximately 10% for genotyping, with a 100% concordance rate.

### 2.3 Statistical analysis

In our experiment, the chi‐square test and the Mann‐Whitney *U* test were used to analyze categorical variables and continuous variables, respectively. The goodness‐of‐fit chi‐squared test was applied to assess the Hardy‐Weinberg equilibrium (HWE) for all SNPs in all samples. The statistical method was performed using SPSS version 19.0 (IBM Corp.). The associations between candidate SNPs and TB infections was determined based on the distribution of allele and genotype frequencies as well as genetic models (dominant and recessive models). Furthermore, an unconditional logistic regression analysis by PLINK v1.07 was used to estimate odds ratios (ORs) and 95% confidence intervals (CIs) while correcting for age and gender. Calibration of the tests was carried out using the Bonferroni method. Fernando found that SNPs are preferentially associated with TB sub‐phenotypes. Correlation analysis between candidate SNPs and TB was then applied to a stratified analysis including tuberculosis (PTB), extra‐pulmonary tuberculosis (EPTB), and tuberculosis with extra‐pulmonary tuberculosis (PTB & EPTB) that were compared to healthy controls. In addition, we estimated the linkage disequilibrium (LD) by establishing the *r* ^2^ coefficient of the SNP loci with Haploview v4.2 and performed haplotype analysis among TB case‐control studies based on the expected maximization‐clustering algorithm. Furthermore, all TB subjects' samples were used for evaluating the correlation between genotypes and clinical phenotypes. The closely related clinical laboratory data (ALB and GLB related to impaired liver function in tuberculosis; WBC, Hb, ESR, and CRP involved in inflammatory responses in tuberculosis), apart from some missing data, were collected for this analysis. The chi‐square test was used, all statistical tests were 2‐sided, and *P* < .05 was considered statistically significant.

## 3 RESULTS

### 3.1 Demographics and clinical characterization of subjects

Table 1 demonstrates that there were statistically significance differences between TB patients and healthy controls for both age and gender (*P* < .001). Among the 900 TB patients enrolled, 657 PTB, 93 EPTB, and 150 PTB & EPTB were included.

**Table 1:** Demographic characteristics of participants in Chinese Han population

Features	TB (n = 900)	HC (n = 1534)	*P*
General information			
Age, mean ± SD (y)	42.51 ± 18.11	37.96 ± 11.07	**<.001**
Male (%)	542 (60.22%)	821 (53.52%)	**<.001**
TB clinical subtype n (%)			
PTB	657 (73.0)		
EPTB	93 (10.3)		
PTB & EPTB	150 (16.7)		

Abbreviations: EPTB, extra‐pulmonary tuberculosis; HC, healthy controls; PTB & EPTB, pulmonary tuberculosis combined with extra‐pulmonary tuberculosis; PTB, pulmonary tuberculosis; TB, tuberculosis.

Bold indicates statistical significant value.

### 3.2 Associations between LncRNA polymorphisms and TB susceptibility

#### 3.2.1 Correlation analysis of single SNPs

In the case‐control population, 4 SNPs (Table S1) were successfully genotyped and the controls were consistent using the Hardy‐Weinberg equilibrium (HWE) rule (*P* < .05). The chromosomal location, MAF and HWE *P* values information for all SNPs are shown in Tables S2 and S3.

The genotype distribution and allele frequency data of 4 selected SNPs, among all TB cases and controls, associated with CASC8 gene are presented in Table 2. Rs7836840 with the mutant allele C (A>C) showed significant differences in both genotype distribution (*P* = .048) and allele frequency (*P* = .034, OR = 1.135, 95% CI = 1.01‐1.28) between TB patients and the control group after adjusting for age and gender. However, after adjustments were made by Bonferroni correction, the underlying association disappeared (*P* = .191 and 0.134). Furthermore, genetic model analyses (dominant model and recessive model) were carried out to further explore the differences in genotype distributions. Table 3 demonstrates that the dominant model of rs7836840 is associated with increasing TB risk and the estimated OR of 1.236, ranging from 1.029 to 1.483 (*P* value of .023 after adjusting for age and gender). The *P* value was .093 with no significant association after adjustment with Bonferroni correction, three tests this correction is done. Another three SNPs demonstrated no significant differences between TB cases and controls.

**Table 2:** Genetic polymorphisms of CASC8 in patients with TB

SNP	Variant	Case, n (%)	Control, n (%)	OR (95%CI)	*P*	P**	Variant	Case, n (%)	Control, n (%)	P*	P**
rs7825118	A	413 (22.94)	739 (24.09)	0.9366 (0.82‐1.08)	.3508	–	AA	47 (5.22)	89 (5.8)	.2446	.9266
G>A	G	1379 (76.61)	2311 (75.33)				AG	319 (35.44)	561 (36.57)		
							GG	530 (58.89)	875 (57.04)		
rs6981424	A	418 (23.22)	666 (21.71)	1.087 (0.95‐1.25)	.2399	.9595	AA	48 (5.33)	69 (4.5)	.2316	.9785
G>A	G	1380 (76.67)	2390 (77.9)				AG	322 (35.78)	528 (34.42)		
							GG	529 (58.78)	931 (60.69)		
rs9297758	A	680 (37.78)	1193 (38.89)	0.957 (0.85‐1.08)	.4724	–	AA	130 (14.44)	223 (14.54)	.3638	–
G>A	G	1112 (61.78)	1867 (60.85)				AG	420 (46.67)	747 (48.7)		
							GG	346 (38.44)	560 (36.51)		
rs7836840	C	825 (45.83)	1309 (42.67)	1.135 (1.01‐1.28)	**.03352**	.1341	CC	178 (19.78)	279 (18.19)	**.04773**	.1909
A>C	A	973 (54.06)	1753 (57.14)				CA	469 (52.11)	751 (48.96)		
							AA	252 (28)	501 (32.66)		

Abbreviations: OR, odds ratio; P**, *P* value after Bonferroni correction; P*, *P* value after adjusting for gender and age; SNP, single nucleotide polymorphism.

Bold indicates statistical significant value.

**Table 3:** Analysis of CASC8 gene polymorphisms relevant to TB risk in Chinese Han population

SNP	Dominant model			Recessive model		
	OR (95% CI)	P*	P**	OR (95% CI)	P*	P**
rs7825118 G>A	0.901 (0.76‐1.068)	.2305	.9218	0.9159 (0.6338‐1.324)	.6401	
rs6981424 G>A	1.095 (0.9237‐1.298)	.2957	–	1.189 (0.8095‐1.746)	.3776	
rs9297758 G>A	0.9089 (0.7649‐1.08)	.2777	–	0.9677 (0.7631‐1.227)	.7867	
rs7836840 A>C	1.236 (1.029‐1.483)	**.02336**	.09342	1.095 (0.8853‐1.354)	.4033	

Bold indicates statistical significant value.

Using a methodology that was similar to that described by Fernando et al, we further examined whether these four candidate SNPs are preferentially associated with a specific TB subtype. Results are presented in Tables 4 and 5. After Bonferroni correction, rs7836840 with a minor C allele had a significantly increased risk of PTB in both genotype distribution (*P* = .050) and allele frequency (OR = 1.196, 95% CI = 1.05‐1.362, *P* = .027) when compared to all forms of TB. The genotype distribution of rs7825118 was significantly associated with PTB patients before Bonferroni correction when compared with all forms of TB (*P* = .046 after adjusting for age and gender; *P* = .1834 after Bonferroni correction). In addition, rs7836840 showed significantly increasing association with PTB susceptibility (OR = 1.327, 95% CI = 1.078‐1.633, *P* = .03, after Bonferroni correction) in the dominant model. No significant differences were identified among the other three SNPs in the analysis of the PTB subgroup.

**Table 4:** Genetic polymorphisms of CASC8 in patients with PTB

SNP	Variant	Case, n (%)	Control, n (%)	OR (95%CI)	*P*	P**	Variant	Case, n (%)	Control, n (%)	P*	P**
rs7825118	A	285 (21.69)	739 (24.09)	0.8729 (0.7474‐1.02)	.08605	.3442	AA	28 (4.26)	89 (5.8)	**.04584**	.1834
G>A	G	1021 (77.7)	2311 (75.33)				AG	229 (34.86)	561 (36.57)		
							GG	396 (60.27)	875 (57.04)		
rs6981424	A	301 (22.91)	666 (21.71)	1.068 (0.9153‐1.247)	.4018	–	AA	32 (4.87)	69 (4.5)	.4305	–
G>A	G	1011 (76.94)	2390 (77.9)				AG	237 (36.07)	528 (34.42)		
							GG	387 (58.9)	931 (60.69)		
rs9297758	A	475 (36.15)	1193 (38.89)	0.8945 (0.7823‐1.023)	.1033	.4132	AA	86 (13.09)	223 (14.54)	.06532	.2613
G>A	G	831 (63.24)	1867 (60.85)				AG	303 (46.12)	747 (48.7)		
							GG	264 (40.18)	560 (36.51)		
rs7836840	C	619 (47.11)	1309 (42.67)	1.196 (1.05‐1.362)	**.00685**	**.02739**	CC	138 (21)	279 (18.19)	**.01253**	.05013
A>C	A	693 (52.74)	1753 (57.14)				CA	343 (52.21)	751 (48.96)		
							AA	175 (26.64)	501 (32.66)		

Abbreviations: OR, odds ratio; P**, *P* value after Bonferroni correction; P*, *P* value after adjusting for gender and age; SNP, single nucleotide polymorphism.

Bold indicates statistical significant value.

**Table 5:** Analysis of CASC8 gene polymorphisms relevant to PTB risk in Chinese Han population

SNP	Dominant model			Recessive model		
	OR (95% CI)	P*	P**	OR (95% CI)	P*	P**
rs7825118 G>A	0.8344 (0.6886‐1.011)	.06477	.2591	0.755 (0.4847‐1.176)	.2138	–
rs6981424 G>A	1.089 (0.9002‐1.318)	.3795	–	1.032 (0.6636‐1.606)	.888	–
rs9297758 G>A	0.8417 (0.6945‐1.02)	.07908	.3163	0.8503 (0.6463‐1.119)	.2464	–
rs7836840 A>C	1.327 (1.078‐1.633)	**.00759**	**.03035**	1.167 (0.9231‐1.474)	.1972	–

Bold indicates statistical significant value.

#### 3.2.2 Linkage analysis and haplotype construction

**Figure 2:**
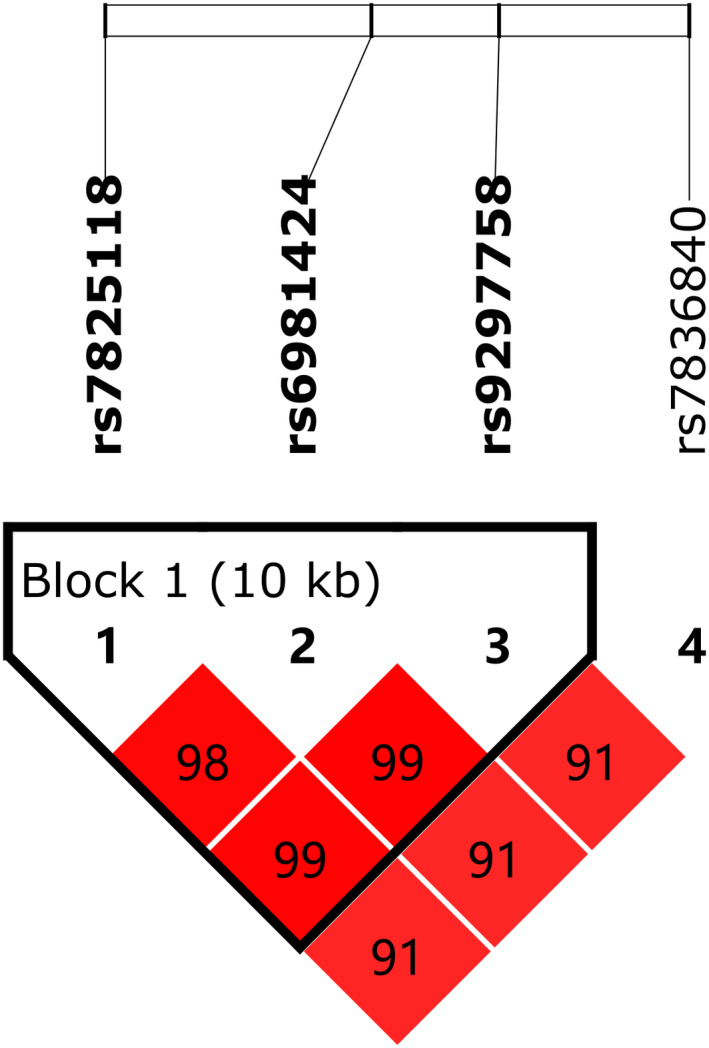
Linkage disequilibrium (LD) plot of 4 SNPs of LincRNA CASC8. Strong LD is represented by a higher percentage and a darker square

Figure 2 shows the linkage disequilibrium (LD) plot of SNPs in the CASC8 gene. With a threshold of pairwise *r* ^2^ > .8, three genetic variants of the CASC8 gene including rs7825118, rs6981424, rs9297758 were in one linkage disequilibrium (LD) block. Four haplotypes including GGG, AGA, GAG, and GGA among the CASC8 gene were constructed using the strong LD state from Figure 2, and no significant association of these haplotype frequencies were observed between the TB cases and the controls (Table 6).

**Table 6:** Haplotypes analysis of CASC8 gene associated with the risk of TB

Haplotype	Frequency			OR (95% CI)	*P* value
	All	Case	Control		
GGG	0.391	0.389	0.393	0.989 (0.877‐1.114)	.8505
AGA	0.237	0.231	0.241	0.942 (0.821‐1.081)	.3946
GAG	0.223	0.232	0.218	1.088 (0.946‐1.251)	.2363
GGA	0.148	0.148	0.149	0.991 (0.841‐1.168)	.9146

#### 3.2.3 Correlation between genotypes and clinical phenotypes

**Figure 3:**
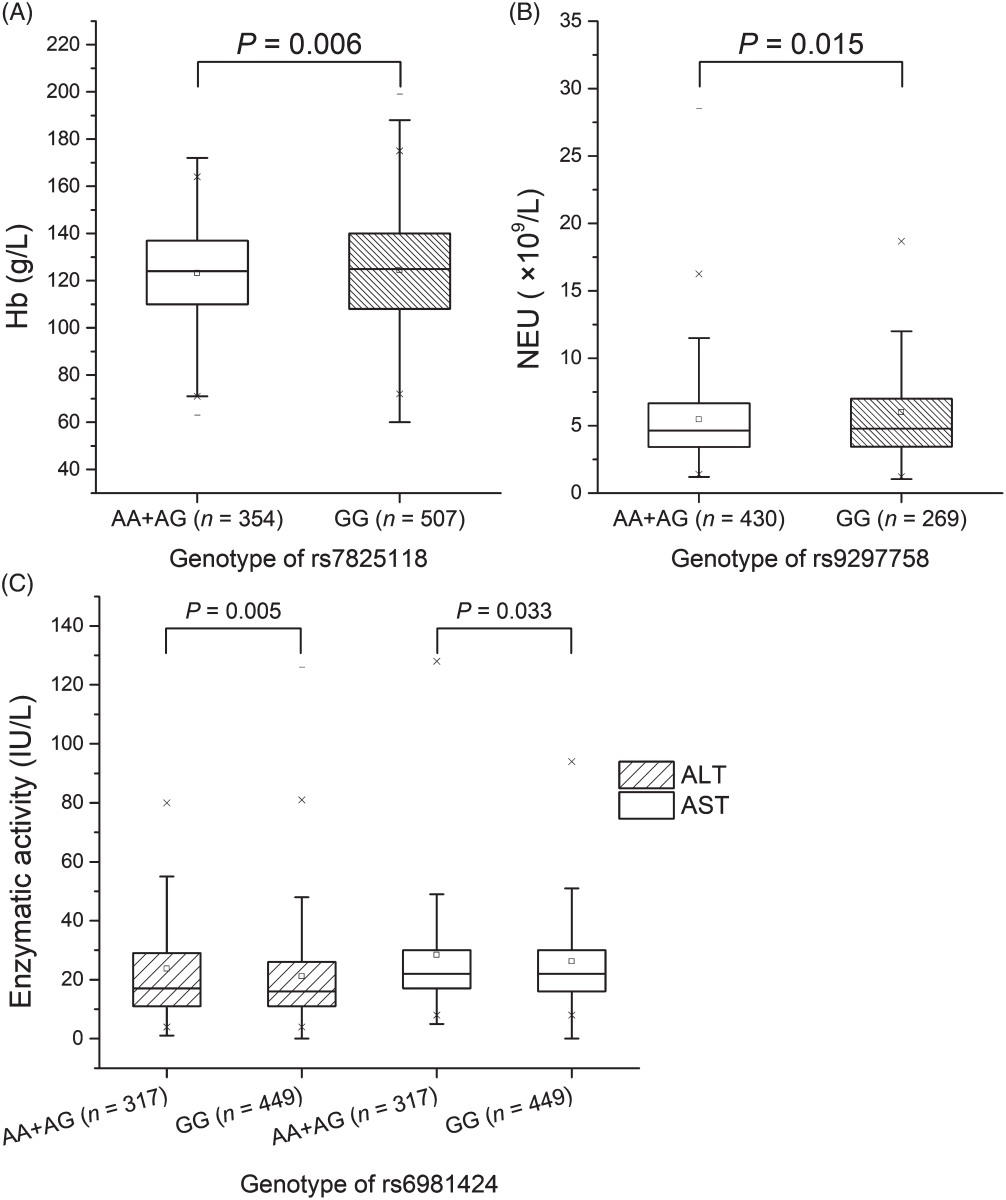
The level of clinical indicators in relation to polymorphisms of CASC8 in TB patients. A refers to serum Hb levels in relation to rs7825118 (G>A), B refers to serum Neu accounts in relation to rs9297758 (G>A), C refers to hepatic enzyme activity in relation to rs6981424 (G>A). Genotype was stratified on the basis of the dominant model, AA+AG vs GG

We further explored whether the four candidate SNPs influence the clinical features of TB patients. Common indices of TB severity include clinical symptoms and laboratory indicators such as albumin (ALB), globulin (GLB) and hemoglobin (Hb) concentrations, white blood cell count (WBC), erythrocyte sedimentation rate (ESR), and C‐reactive protein (CRP) levels. Because some homozygous minor genotypes occur infrequently, we applied dominant and recessive models to stratify the 4 SNPs. Although rs7825118 and rs9297758 were not shown to be significantly associated with TB susceptibility in the above SNP analysis, they were closely associated with Hb and neutrophil (Neu) levels (*P* = .006 and 0.015, respectively). Lower levels of Hb and Neu were displayed among patients possessing the AG+AA genotypes. Additionally, significant associations were discovered between rs6981424 and indices of liver function in patients, and more patients with the AA+AG genotype exhibited higher ALT and AST activities (*P* = .005 and .033 respectably) (Figure 3).

## 4 DISCUSSION

With the high prevalence and rapid dissemination of MDR‐TB in recent years, the regional disease burden of TB has become high, threatening health of the public in China.^19^ Our study is the first to identify the association between the 4 SNPs of LncRNA CASC8 and TB susceptibility. We demonstrated that tagSNP rs7836840 may be involved in the risk of the development of TB, particularly in the PTB subgroups. Further research on candidate SNP rs6981424 may determine the influence of Hb levels in TB patients. To the best of our knowledge, this is the first study to comprehensively analyze the strong associations between CASC8 and TB risk, and CASC8 may become a promising biomarker in the diagnosis of TB.

Long non‐coding RNAs are newly discovered transcripts that are more than 200 nt in length but without the capacity of protein encoding,^20^ and they are involved in regulating gene expression through a variety of molecular mechanisms. LncRNAs are highly tissue‐specific when compared to mRNA^21^, ^22^ and have the potential to be new targets for monitoring disease progression. However, there are currently insufficient data to demonstrate the impact of lncRNA genetic polymorphisms on TB susceptibility and clinical presentation. With this in mind, we conducted a case‐control study in the western Han Chinese population for correlation analysis.

CASC8 is located in the 8q24 region with few functional annotation genes, meaning that it is possible to carry multiple non‐coding transcripts to perform a variety of functions.^23^ Hu et al reported that lncRNA CASC8 suppresses the proliferation of bladder cancer cells by down‐regulating glycolysis.^24^ Moreover, the genetic polymorphism of CASC8 has also been reported as a biomarker for the diagnosis of lung, gastric, and colorectal cancer.^14^, ^25^, ^26^ However, there is no study investigating the relationship between the CASC8 polymorphism and TB susceptibility. Our study demonstrated that there is no significant association between TB risk (containing all subgroups) and all four SNPs were observed in CASC8 among the case‐control group in the Han population of western China. Subsequent subgroup analysis further confirmed the work of Fernando et al, describing that SNPs are preferentially associated with specific tuberculosis subtypes. This may indicate that the rs7836840 minor C allele is a potential risk factor for the development of PTB due to the statistically significant differences in allele frequency and genotype distribution between the additive and dominant models in rs7836840. However, no differences were observed between EPTB and PTB&EPTB groups. Differences in analyses between TB subgroups may reflect linkage disequilibrium with other undetermined susceptible loci in different populations or because sample sizes are limited and the EPTB and EPTB & PTB subgroups are low in power.

We investigated whether the dominant model of four candidate SNPs in CASC8 are related to the clinical manifestations of tuberculosis in TB patients with complete clinical data and the results of single SNP analysis. Notably, we found that the AA+AG genotype in 3 SNPs with a TB non‐susceptibility locus was related to lower Hb level in rs7825118 (*P* = .006), lower Neu counts in rs9297758 (*P* = .015), and higher ALT and AST activity in rs6981424 (*P* = .005 and *P* = .033). This indicates that patients carrying the susceptible genotype AA+AG may have a higher inflammatory risk and host defense response to TB infection. Previous studies have demonstrated that rs1055229 is significantly associated with ESR among TB patients in AC079767.4 without TB susceptibility.^27^ The mechanism that causes this difference is still unclear, and several hypotheses have been proposed. First, the genetic polymorphism of the lncRNA variants may affect different signaling pathways and play de‐regulatory roles in different stages of TB progression. Additionally, insufficient sample size, clustering differences and heterogeneity in the enrolled population may explain the lack of significant correlations between SNPs and TB risk.

Using the bioinformatics software HaploReg v4.1 (http://www.broadinstitute.org/), we found that the four polymorphic loci (rs7825118, rs6981424, rs9297758, and rs7836840) were located in the intron region of CASC8 and there were no genes encoding proteins nearby. Recently, many studies have focused on the function of introns, which are non‐coding DNA fragments of genes that are involved in gene splicing and play an important role in regulating cell growth under stressful conditions.^28^ In our disease association study, the rs7836840 genetic variant in the intron region was determined to be strongly associated with TB risk. This may affect the functional expression of genes through mechanisms such as transcription levels within intron region mutations, intron splicing enhancers, or silencers.^29^ Our findings provide useful information for the future investigation of the potential biological functions of SNP rs7836840 in the development of TB.

Gene levels and transcription levels are closely related to the development of disease. Our study is based on the correlation analysis between the expression of SNP by lncRNA and TB risk. In the next phase of our work, we will analyze the structural variation of genes and changes in downstream pathway regulation from the genetic level to explore the occurrence and development of TB.

Our study describes the first data describing the associations between CASC8 polymorphisms and TB in the western Han Chinese population. However, our study has some limitations: First, the gene‐environment or gene‐gene interaction was not further analyzed in SNPs studies without significant association with tuberculosis. Second, the sample size of our study is limited. Third, the heterogeneity of the population was limited as the samples collected were mainly in western China, so some polymorphisms with significant effects may have been undetected. Lastly, several samples in this study lacked sufficient clinical characterization due to poor patient compliance; ideally, our results should be confirmed in larger population‐based studies with more clinical detail.

In conclusion, this study investigated whether the SNP rs7836840 C allele of lncRNA CASC8 may act as potential risk factor associated with TB susceptibility. The AG+GG genotype in other 3 SNPs was correlated with serum Hb and Neu levels and hepatic enzyme activity in a dominant model. Our findings suggest that genetic polymorphisms of LncRNA CASC8 may play an important role in TB risk and inflammatory response and are expected to serve as a new target for diagnosing tuberculosis.
