# Supplementary material for: Significance of LncRNA CASC8 genetic polymorphisms on the tuberculosis susceptibility in Chinese population
Source: J Clin Lab Anal. 2020 Feb 7;34(6):e23234. doi: 10.1002/jcla.23234 (PMC7307370; doi:10.1002/jcla.23234)
Supplement: Supplementary file 1 [file JCLA-34-e23234-s001.docx]

**Table S1 Detail the information of 4 SNPs in CASC8**

| original | Chr | Start | End | Ref | Alt | Func.refGene | Gene.refGene | ObsHET | PredHET | HWpval | %Geno | FamTrio | MendErr | MAF |
| --- | --- | --- | --- | --- | --- | --- | --- | --- | --- | --- | --- | --- | --- | --- |
| rs7825118 | 8 | 128475817 | 128475817 | A | G | ncRNA_intronic | CASC8 | 0.311 | 0.336 | 0.585 | 100 | 0 | 0 | 0.214 |
| rs9297758 | 8 | 128486588 | 128486588 | A | G | ncRNA_intronic | CASC8 | 0.447 | 0.455 | 0.9836 | 100 | 0 | 0 | 0.35 |
| rs6981424 | 8 | 128483096 | 128483096 | A | G | ncRNA_intronic | CASC8 | 0.398 | 0.373 | 0.7179 | 100 | 0 | 0 | 0.248 |
| rs7836840 | 8 | 128491792 | 128491792 | C | A | ncRNA_intronic | CASC8 | 0.515 | 0.497 | 0.9116 | 100 | 0 | 0 | 0.461 |

ObsHET is the marker's observed heterozygosity.

PredHET is the marker's predicted heterozygosity (i.e. 2*MAF*(1-MAF)).

HWpval is the Hardy-Weinberg equilibrium p value, which is the probability that its deviation from H-W equilibrium could be explained by chance.

%Geno is the percentage of non-missing genotypes for this marker.

FamTrio is the number of fully genotyped family trios for this marker (0 for datasets with unrelated individuals).

MendErr is the number of observed Mendelian inheritance errors (0 for datasets with unrelated individuals).

MAF is the minor allele frequency (using founders only) for this marker.
