# Supplementary material for: Significance of LncRNA CASC8 genetic polymorphisms on the tuberculosis susceptibility in Chinese population
Source: J Clin Lab Anal. 2020 Feb 7;34(6):e23234. doi: 10.1002/jcla.23234 (PMC7307370; doi:10.1002/jcla.23234)
Supplement: Supplementary file 2 [file JCLA-34-e23234-s002.docx]

Supplemental data

Table S2 MAF p values information for all CASC8 SNPs

| CHR | SNP | A1 | A2 | MAF | NCHROBS |
| --- | --- | --- | --- | --- | --- |
| 8 | rs7825118 | A | G | 0.2379 | 4842 |
| 8 | rs6981424 | A | G | 0.2233 | 4854 |
| 8 | rs9297758 | A | G | 0.386 | 4852 |
| 8 | rs7836840 | C | A | 0.4391 | 4860 |

Table S3 HWE p values information for all CASC8 SNPs

| CHR | SNP | TEST | A1 | A2 | GENO | O(HET) | E(HET) | P |
| --- | --- | --- | --- | --- | --- | --- | --- | --- |
| 8 | rs7825118 | ALL | A | G | 136/880/1405 | 0.3635 | 0.3626 | 0.9553 |
| 8 | rs7825118 | AFF | A | G | 47/319/530 | 0.356 | 0.3547 | 1 |
| 8 | rs7825118 | UNAFF | A | G | 89/561/875 | 0.3679 | 0.3672 | 1 |
| 8 | rs6981424 | ALL | A | G | 117/850/1460 | 0.3502 | 0.3469 | 0.6821 |
| 8 | rs6981424 | AFF | A | G | 48/322/529 | 0.3582 | 0.3569 | 1 |
| 8 | rs6981424 | UNAFF | A | G | 69/528/931 | 0.3455 | 0.3409 | 0.6524 |
| 8 | rs9297758 | ALL | A | G | 353/1167/906 | 0.481 | 0.474 | 0.4931 |
| 8 | rs9297758 | AFF | A | G | 130/420/346 | 0.4688 | 0.4709 | 0.8873 |
| 8 | rs9297758 | UNAFF | A | G | 223/747/560 | 0.4882 | 0.4757 | 0.3333 |
| 8 | rs7836840 | ALL | C | A | 457/1220/753 | 0.5021 | 0.4926 | 0.3649 |
| 8 | rs7836840 | AFF | C | A | 178/469/252 | 0.5217 | 0.4966 | 0.1398 |
| 8 | rs7836840 | UNAFF | C | A | 279/751/501 | 0.4905 | 0.4895 | 0.9584 |
