# Supplementary figures and images for: Significance of LncRNA CASC8 genetic polymorphisms on the tuberculosis susceptibility in Chinese population
Source: J Clin Lab Anal. 2020 Feb 7;34(6):e23234. doi: 10.1002/jcla.23234 (PMC7307370; doi:10.1002/jcla.23234)

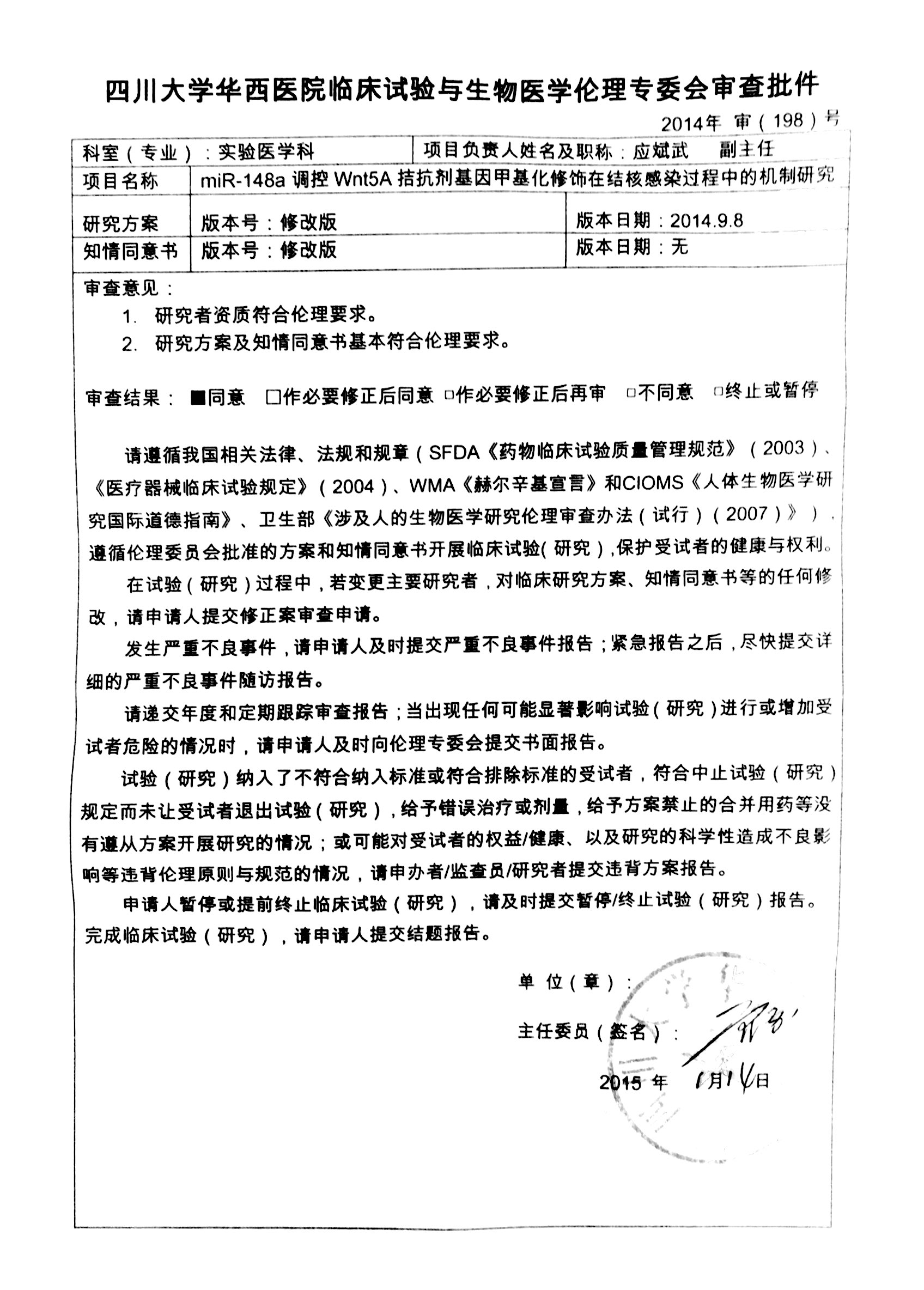

Supplement: Supplementary file 3 [file JCLA-34-e23234-s003.tif]
