# Supplementary material for: Significance of LncRNA CASC8 genetic polymorphisms on the tuberculosis susceptibility in Chinese population
Source: J Clin Lab Anal. 2020 Feb 7;34(6):e23234. doi: 10.1002/jcla.23234 (PMC7307370; doi:10.1002/jcla.23234)
Supplement: Supplementary file 4 [file JCLA-34-e23234-s004.docx]

| Detailed clinical data of TB cases | | | | | | | | |
| --- | --- | --- | --- | --- | --- | --- | --- | --- |
| **Sample ID** | **Sex** | **Age (years)** | | **TB clinical subtype** | **NEU (×10^9^/L)** | **Hb (g/L)** | **ALT (IU/L)** | **AST (IU/L)** |
| ZBYBWS01P03A02 | male | 58 | PTB | | 5.17 | 122 | 10 | 18 |
| ZBYBWS01P03A03 | male | 28 | PTB | | 8.63 | 157 | 14 | 46 |
| ZBYBWS01P03A08 | female | 33 | PTB | | 2.76 | 140 | 22 | 28 |
| ZBYBWS01P03A11 | male | 79 | PTB | | 2.06 | 112 | 20 | 66 |
| ZBYBWS01P03B01 | female | 60 | PTB & EPTB | | 3.51 | 127 | 13 | 15 |
| ZBYBWS01P03B02 | female | 77 | PTB | | 6.7 | 94 | 8 | 11 |
| ZBYBWS01P03B03 | male | 20 | PTB | | 13.26 | 134 | 17 | 16 |
| ZBYBWS01P03B07 | male | 30 | PTB & EPTB | | 7.63 | 134 | 11 | 12 |
| ZBYBWS01P03B08 | female | 48 | PTB | | 7.73 | 120 | 9 | 15 |
| ZBYBWS01P03B09 | male | 24 | PTB | | 4.62 | 142 | 28 | 29 |
| ZBYBWS01P03B11 | male | 20 | PTB | | 3.11 | 139 | 44 | 29 |
| ZBYBWS01P03C01 | male | 33 | PTB | | 6.42 | 140 | 13 | 17 |
| ZBYBWS01P03C02 | male | 28 | PTB | | 5.64 | 124 | 41 | 44 |
| ZBYBWS01P03C03 | female | 24 | PTB | | 4.93 | 128 | 17 | 22 |
| ZBYBWS01P03C05 | male | 42 | PTB | | 4.78 | 175 | 16 | 17 |
| ZBYBWS01P03C08 | female | 42 | EPTB | | NA | NA | NA | NA |
| ZBYBWS01P03C09 | male | 50 | PTB | | 4.63 | 103 | 21 | 35 |
| ZBYBWS01P03C12 | male | 20 | PTB & EPTB | | 4.34 | 161 | 7 | 15 |
| ZBYBWS01P03D01 | female | 26 | PTB & EPTB | | 10.35 | 111 | 9 | 31 |
| ZBYBWS01P03D02 | male | 73 | PTB | | 3.66 | 100 | 8 | 19 |
| ZBYBWS01P03D03 | male | 64 | PTB | | 1.98 | 96 | 9 | 20 |
| ZBYBWS01P03D05 | male | 25 | PTB & EPTB | | 5.15 | 156 | 11 | 22 |
| ZBYBWS01P03D06 | male | 42 | PTB & EPTB | | 6.8 | 124 | 35 | 24 |
| ZBYBWS01P03D07 | female | 68 | PTB & EPTB | | 2.93 | 71 | 38 | 25 |
| ZBYBWS01P03D08 | male | 56 | PTB | | 3.52 | 113 | 21 | 40 |
| ZBYBWS01P03D09 | female | 22 | PTB & EPTB | | 1.77 | 117 | 10 | 20 |
| ZBYBWS01P03E02 | male | 66 | EPTB | | 7.19 | 134 | 41 | 34 |
| ZBYBWS01P03E03 | male | 34 | PTB | | 2.47 | 118 | 9 | 19 |
| ZBYBWS01P03E04 | female | 50 | PTB | | 3.23 | 118 | 33 | 37 |
| ZBYBWS01P03E05 | female | 70 | PTB | | 2.99 | 135 | 10 | 24 |
| ZBYBWS01P03E06 | male | 17 | PTB & EPTB | | 4.69 | 127 | 14 | 15 |
| ZBYBWS01P03E07 | female | 47 | PTB & EPTB | | 6.46 | 108 | 24 | 14 |
| ZBYBWS01P03E08 | male | 29 | PTB | | 3.01 | 132 | 5 | 14 |
| ZBYBWS01P03E09 | male | 37 | PTB | | 2.48 | 135 | 16 | 17 |
| ZBYBWS01P03F04 | male | 42 | PTB & EPTB | | 3.24 | 124 | 13 | 20 |
| ZBYBWS01P03F05 | female | 33 | PTB & EPTB | | 7.56 | 99 | 16 | 15 |
| ZBYBWS01P03F11 | male | 54 | PTB | | 5.73 | 154 | 23 | 24 |
| ZBYBWS01P03F12 | male | 58 | PTB | | 12.77 | 112 | 23 | 122 |
| ZBYBWS01P03G01 | male | 26 | PTB & EPTB | | 6.24 | 99 | 45 | 29 |
| ZBYBWS01P03G03 | female | 64 | PTB & EPTB | | 4.78 | 94 | 10 | 28 |
| ZBYBWS01P03G08 | female | 18 | PTB | | 3.16 | 110 | 16 | 25 |
| ZBYBWS01P03G09 | female | 70 | PTB | | 19.08 | 132 | 22 | 38 |
| ZBYBWS01P03G11 | male | 37 | PTB | | 5.47 | 119 | 54 | 39 |
| ZBYBWS01P03H03 | female | 28 | EPTB | | 4.04 | 124 | 42 | 41 |
| ZBYBWS01P03H04 | female | 28 | PTB | | 2.79 | 121 | 66 | 85 |
| ZBYBWS01P03H10 | male | 47 | PTB | | 2.24 | 72 | 7 | 13 |
| ZBYBWS01P03H12 | male | 34 | PTB & EPTB | | 9.06 | 152 | 21 | 18 |
| ZBYBWS01P04A02 | male | 18 | PTB | | 3.25 | 135 | 118 | 70 |
| ZBYBWS01P04A03 | female | 38 | PTB | | 4.24 | 110 | 12 | 22 |
| ZBYBWS01P04A04 | male | 60 | PTB | | 9.28 | 128 | 23 | 75 |
| ZBYBWS01P04A05 | male | 63 | PTB & EPTB | | 3.02 | 90 | 25 | 41 |
| ZBYBWS01P04A06 | female | 58 | EPTB | | 3.75 | 134 | 15 | 25 |
| ZBYBWS01P04A08 | male | 21 | PTB & EPTB | | 7.27 | 146 | 71 | 27 |
| ZBYBWS01P04A10 | female | 39 | PTB & EPTB | | 8.64 | 150 | 60 | 35 |
| ZBYBWS01P04A12 | female | 59 | PTB | | 4.88 | 105 | 11 | 16 |
| ZBYBWS01P04B02 | female | 64 | PTB | | 4.71 | 103 | 26 | 27 |
| ZBYBWS01P04B05 | female | 25 | PTB & EPTB | | 3.83 | 100 | 8 | 17 |
| ZBYBWS01P04B06 | male | 51 | EPTB | | 5.69 | 151 | 46 | 28 |
| ZBYBWS01P04B07 | female | 47 | PTB | | 2.89 | 104 | 12 | 15 |
| ZBYBWS01P04B09 | female | 41 | PTB | | 3.65 | 111 | 57 | 38 |
| ZBYBWS01P04B10 | female | 21 | EPTB | | 4.39 | 92 | 22 | 23 |
| ZBYBWS01P04B11 | female | 28 | PTB | | 4.28 | 130 | 20 | 27 |
| ZBYBWS01P04B12 | male | 26 | PTB & EPTB | | 7.01 | 120 | 33 | 24 |
| ZBYBWS01P04C02 | female | 20 | PTB | | 2.43 | 110 | 14 | 20 |
| ZBYBWS01P04C05 | male | 47 | PTB | | 4.68 | 149 | 24 | 19 |
| ZBYBWS01P04C08 | female | 22 | PTB & EPTB | | 3.42 | 136 | 14 | 20 |
| ZBYBWS01P04C09 | male | 59 | PTB | | NA | 145 | NA | NA |
| ZBYBWS01P04D01 | male | 46 | PTB | | 4.8 | 128 | 26 | 17 |
| ZBYBWS01P04D02 | male | 14 | PTB & EPTB | | 1.21 | 100 | 16 | 37 |
| ZBYBWS01P04D03 | male | 45 | PTB | | 3.47 | 137 | 9 | 14 |
| ZBYBWS01P04D04 | male | 17 | EPTB | | 2.63 | 163 | 16 | 22 |
| ZBYBWS01P04D05 | female | 14 | PTB & EPTB | | 6.73 | 125 | 11 | 13 |
| ZBYBWS01P04D06 | male | 22 | PTB & EPTB | | 9.37 | 130 | 53 | 82 |
| ZBYBWS01P04D07 | male | 48 | PTB | | 6.58 | 135 | 21 | 29 |
| ZBYBWS01P04D08 | female | 47 | PTB & EPTB | | 4.97 | 85 | 19 | 16 |
| ZBYBWS01P04D10 | male | 63 | PTB | | 5.81 | 121 | 11 | 23 |
| ZBYBWS01P04D11 | female | 31 | PTB | | 4.23 | 137 | NA | NA |
| ZBYBWS01P04E01 | male | 26 | PTB & EPTB | | 4.08 | 149 | 16 | 23 |
| ZBYBWS01P04E03 | female | 23 | EPTB | | NA | 134 | NA | NA |
| ZBYBWS01P04E04 | male | 55 | PTB | | NA | 140 | NA | NA |
| ZBYBWS01P04E05 | female | 21 | PTB | | 2.48 | 119 | 29 | 33 |
| ZBYBWS01P04E07 | male | 49 | EPTB | | NA | 133 | NA | NA |
| ZBYBWS01P04E09 | male | 60 | PTB | | 9.12 | 129 | 80 | 94 |
| ZBYBWS01P04F07 | male | 60 | EPTB | | 4.51 | 139 | 14 | 21 |
| ZBYBWS01P04F10 | female | 52 | PTB & EPTB | | 8.91 | 122 | 10 | 16 |
| ZBYBWS01P04G01 | male | 18 | EPTB | | 9.77 | 153 | 38 | 29 |
| ZBYBWS01P04G02 | female | 74 | PTB | | 5.87 | 104 | 7 | 8 |
| ZBYBWS01P04G05 | female | 30 | PTB & EPTB | | 5.4 | 117 | 13 | 19 |
| ZBYBWS01P04G10 | male | 88 | PTB & EPTB | | 3.38 | 120 | 15 | 15 |
| ZBYBWS01P04H03 | female | 45 | PTB & EPTB | | 15.18 | 122 | 18 | 19 |
| ZBYBWS01P04H05 | female | 54 | PTB | | 11.38 | 103 | 21 | 42 |
| ZBYBWS01P04H06 | male | 54 | PTB | | NA | 126 | NA | NA |
| ZBYBWS01P04H07 | male | 47 | PTB & EPTB | | 6.36 | 139 | 18 | 28 |
| ZBYBWS01P04H09 | male | 48 | PTB | | 14.5 | 79 | 9 | 10 |
| ZBYBWS01P04H10 | female | 20 | PTB | | 1.73 | 123 | 19 | 20 |
| ZBYBWS01P04H11 | male | 53 | PTB | | NA | 118 | NA | NA |
| ZBYBWS01P04H12 | male | 40 | PTB | | NA | NA | NA | NA |
| ZBYBWS01P07A02 | female | 35 | EPTB | | 5.4 | 106 | 12 | 24 |
| ZBYBWS01P07A05 | female | 28 | PTB | | 4.84 | 132 | 66 | 43 |
| ZBYBWS01P07A07 | male | 76 | PTB | | 5.5 | 90 | 19 | 28 |
| ZBYBWS01P07A08 | female | 69 | PTB & EPTB | | 3.47 | 92 | 19 | 32 |
| ZBYBWS01P07A09 | male | 62 | PTB | | 4.19 | 91 | 22 | 41 |
| ZBYBWS01P07A10 | male | 62 | PTB | | NA | 127 | 41 | 70 |
| ZBYBWS01P07A11 | male | 45 | PTB | | NA | 93 | 4 | 39 |
| ZBYBWS01P07A12 | female | 53 | PTB | | 3.1 | 124 | 22 | 25 |
| ZBYBWS01P07B01 | male | 38 | PTB | | NA | 62 | 67 | 88 |
| ZBYBWS01P07B02 | male | 50 | PTB | | 4.43 | 107 | 36 | 26 |
| ZBYBWS01P07B06 | male | 59 | PTB | | NA | 132 | 26 | 16 |
| ZBYBWS01P07B07 | female | 31 | EPTB | | 6.37 | 88 | 4 | 11 |
| ZBYBWS01P07B08 | male | 29 | PTB | | 3.62 | 149 | 43 | 27 |
| ZBYBWS01P07B09 | male | 40 | PTB | | 3.26 | 117 | 16 | 21 |
| ZBYBWS01P07B10 | male | 54 | PTB | | 5.7 | 143 | 25 | 37 |
| ZBYBWS01P07B11 | male | 82 | PTB | | 12.48 | 102 | 22 | 24 |
| ZBYBWS01P07B12 | female | 50 | PTB | | 2.95 | 126 | 22 | 24 |
| ZBYBWS01P07C01 | male | 63 | PTB | | 4.44 | 107 | 21 | 29 |
| ZBYBWS01P07C02 | male | 46 | PTB | | 6.42 | 130 | 9 | 12 |
| ZBYBWS01P07C04 | male | 20 | PTB & EPTB | | 6.71 | 131 | 56 | 32 |
| ZBYBWS01P07C05 | male | 63 | EPTB | | 7.29 | 153 | 47 | 19 |
| ZBYBWS01P07C06 | female | 37 | PTB & EPTB | | 5.85 | 141 | 11 | 14 |
| ZBYBWS01P07C07 | female | 79 | PTB | | 4.15 | 120 | 13 | 23 |
| ZBYBWS01P07C08 | female | 21 | PTB | | 4.16 | 91 | 6 | 12 |
| ZBYBWS01P07C09 | male | 20 | PTB | | 6.55 | 138 | 14 | 16 |
| ZBYBWS01P07C10 | male | 49 | PTB | | 7.32 | 135 | 24 | 18 |
| ZBYBWS01P07C11 | female | 37 | PTB & EPTB | | 3.39 | 99 | 27 | 52 |
| ZBYBWS01P07C12 | female | 56 | PTB | | 3.29 | 107 | 9 | 14 |
| ZBYBWS01P07D02 | female | 14 | PTB | | 4.2 | 93 | 6 | 11 |
| ZBYBWS01P07D03 | female | 65 | PTB | | 2.16 | 103 | 35 | 34 |
| ZBYBWS01P07D04 | male | 62 | PTB | | 7.17 | 77 | 9 | 14 |
| ZBYBWS01P07D09 | male | 47 | PTB | | 5.95 | 100 | 44 | 39 |
| ZBYBWS01P07D10 | male | 27 | PTB | | 4.52 | 101 | 43 | 24 |
| ZBYBWS01P07D11 | male | 50 | PTB | | 13.07 | 87 | 15 | 16 |
| ZBYBWS01P07D12 | female | 41 | PTB | | 28.52 | 120 | 47 | 38 |
| ZBYBWS01P07E01 | male | 66 | PTB | | 11.12 | 123 | 78 | 28 |
| ZBYBWS01P07E02 | male | 58 | EPTB | | 17.11 | 105 | 24 | 31 |
| ZBYBWS01P07E04 | female | 19 | PTB | | NA | NA | NA | NA |
| ZBYBWS01P07E05 | female | 61 | PTB | | 4.82 | 98 | 34 | 25 |
| ZBYBWS01P07E06 | female | 45 | PTB & EPTB | | 11.83 | 145 | 32 | 20 |
| ZBYBWS01P07E08 | male | 48 | PTB | | 4.38 | 134 | 16 | 25 |
| ZBYBWS01P07E09 | male | 24 | EPTB | | 5.4 | 157 | 32 | 41 |
| ZBYBWS01P07E11 | male | 16 | EPTB | | 7.42 | 128 | 13 | 21 |
| ZBYBWS01P07E12 | female | 14 | EPTB | | NA | NA | NA | NA |
| ZBYBWS01P07F01 | male | 43 | PTB | | 2.97 | 122 | 11 | 14 |
| ZBYBWS01P07F02 | male | 43 | PTB | | 4 | 117 | 25 | 17 |
| ZBYBWS01P07F03 | male | 16 | EPTB | | 4.47 | 99 | 11 | 14 |
| ZBYBWS01P07F04 | female | 64 | EPTB | | NA | 88 | 16 | 17 |
| ZBYBWS01P07F05 | male | 58 | PTB | | 7.87 | 107 | 18 | 39 |
| ZBYBWS01P07F06 | male | 27 | PTB | | 2.68 | 120 | 20 | 18 |
| ZBYBWS01P07F09 | male | 49 | PTB | | 4.24 | 133 | 30 | 46 |
| ZBYBWS01P07F11 | female | 39 | EPTB | | 6.65 | 135 | 16 | 14 |
| ZBYBWS01P07G01 | male | 27 | PTB | | 6.93 | 114 | 9 | 15 |
| ZBYBWS01P07G03 | male | 58 | EPTB | | NA | 149 | 25 | 31 |
| ZBYBWS01P07G04 | female | 50 | PTB | | 3.32 | 97 | 6 | 8 |
| ZBYBWS01P07G08 | male | 27 | PTB | | 10.35 | 136 | 33 | 20 |
| ZBYBWS01P07G10 | female | 47 | PTB | | 5.83 | 113 | 36 | 27 |
| ZBYBWS01P07G11 | male | 25 | PTB | | 7.48 | 121 | 12 | 12 |
| ZBYBWS01P07G12 | male | 55 | PTB | | 3.09 | 126 | 10 | 19 |
| ZBYBWS01P07H01 | female | 29 | PTB | | 7.43 | 74 | 21 | 18 |
| ZBYBWS01P07H03 | male | 70 | PTB | | 6.62 | 92 | 14 | 22 |
| ZBYBWS01P07H04 | male | 74 | PTB & EPTB | | 2.26 | 101 | 26 | 23 |
| ZBYBWS01P07H05 | male | 31 | EPTB | | 5.02 | 145 | 29 | 25 |
| ZBYBWS01P07H06 | female | 30 | PTB | | 3.12 | 99 | 45 | 21 |
| ZBYBWS01P07H08 | male | 66 | PTB | | 10.75 | 111 | 6 | 14 |
| ZBYBWS01P07H09 | male | 62 | PTB | | 7.87 | 116 | 26 | 52 |
| ZBYBWS01P07H10 | male | 22 | PTB | | 11.14 | 138 | 37 | 21 |
| ZBYBWS01P07H11 | male | 57 | PTB | | 3.48 | 91 | 37 | 63 |
| ZBYBWS01P08A03 | female | 42 | EPTB | | 2.74 | 126 | 8 | 16 |
| ZBYBWS01P08A04 | male | 41 | PTB | | 4.69 | 144 | 81 | 75 |
| ZBYBWS01P08A05 | male | 80 | PTB | | NA | 116 | 12 | 12 |
| ZBYBWS01P08A06 | male | 24 | PTB | | 5.2 | 165 | 10 | 25 |
| ZBYBWS01P08A07 | female | 26 | PTB | | 2.26 | 141 | 6 | 16 |
| ZBYBWS01P08A10 | male | 40 | PTB | | 4.17 | 155 | 15 | 18 |
| ZBYBWS01P08A11 | male | 18 | PTB | | 9.88 | 158 | 10 | 15 |
| ZBYBWS01P08A12 | male | 49 | PTB | | NA | 141 | 36 | 75 |
| ZBYBWS01P08B01 | male | 87 | PTB | | 8.33 | 91 | 7 | 25 |
| ZBYBWS01P08B04 | male | 31 | PTB | | 1.71 | 146 | 29 | 25 |
| ZBYBWS01P08B05 | female | 18 | PTB | | 6.63 | 86 | 29 | 37 |
| ZBYBWS01P08B09 | male | 26 | PTB & EPTB | | 13.95 | 113 | 8 | 13 |
| ZBYBWS01P08B11 | male | 52 | PTB | | 8.13 | 127 | 18 | 42 |
| ZBYBWS01P08C02 | male | 51 | EPTB | | 2.61 | 97 | 7 | 36 |
| ZBYBWS01P08C03 | male | 23 | EPTB | | 4.13 | 136 | 21 | 18 |
| ZBYBWS01P08C05 | female | 23 | PTB | | 2.35 | 109 | 7 | 19 |
| ZBYBWS01P08C06 | female | 62 | PTB | | 2.04 | 140 | 6 | 11 |
| ZBYBWS01P08C07 | female | 25 | PTB | | 7.04 | 146 | 15 | 18 |
| ZBYBWS01P08C09 | female | 66 | PTB | | 4.07 | 106 | 21 | 29 |
| ZBYBWS01P08D01 | male | 32 | EPTB | | 4.49 | 133 | 18 | 40 |
| ZBYBWS01P08D02 | male | 58 | PTB | | 4.7 | 97 | 16 | 21 |
| ZBYBWS01P08D05 | male | 88 | PTB | | 4.12 | 135 | 11 | 26 |
| ZBYBWS01P08D07 | female | 73 | PTB | | 5.74 | 131 | 28 | 26 |
| ZBYBWS01P08D08 | male | 68 | PTB | | 10.21 | 125 | 13 | 14 |
| ZBYBWS01P08D09 | male | 47 | PTB | | 7.72 | 63 | 6 | 14 |
| ZBYBWS01P08D10 | male | 52 | PTB | | 3.45 | 101 | 14 | 24 |
| ZBYBWS01P08D11 | male | 33 | PTB | | 8.9 | 92 | 5 | 16 |
| ZBYBWS01P08E01 | male | 22 | PTB & EPTB | | 5.55 | 109 | 54 | 29 |
| ZBYBWS01P08E02 | male | 26 | EPTB | | 5.28 | 140 | 41 | 23 |
| ZBYBWS01P08E03 | male | 58 | PTB | | 3.04 | 118 | 23 | 18 |
| ZBYBWS01P08E04 | male | 45 | PTB | | 4.05 | 121 | 28 | 26 |
| ZBYBWS01P08E05 | male | 76 | PTB | | 4.39 | 95 | 10 | 18 |
| ZBYBWS01P08E08 | female | 56 | EPTB | | 3.55 | 123 | 17 | 29 |
| ZBYBWS01P08E09 | male | 79 | PTB | | 4.13 | 126 | 26 | 19 |
| ZBYBWS01P08E10 | male | 74 | EPTB | | 5.18 | 81 | 8 | 11 |
| ZBYBWS01P08E12 | female | 62 | PTB | | 3.27 | 117 | 11 | 15 |
| ZBYBWS01P08F02 | female | 24 | PTB | | 5.35 | 80 | 12 | 47 |
| ZBYBWS01P08F03 | female | 56 | PTB | | 13.18 | 102 | 35 | 34 |
| ZBYBWS01P08F04 | male | 17 | PTB | | 7.37 | 84 | 6 | 17 |
| ZBYBWS01P08F06 | female | 73 | PTB & EPTB | | 5.18 | 73 | 23 | 22 |
| ZBYBWS01P08F07 | male | 55 | PTB | | 4.21 | 112 | 21 | 45 |
| ZBYBWS01P08F09 | male | 42 | PTB | | 3.22 | 148 | 9 | 19 |
| ZBYBWS01P08F10 | female | 77 | EPTB | | 4.64 | 116 | 42 | 31 |
| ZBYBWS01P08F11 | male | 64 | PTB | | 9.68 | 95 | 17 | 22 |
| ZBYBWS01P08F12 | female | 50 | PTB | | NA | 99 | 6 | 5 |
| ZBYBWS01P08G01 | male | 56 | PTB | | 6.33 | 97 | 38 | 203 |
| ZBYBWS01P08G02 | male | 22 | PTB & EPTB | | 3.54 | 109 | 14 | 12 |
| ZBYBWS01P08G03 | male | 48 | EPTB | | 3.72 | 129 | 49 | 37 |
| ZBYBWS01P08G06 | female | 41 | PTB | | 3.44 | 128 | 17 | 18 |
| ZBYBWS01P08G07 | female | 26 | EPTB | | 4.87 | 97 | 14 | 18 |
| ZBYBWS01P08G08 | male | 56 | PTB | | 1.62 | 135 | 14 | 20 |
| ZBYBWS01P08G09 | male | 15 | PTB | | 6.24 | 144 | 6 | 16 |
| ZBYBWS01P08H03 | male | 23 | PTB | | 7.52 | 73 | 17 | 24 |
| ZBYBWS01P08H04 | male | 61 | PTB | | 5.24 | 127 | 24 | 26 |
| ZBYBWS01P08H05 | female | 40 | PTB | | 4.67 | 118 | 14 | 15 |
| ZBYBWS01P08H07 | male | 59 | PTB | | 3.97 | 78 | 5 | 15 |
| ZBYBWS01P08H09 | male | 25 | PTB | | 5.42 | 115 | 9 | 13 |
| ZBYBWS01P08H11 | male | 44 | PTB | | 8.59 | 160 | 25 | 18 |
| ZBYBWS01P11A02 | female | 24 | PTB | | 4.97 | 129 | 13 | 19 |
| ZBYBWS01P11A03 | male | 20 | EPTB | | 7.24 | 113 | 9 | 12 |
| ZBYBWS01P11A04 | male | 26 | PTB | | 3.58 | 155 | 5 | 13 |
| ZBYBWS01P11A05 | male | 61 | PTB | | 4.96 | 121 | 13 | 13 |
| ZBYBWS01P11A06 | male | 21 | PTB | | 4.92 | 134 | 68 | 63 |
| ZBYBWS01P11A09 | male | 20 | PTB | | 7.44 | 125 | 13 | 14 |
| ZBYBWS01P11A10 | female | 62 | PTB | | 5.36 | 108 | 14 | 18 |
| ZBYBWS01P11A11 | male | 29 | PTB | | 9.35 | 109 | 15 | 11 |
| ZBYBWS01P11A12 | female | 19 | EPTB | | 8.39 | 139 | 47 | 33 |
| ZBYBWS01P11B01 | male | 58 | PTB | | 3.98 | 123 | 16 | 22 |
| ZBYBWS01P11B02 | female | 58 | PTB | | 7.79 | 99 | 9 | 10 |
| ZBYBWS01P11B03 | male | 22 | PTB | | 6.93 | 118 | 9 | 11 |
| ZBYBWS01P11B05 | male | 47 | EPTB | | 6.91 | 128 | 15 | 12 |
| ZBYBWS01P11B06 | female | 27 | PTB | | 5.46 | 114 | 15 | 23 |
| ZBYBWS01P11B07 | male | 19 | PTB | | NA | 148 | 38 | 25 |
| ZBYBWS01P11B08 | female | 22 | PTB | | 2.7 | 125 | 16 | 16 |
| ZBYBWS01P11B09 | male | 69 | PTB | | 5.68 | 101 | 17 | 41 |
| ZBYBWS01P11B10 | female | 39 | PTB | | 14.37 | 99 | 1 | 5 |
| ZBYBWS01P11B11 | male | 56 | EPTB | | 3.55 | 109 | 10 | 16 |
| ZBYBWS01P11B12 | male | 59 | PTB | | 7.58 | 129 | 15 | 17 |
| ZBYBWS01P11C01 | male | 43 | PTB | | 3.41 | 147 | 15 | 20 |
| ZBYBWS01P11C02 | female | 21 | PTB | | 2.61 | 127 | 63 | 46 |
| ZBYBWS01P11C03 | male | 17 | PTB | | 7.12 | 92 | 11 | 11 |
| ZBYBWS01P11C04 | female | 35 | PTB | | 10.63 | 104 | 22 | 25 |
| ZBYBWS01P11C05 | male | 16 | EPTB | | 6.64 | 117 | 10 | 20 |
| ZBYBWS01P11C07 | female | 26 | PTB | | 5.86 | 102 | 10 | 17 |
| ZBYBWS01P11C08 | male | 33 | PTB | | NA | 147 | 28 | 27 |
| ZBYBWS01P11C10 | male | 49 | PTB | | 6.24 | 107 | 33 | 41 |
| ZBYBWS01P11C12 | female | 28 | PTB | | 3.12 | 85 | 10 | 15 |
| ZBYBWS01P11D01 | male | 18 | PTB | | 5.92 | 137 | 46 | 33 |
| ZBYBWS01P11D02 | female | 61 | PTB | | 5.84 | 129 | 10 | 26 |
| ZBYBWS01P11D03 | female | 26 | PTB | | 10.02 | 134 | 49 | 30 |
| ZBYBWS01P11D04 | male | 42 | PTB | | 5.84 | 161 | 13 | 21 |
| ZBYBWS01P11D05 | male | 40 | PTB | | 7.48 | 135 | 13 | 45 |
| ZBYBWS01P11D06 | female | 86 | PTB | | NA | 100 | 15 | 40 |
| ZBYBWS01P11D07 | female | 22 | PTB | | 4 | 126 | 13 | 12 |
| ZBYBWS01P11D08 | male | 23 | PTB | | 10.06 | 138 | 14 | 21 |
| ZBYBWS01P11D09 | male | 30 | PTB | | 11.24 | 113 | 10 | 16 |
| ZBYBWS01P11D10 | male | 40 | PTB & EPTB | | 6.17 | 104 | 20 | 24 |
| ZBYBWS01P11D11 | male | 26 | PTB | | 9.37 | 108 | 9 | 17 |
| ZBYBWS01P11D12 | female | 28 | PTB | | 4.77 | 123 | 11 | 18 |
| ZBYBWS01P11E02 | male | 82 | PTB | | NA | 63 | 32 | 12 |
| ZBYBWS01P11E03 | female | 24 | PTB & EPTB | | 7.19 | 127 | 15 | 26 |
| ZBYBWS01P11E04 | male | 66 | PTB | | 8.55 | 79 | 17 | 88 |
| ZBYBWS01P11E05 | female | 50 | PTB & EPTB | | 3.04 | 138 | 28 | 18 |
| ZBYBWS01P11E06 | male | 60 | PTB & EPTB | | 3.38 | 93 | 28 | 31 |
| ZBYBWS01P11E07 | female | 57 | PTB & EPTB | | 5.68 | 143 | 23 | 24 |
| ZBYBWS01P11E08 | male | 57 | PTB | | 9.86 | 100 | 14 | 23 |
| ZBYBWS01P11E09 | female | 72 | PTB | | 6.56 | 103 | 11 | 19 |
| ZBYBWS01P11E10 | female | 30 | EPTB | | 1.32 | 110 | 46 | 37 |
| ZBYBWS01P11E11 | male | 35 | PTB | | 18.45 | 108 | 36 | 50 |
| ZBYBWS01P11E12 | male | 64 | PTB | | 9.14 | 101 | 11 | 15 |
| ZBYBWS01P11F01 | female | 72 | PTB | | 3.11 | 119 | 23 | 38 |
| ZBYBWS01P11F02 | female | 63 | PTB | | 5.01 | 118 | 15 | 42 |
| ZBYBWS01P11F03 | female | 68 | PTB | | 3.84 | 105 | 9 | 14 |
| ZBYBWS01P11F04 | male | 65 | PTB | | 6.65 | 128 | 13 | 18 |
| ZBYBWS01P11F05 | female | 64 | PTB | | 11.87 | 122 | 8 | 16 |
| ZBYBWS01P11F08 | female | 22 | PTB & EPTB | | 6.91 | 131 | 12 | 13 |
| ZBYBWS01P11F10 | female | 22 | PTB & EPTB | | 9.39 | 108 | 17 | 25 |
| ZBYBWS01P11F12 | male | 29 | EPTB | | 9.23 | NA | 48 | 37 |
| ZBYBWS01P11G01 | female | 32 | EPTB | | 4.25 | 93 | 12 | 18 |
| ZBYBWS01P11G02 | male | 47 | PTB | | 5.85 | 173 | 18 | 17 |
| ZBYBWS01P11G03 | female | 53 | PTB | | 3.94 | 90 | 48 | 73 |
| ZBYBWS01P11G04 | male | 22 | PTB | | 3.51 | 126 | 26 | 24 |
| ZBYBWS01P11G05 | male | 69 | PTB | | 3.84 | 126 | 93 | 66 |
| ZBYBWS01P11G06 | male | 25 | EPTB | | 5.11 | 152 | 23 | 13 |
| ZBYBWS01P11G07 | male | 57 | PTB | | 5.21 | 126 | 23 | 11 |
| ZBYBWS01P11G10 | male | 56 | PTB | | 6.7 | 121 | 14 | 15 |
| ZBYBWS01P11G11 | female | 52 | EPTB | | 4.46 | 90 | 71 | 67 |
| ZBYBWS01P11G12 | female | 63 | PTB | | 4.83 | 97 | 17 | 23 |
| ZBYBWS01P11H01 | male | 48 | PTB | | 5.96 | 166 | 11 | 17 |
| ZBYBWS01P11H02 | female | 42 | EPTB | | 2.98 | 127 | 11 | 90 |
| ZBYBWS01P11H03 | female | 16 | PTB | | 2.39 | 142 | 7 | 18 |
| ZBYBWS01P11H04 | female | 52 | PTB | | 2.86 | 136 | 14 | 24 |
| ZBYBWS01P11H06 | female | 40 | PTB & EPTB | | 5.13 | 118 | 15 | 17 |
| ZBYBWS01P11H09 | male | 27 | PTB | | 11.49 | 149 | 43 | 35 |
| ZBYBWS01P12A04 | female | 32 | PTB | | 6.06 | 120 | 13 | 20 |
| ZBYBWS01P12A12 | female | 30 | PTB | | 4.8 | 118 | 19 | 42 |
| ZBYBWS01P12B02 | female | 44 | PTB & EPTB | | 6.97 | 136 | 34 | 40 |
| ZBYBWS01P12B04 | male | 71 | PTB | | 4.13 | 92 | 58 | 40 |
| ZBYBWS01P12B05 | male | 58 | PTB | | 7.29 | 121 | 8 | 15 |
| ZBYBWS01P12B06 | female | 22 | PTB | | 3.26 | 119 | 9 | 16 |
| ZBYBWS01P12B08 | female | 41 | PTB | | 5.47 | 111 | 18 | 15 |
| ZBYBWS01P12C01 | male | 66 | PTB | | 10.24 | 108 | 30 | 49 |
| ZBYBWS01P12C02 | male | 20 | PTB | | 5.85 | 143 | 10 | 22 |
| ZBYBWS01P12C03 | female | 17 | PTB | | 2.75 | 111 | 12 | 12 |
| ZBYBWS01P12C05 | female | 62 | PTB | | 4.64 | 96 | 15 | 19 |
| ZBYBWS01P12C07 | female | 32 | PTB | | 18.68 | 107 | 35 | 49 |
| ZBYBWS01P12C08 | male | 49 | PTB | | 4.14 | 131 | 18 | 18 |
| ZBYBWS01P12C09 | female | 33 | PTB | | 5.32 | 125 | 14 | 18 |
| ZBYBWS01P12C11 | male | 30 | PTB | | 5.51 | 142 | 27 | 26 |
| ZBYBWS01P12D01 | male | 81 | PTB | | 9.5 | 137 | 58 | 93 |
| ZBYBWS01P12D03 | male | 24 | PTB & EPTB | | 3.89 | 109 | 12 | 25 |
| ZBYBWS01P12D05 | male | 48 | PTB | | 6.04 | 60 | 4 | 12 |
| ZBYBWS01P12D06 | male | 51 | EPTB | | 3.02 | NA | 39 | 27 |
| ZBYBWS01P12D07 | male | 56 | PTB | | 7.61 | 93 | 26 | 32 |
| ZBYBWS01P12D11 | male | 69 | PTB | | 4.91 | 122 | 4 | 13 |
| ZBYBWS01P12D12 | male | 17 | PTB | | 4.66 | 148 | 21 | 23 |
| ZBYBWS01P12E01 | male | 47 | PTB | | 1.2 | 109 | 7 | 18 |
| ZBYBWS01P12E02 | female | 30 | PTB | | 5.27 | 119 | 49 | 52 |
| ZBYBWS01P12E03 | male | 33 | PTB | | 4.18 | 114 | 42 | 51 |
| ZBYBWS01P12E04 | female | 70 | PTB | | 10.93 | 95 | 17 | 20 |
| ZBYBWS01P12E05 | male | 41 | PTB | | 6.26 | 76 | 27 | 26 |
| ZBYBWS01P12E06 | male | 32 | PTB | | 4.18 | 139 | 16 | 20 |
| ZBYBWS01P12E07 | female | 73 | PTB | | 5.98 | 87 | 8 | 13 |
| ZBYBWS01P12E08 | male | 24 | PTB | | 3.23 | 144 | 33 | 39 |
| ZBYBWS01P12E10 | male | 22 | PTB | | 3.78 | 117 | 4 | 102 |
| ZBYBWS01P12E11 | female | 32 | PTB | | 2.57 | 121 | 9 | 14 |
| ZBYBWS01P12E12 | female | 33 | PTB | | 4.53 | 135 | 23 | 19 |
| ZBYBWS01P12F01 | male | 66 | PTB | | 1.39 | 137 | 12 | 20 |
| ZBYBWS01P12F02 | female | 69 | PTB | | 2.34 | 115 | 16 | 25 |
| ZBYBWS01P12F03 | female | 27 | PTB | | 5.09 | 127 | 9 | 15 |
| ZBYBWS01P12F04 | male | 39 | PTB | | 3.99 | 81 | 52 | 64 |
| ZBYBWS01P12F05 | male | 72 | PTB | | 5.27 | 95 | 5 | 15 |
| ZBYBWS01P12F06 | female | 40 | PTB & EPTB | | 5.2 | 114 | 35 | 47 |
| ZBYBWS01P12F07 | female | 27 | EPTB | | 3.46 | 145 | 22 | 15 |
| ZBYBWS01P12F08 | female | 43 | PTB | | 4.55 | 100 | 5 | 11 |
| ZBYBWS01P12F09 | male | 51 | PTB | | 3.87 | 114 | NA | NA |
| ZBYBWS01P12F11 | female | 49 | PTB | | 3.93 | 130 | 7 | 10 |
| ZBYBWS01P12F12 | female | 57 | EPTB | | 4.22 | 123 | 20 | 23 |
| ZBYBWS01P12G02 | male | 57 | PTB & EPTB | | 4.04 | 113 | 25 | 36 |
| ZBYBWS01P12G03 | female | 21 | EPTB | | 3.27 | 134 | 17 | 17 |
| ZBYBWS01P12G04 | male | 56 | PTB | | 3.51 | 128 | 43 | 96 |
| ZBYBWS01P12G05 | male | 41 | PTB | | 5.89 | 142 | 45 | 32 |
| ZBYBWS01P12G06 | female | 15 | PTB & EPTB | | 4.8 | 132 | 8 | 13 |
| ZBYBWS01P12G07 | female | 24 | PTB & EPTB | | 3.29 | 116 | 9 | 7 |
| ZBYBWS01P12G08 | female | 54 | PTB | | 5.86 | 98 | 9 | 23 |
| ZBYBWS01P12G09 | male | 18 | PTB & EPTB | | 3.42 | 141 | 16 | 14 |
| ZBYBWS01P12G10 | female | 14 | PTB & EPTB | | 8.53 | 116 | 26 | 22 |
| ZBYBWS01P12G11 | male | 16 | PTB | | 3.8 | 122 | 20 | 24 |
| ZBYBWS01P12H01 | female | 15 | PTB | | 3.29 | 136 | 12 | 27 |
| ZBYBWS01P12H02 | male | 25 | PTB | | 3.98 | 125 | 8 | 19 |
| ZBYBWS01P12H03 | male | 50 | PTB | | 4.21 | 143 | 13 | 8 |
| ZBYBWS01P12H04 | male | 48 | EPTB | | 8.74 | 112 | 15 | 29 |
| ZBYBWS01P12H05 | male | 59 | PTB | | 4.6 | 107 | 12 | 17 |
| ZBYBWS01P12H06 | male | 46 | PTB | | 5.7 | 148 | 21 | 17 |
| ZBYBWS01P12H09 | male | 55 | PTB | | 2.76 | 127 | 12 | 18 |
| ZBYBWS01P12H10 | female | 20 | PTB & EPTB | | 10.58 | 104 | 6 | 11 |
| ZBYBWS01P15A03 | female | 32 | PTB & EPTB | | 2.98 | 113 | 18 | 18 |
| ZBYBWS01P15A04 | male | 68 | PTB & EPTB | | 4.25 | 103 | 12 | 28 |
| ZBYBWS01P15A05 | female | 44 | EPTB | | 2.37 | 127 | 16 | 25 |
| ZBYBWS01P15A07 | male | 79 | PTB | | 3.88 | 126 | 41 | 32 |
| ZBYBWS01P15A10 | male | 15 | PTB & EPTB | | 13.44 | 102 | 7 | 12 |
| ZBYBWS01P15B02 | male | 60 | EPTB | | 5.57 | 110 | 11 | 15 |
| ZBYBWS01P15B06 | male | 48 | PTB & EPTB | | 4.53 | 142 | 65 | 37 |
| ZBYBWS01P15B07 | female | 41 | PTB | | 1.85 | 109 | 19 | 22 |
| ZBYBWS01P15B11 | male | 49 | EPTB | | 6.54 | 100 | 19 | 17 |
| ZBYBWS01P15B12 | male | 61 | EPTB | | NA | 88 | NA | NA |
| ZBYBWS01P15C01 | male | 71 | PTB | | 3.76 | 97 | 6 | 12 |
| ZBYBWS01P15C02 | female | 49 | PTB | | NA | 141 | NA | NA |
| ZBYBWS01P15C03 | male | 50 | EPTB | | 1.89 | 129 | 19 | 18 |
| ZBYBWS01P15C05 | male | 20 | PTB & EPTB | | 6.42 | 92 | 55 | 28 |
| ZBYBWS01P15C06 | female | 51 | EPTB | | 3.19 | 129 | 12 | 20 |
| ZBYBWS01P15C08 | female | 54 | PTB & EPTB | | 4.02 | 100 | 25 | 30 |
| ZBYBWS01P15C09 | female | 44 | PTB | | 6.23 | 64 | 9 | 26 |
| ZBYBWS01P15D01 | female | 31 | PTB | | 3.81 | 115 | 30 | 31 |
| ZBYBWS01P15D03 | male | 62 | PTB | | 3.71 | 127 | 22 | 17 |
| ZBYBWS01P15D08 | male | 53 | PTB | | 2.51 | 163 | 18 | 21 |
| ZBYBWS01P15D12 | female | 57 | PTB | | 6.18 | 130 | 22 | 50 |
| ZBYBWS01P15E01 | female | 47 | PTB | | 3.95 | 104 | 10 | 14 |
| ZBYBWS01P15E09 | female | 22 | PTB | | 4.28 | 135 | 20 | 30 |
| ZBYBWS01P15E10 | female | 57 | PTB | | 1.11 | 99 | 47 | 56 |
| ZBYBWS01P15E11 | female | 18 | PTB & EPTB | | NA | 118 | 14 | 6 |
| ZBYBWS01P15E12 | male | 27 | PTB & EPTB | | 7.44 | 128 | 17 | 21 |
| ZBYBWS01P15F01 | male | 18 | PTB & EPTB | | 3.98 | 122 | 15 | 13 |
| ZBYBWS01P15F02 | male | 22 | PTB | | 2.7 | 149 | 14 | 21 |
| ZBYBWS01P15F03 | female | 27 | PTB | | 4.55 | 141 | 12 | 34 |
| ZBYBWS01P15F04 | male | 25 | PTB & EPTB | | 4.63 | 123 | 57 | 24 |
| ZBYBWS01P15F05 | male | 31 | PTB | | 13.59 | 122 | 5 | 13 |
| ZBYBWS01P15F06 | female | 41 | PTB | | NA | 139 | 20 | 20 |
| ZBYBWS01P15F10 | male | 31 | EPTB | | 2.62 | 95 | 6 | 12 |
| ZBYBWS01P15F11 | male | 17 | PTB | | 7.53 | 94 | 28 | 48 |
| ZBYBWS01P15F12 | female | 24 | PTB | | 2.56 | 139 | 7 | 17 |
| ZBYBWS01P15G01 | female | 45 | PTB & EPTB | | 3.4 | 131 | 17 | 26 |
| ZBYBWS01P15G02 | female | 14 | EPTB | | NA | NA | NA | NA |
| ZBYBWS01P15G03 | female | 76 | EPTB | | 2.99 | NA | 11 | 14 |
| ZBYBWS01P15G04 | male | 14 | PTB | | NA | 126 | 26 | 38 |
| ZBYBWS01P15G05 | male | 37 | PTB | | 4.32 | 152 | 20 | 17 |
| ZBYBWS01P15G08 | female | 52 | PTB | | 4.83 | 118 | 16 | 20 |
| ZBYBWS01P15G09 | female | 37 | PTB | | 5.74 | 128 | 8 | 17 |
| ZBYBWS01P15G11 | male | 61 | PTB | | 6.88 | 139 | 35 | 31 |
| ZBYBWS01P15H02 | female | 27 | PTB & EPTB | | 3.92 | 121 | 12 | 12 |
| ZBYBWS01P15H03 | male | 20 | PTB | | 10.64 | 101 | 7 | 16 |
| ZBYBWS01P15H04 | male | 35 | EPTB | | 4.64 | 111 | 36 | 38 |
| ZBYBWS01P15H06 | male | 39 | PTB | | 6.92 | 135 | 10 | 17 |
| ZBYBWS01P15H07 | male | 36 | PTB | | 8.38 | 138 | 12 | 15 |
| ZBYBWS01P15H08 | male | 52 | PTB | | 1.99 | 132 | 12 | 14 |
| ZBYBWS01P15H09 | male | 29 | PTB | | 4.43 | 122 | 11 | 19 |
| ZBYBWS01P15H11 | female | 44 | PTB | | 2.84 | 111 | 21 | 15 |
| ZBYBWS01P16A02 | female | 65 | PTB | | 3.05 | 111 | 17 | 18 |
| ZBYBWS01P16A04 | male | 36 | PTB | | 2.03 | 142 | 37 | 39 |
| ZBYBWS01P16A09 | male | 22 | EPTB | | 9.61 | 82 | 8 | 19 |
| ZBYBWS01P16A10 | male | 86 | PTB | | 12.97 | 103 | 12 | 17 |
| ZBYBWS01P16A12 | female | 60 | EPTB | | 10.87 | 101 | 15 | 23 |
| ZBYBWS01P16B01 | female | 80 | EPTB | | 1.51 | 92 | 77 | 38 |
| ZBYBWS01P16B02 | male | 23 | PTB | | 5.06 | 149 | 43 | 27 |
| ZBYBWS01P16B03 | male | 27 | PTB & EPTB | | 14.42 | 129 | 20 | 27 |
| ZBYBWS01P16B04 | male | 19 | PTB | | 6.82 | 122 | 14 | 21 |
| ZBYBWS01P16B05 | male | 47 | PTB | | 4.28 | 148 | 38 | 29 |
| ZBYBWS01P16B06 | female | 51 | PTB | | 1.34 | 133 | 7 | 12 |
| ZBYBWS01P16B07 | male | 18 | EPTB | | 8.02 | 129 | 45 | 23 |
| ZBYBWS01P16B08 | female | 48 | PTB & EPTB | | 1.68 | 130 | 8 | 15 |
| ZBYBWS01P16B09 | male | 45 | PTB & EPTB | | 1.87 | 105 | 49 | 51 |
| ZBYBWS01P16B10 | female | 43 | PTB | | 5.59 | 138 | 12 | 16 |
| ZBYBWS01P16B11 | female | 29 | PTB | | 11.18 | 131 | 11 | 17 |
| ZBYBWS01P16B12 | male | 63 | PTB | | 5.52 | 153 | 10 | 31 |
| ZBYBWS01P16C02 | male | 72 | PTB | | NA | 118 | 18 | 11 |
| ZBYBWS01P16C08 | male | 52 | PTB | | 4.6 | 141 | 13 | 10 |
| ZBYBWS01P16C09 | male | 62 | PTB | | 3.36 | NA | 14 | 17 |
| ZBYBWS01P16C10 | male | 24 | PTB | | 9.06 | 116 | 45 | 28 |
| ZBYBWS01P16C11 | male | 19 | EPTB | | 2.89 | 113 | 38 | 46 |
| ZBYBWS01P16D03 | male | 61 | PTB | | 8.63 | 184 | 18 | 26 |
| ZBYBWS01P16D05 | male | 78 | PTB | | 3.18 | 103 | 34 | 52 |
| ZBYBWS01P16D07 | female | 29 | EPTB | | NA | 134 | 276 | 282 |
| ZBYBWS01P16D08 | male | 45 | PTB | | 3.91 | 142 | 15 | 21 |
| ZBYBWS01P16E01 | male | 21 | PTB | | 5.05 | 77 | 4 | 11 |
| ZBYBWS01P16E04 | female | 61 | PTB | | 3.75 | 128 | 16 | 20 |
| ZBYBWS01P16E06 | male | 22 | PTB | | 12.14 | 109 | 8 | 16 |
| ZBYBWS01P16E07 | male | 57 | PTB & EPTB | | 2.78 | 111 | 64 | 51 |
| ZBYBWS01P16E08 | male | 52 | PTB | | 2.41 | 84 | 35 | 30 |
| ZBYBWS01P16F04 | female | 68 | PTB | | 2.88 | 118 | 5 | 15 |
| ZBYBWS01P16F05 | female | 46 | EPTB | | 3.81 | 102 | 6 | 20 |
| ZBYBWS01P16F07 | female | 42 | PTB | | 6.85 | 99 | 21 | 28 |
| ZBYBWS01P16F08 | female | 31 | PTB & EPTB | | 6.75 | 102 | 18 | 98 |
| ZBYBWS01P16F11 | male | 44 | PTB | | 5.41 | 108 | 10 | 15 |
| ZBYBWS01P16F12 | male | 22 | PTB & EPTB | | 7.63 | 150 | 51 | 30 |
| ZBYBWS01P16G01 | male | 46 | PTB | | 2.65 | 136 | 18 | 19 |
| ZBYBWS01P16G03 | female | 28 | PTB | | 2.1 | 125 | 33 | 22 |
| ZBYBWS01P16G05 | male | 63 | PTB & EPTB | | 4.63 | 68 | 7 | 40 |
| ZBYBWS01P16G07 | female | 25 | PTB | | 1.66 | 139 | 48 | 36 |
| ZBYBWS01P16G09 | female | 49 | PTB & EPTB | | 2.46 | NA | 17 | 23 |
| ZBYBWS01P16G10 | male | 39 | PTB | | 12.07 | 169 | 44 | 35 |
| ZBYBWS01P16G12 | male | 64 | EPTB | | 8.88 | NA | 5 | 9 |
| ZBYBWS01P16H01 | female | 64 | PTB | | 2.53 | 119 | 38 | 40 |
| ZBYBWS01P16H02 | male | 54 | PTB | | 2.61 | NA | 18 | 27 |
| ZBYBWS01P16H03 | male | 76 | PTB | | 3.89 | 130 | 14 | 25 |
| ZBYBWS01P16H04 | male | 61 | PTB | | 5.36 | 156 | 16 | 15 |
| ZBYBWS01P16H06 | male | 32 | PTB | | 1.81 | 79 | 167 | 64 |
| ZBYBWS01P16H07 | male | 42 | PTB | | 5.34 | 105 | 40 | 19 |
| ZBYBWS01P16H08 | female | 31 | PTB & EPTB | | 5.62 | 114 | 12 | 14 |
| ZBYBWS01P16H09 | male | 18 | PTB | | NA | 161 | NA | NA |
| ZBYBWS01P16H10 | male | 58 | PTB | | NA | 165 | NA | NA |
| ZBYBWS01P16H11 | male | 20 | PTB | | 8.05 | 104 | 7 | 16 |
| ZBYBWS01P19A02 | male | 17 | EPTB | | 19.21 | 151 | 16 | 23 |
| ZBYBWS01P19A04 | male | 63 | PTB | | 3.79 | 154 | 28 | 36 |
| ZBYBWS01P19A06 | male | 59 | PTB | | 4.92 | 141 | 15 | 19 |
| ZBYBWS01P19A10 | female | 24 | PTB & EPTB | | 6.42 | 112 | 10 | 12 |
| ZBYBWS01P19B03 | male | 76 | PTB | | 4.65 | 112 | 30 | 44 |
| ZBYBWS01P19B08 | male | 25 | PTB | | 13.86 | 97 | 9 | 19 |
| ZBYBWS01P19B11 | female | 49 | EPTB | | 3.7 | 102 | 32 | 20 |
| ZBYBWS01P19C01 | female | 30 | PTB | | 5.09 | 130 | 11 | 15 |
| ZBYBWS01P19C04 | male | 21 | PTB | | 8.5 | 117 | 10 | 18 |
| ZBYBWS01P19C07 | male | 19 | PTB & EPTB | | 4.25 | 120 | 8 | 14 |
| ZBYBWS01P19C08 | male | 62 | EPTB | | 4.24 | 80 | 6 | 23 |
| ZBYBWS01P19C11 | male | 14 | PTB | | 5.67 | 144 | 25 | 26 |
| ZBYBWS01P19C12 | male | 47 | PTB | | 4.65 | 114 | 46 | 73 |
| ZBYBWS01P19D01 | female | 23 | PTB & EPTB | | 5.19 | 118 | 29 | 41 |
| ZBYBWS01P19D03 | male | 35 | PTB | | 2.71 | 141 | 26 | 24 |
| ZBYBWS01P19D07 | female | 14 | EPTB | | 5.25 | 90 | 9 | 16 |
| ZBYBWS01P19D10 | male | 47 | PTB | | NA | 148 | 29 | 2 |
| ZBYBWS01P19E04 | male | 73 | PTB | | 8.65 | 123 | 18 | 18 |
| ZBYBWS01P19E05 | male | 17 | PTB & EPTB | | 4.11 | 136 | 11 | 18 |
| ZBYBWS01P19E07 | male | 21 | PTB & EPTB | | 4.7 | 117 | 14 | 31 |
| ZBYBWS01P19E08 | male | 27 | PTB & EPTB | | 5.01 | 138 | 14 | 14 |
| ZBYBWS01P19E09 | male | 80 | PTB | | NA | 115 | 22 | 13 |
| ZBYBWS01P19F01 | male | 30 | PTB | | 8.23 | 113 | 8 | 16 |
| ZBYBWS01P19F08 | male | 17 | PTB | | 11.02 | 129 | 7 | 10 |
| ZBYBWS01P19F10 | male | 55 | PTB | | 4.32 | 134 | 8 | 12 |
| ZBYBWS01P19F12 | male | 67 | PTB | | 3.94 | 105 | 11 | 28 |
| ZBYBWS01P19G01 | male | 64 | PTB & EPTB | | 8.21 | 100 | 20 | 25 |
| ZBYBWS01P19G02 | male | 59 | PTB | | 2.81 | 147 | 11 | 15 |
| ZBYBWS01P19G04 | male | 61 | PTB | | NA | 142 | NA | NA |
| ZBYBWS01P19G06 | female | 65 | PTB & EPTB | | 4.5 | 125 | 11 | 15 |
| ZBYBWS01P19G07 | male | 54 | PTB | | 6.41 | 147 | 21 | 33 |
| ZBYBWS01P19G08 | male | 24 | PTB | | NA | 154 | NA | NA |
| ZBYBWS01P19G09 | male | 73 | EPTB | | NA | 142 | NA | NA |
| ZBYBWS01P19G10 | male | 50 | PTB & EPTB | | 5.01 | 117 | 9 | 12 |
| ZBYBWS01P19G11 | male | 71 | PTB | | 3.77 | 121 | 19 | 26 |
| ZBYBWS01P19G12 | male | 27 | PTB | | 6.46 | 151 | 20 | 19 |
| ZBYBWS01P19H01 | male | 39 | PTB | | 3.49 | 106 | 36 | 39 |
| ZBYBWS01P19H03 | female | 33 | PTB & EPTB | | 6.36 | 128 | 23 | 34 |
| ZBYBWS01P19H04 | male | 43 | PTB & EPTB | | 3.7 | 143 | 88 | 60 |
| ZBYBWS01P19H08 | male | 27 | PTB | | 2.38 | 149 | 7 | 16 |
| ZBYBWS01P19H10 | male | 66 | PTB | | 4.81 | 133 | 17 | 21 |
| ZBYBWS01P20A02 | female | 34 | PTB & EPTB | | 3.02 | 125 | 24 | 21 |
| ZBYBWS01P20A03 | male | 34 | PTB | | 5.66 | 116 | 11 | 18 |
| ZBYBWS01P20A04 | male | 79 | PTB | | 4.07 | 112 | 59 | 86 |
| ZBYBWS01P20A06 | male | 17 | PTB & EPTB | | 4.77 | 159 | 20 | 43 |
| ZBYBWS01P20A10 | male | 69 | PTB | | NA | 138 | 12 | 27 |
| ZBYBWS01P20A11 | male | 60 | PTB | | NA | 146 | NA | NA |
| ZBYBWS01P20B04 | male | 55 | PTB | | 4.18 | 147 | 10 | 19 |
| ZBYBWS01P20B06 | female | 31 | PTB & EPTB | | 5.38 | 132 | 12 | 17 |
| ZBYBWS01P20B09 | male | 52 | PTB & EPTB | | 2.26 | 115 | 9 | 22 |
| ZBYBWS01P20C03 | male | 48 | PTB | | 8.91 | 128 | 15 | 21 |
| ZBYBWS01P20C05 | female | 38 | PTB | | 4.74 | 117 | 38 | 107 |
| ZBYBWS01P20C07 | female | 51 | PTB | | NA | 120 | NA | NA |
| ZBYBWS01P20C09 | male | 49 | PTB | | 3.07 | 145 | 41 | 32 |
| ZBYBWS01P20C10 | male | 25 | PTB & EPTB | | 4.12 | 136 | 8 | 17 |
| ZBYBWS01P20C11 | male | 51 | PTB | | 7.7 | 128 | 24 | 30 |
| ZBYBWS01P20D01 | female | 26 | PTB | | 4.13 | 128 | 10 | 12 |
| ZBYBWS01P20D02 | male | 45 | PTB | | 5.8 | 152 | 23 | 18 |
| ZBYBWS01P20D03 | female | 27 | PTB & EPTB | | 4.23 | 129 | 10 | 19 |
| ZBYBWS01P20D04 | female | 54 | PTB | | 2.25 | 102 | 36 | 34 |
| ZBYBWS01P20D06 | female | 27 | PTB & EPTB | | 6.62 | 93 | 42 | 19 |
| ZBYBWS01P20D08 | male | 51 | PTB & EPTB | | 10.92 | 129 | 17 | 22 |
| ZBYBWS01P20D09 | female | 31 | PTB | | 3.62 | 99 | 25 | 25 |
| ZBYBWS01P20D12 | male | 20 | PTB | | 6.11 | 125 | 23 | 26 |
| ZBYBWS01P20E01 | female | 42 | PTB | | 3.99 | 136 | 21 | 26 |
| ZBYBWS01P20E02 | male | 64 | PTB | | 6.51 | 113 | 23 | 16 |
| ZBYBWS01P20E03 | female | 32 | PTB | | 6.51 | 114 | 34 | 32 |
| ZBYBWS01P20E04 | female | 40 | PTB | | 5.67 | 120 | 29 | 44 |
| ZBYBWS01P20E05 | female | 25 | PTB & EPTB | | 5.14 | 116 | 10 | 21 |
| ZBYBWS01P20E06 | male | 43 | PTB & EPTB | | 10.07 | 127 | 22 | 30 |
| ZBYBWS01P20E08 | male | 39 | PTB | | 3.83 | 115 | 12 | 14 |
| ZBYBWS01P20E09 | male | 63 | PTB | | 4.52 | 120 | 9 | 36 |
| ZBYBWS01P20E10 | female | 32 | PTB | | 4.64 | 149 | 21 | 30 |
| ZBYBWS01P20E12 | male | 35 | PTB | | NA | 139 | NA | NA |
| ZBYBWS01P20F01 | male | 84 | PTB | | 6.77 | 104 | 15 | 25 |
| ZBYBWS01P20F03 | male | 48 | PTB | | 6.2 | 139 | 52 | 30 |
| ZBYBWS01P20F04 | female | 40 | PTB | | 4.17 | 138 | 15 | 18 |
| ZBYBWS01P20F05 | male | 38 | PTB | | 11.87 | 152 | 23 | 20 |
| ZBYBWS01P20F06 | male | 36 | PTB | | 2.92 | 161 | 22 | 29 |
| ZBYBWS01P20F07 | female | 21 | PTB & EPTB | | 9.85 | 94 | 12 | 19 |
| ZBYBWS01P20F08 | male | 62 | PTB & EPTB | | 1.49 | 100 | 16 | 24 |
| ZBYBWS01P20F09 | male | 54 | PTB | | 4.61 | 169 | 11 | 21 |
| ZBYBWS01P20F10 | female | 58 | PTB | | 2.69 | 130 | 18 | 17 |
| ZBYBWS01P20F11 | female | 66 | PTB | | 4.4 | 101 | 19 | 17 |
| ZBYBWS01P20G01 | male | 45 | PTB | | 2.4 | 147 | 17 | 23 |
| ZBYBWS01P20G04 | female | 35 | PTB | | 1.98 | 143 | 15 | 16 |
| ZBYBWS01P20G06 | female | 27 | PTB | | 8.14 | 134 | 40 | 40 |
| ZBYBWS01P20G07 | female | 72 | PTB | | 3.63 | 135 | 16 | 26 |
| ZBYBWS01P20G09 | female | 66 | PTB | | 6.62 | 90 | 7 | 21 |
| ZBYBWS01P20G11 | female | 31 | PTB | | 2.9 | 104 | 47 | 45 |
| ZBYBWS01P20G12 | male | 50 | PTB | | 4.22 | 128 | 9 | 15 |
| ZBYBWS01P20H02 | male | 59 | PTB & EPTB | | 5.71 | 118 | 25 | 29 |
| ZBYBWS01P20H03 | male | 27 | PTB | | NA | 124 | NA | NA |
| ZBYBWS01P20H04 | male | 69 | PTB | | 4.66 | 78 | 13 | 24 |
| ZBYBWS01P20H06 | female | 52 | PTB | | 3.13 | 120 | 26 | 30 |
| ZBYBWS01P20H07 | female | 30 | PTB | | 5.31 | 140 | 15 | 20 |
| ZBYBWS01P20H08 | female | 19 | PTB | | 2.24 | 146 | 9 | 15 |
| ZBYBWS01P20H09 | female | 17 | PTB | | 3.41 | 141 | 6 | 17 |
| ZBYBWS01P20H10 | male | 28 | PTB | | 2.37 | 149 | 12 | 21 |
| ZBYBWS01P23A02 | female | 27 | PTB | | 3.62 | 138 | 10 | 21 |
| ZBYBWS01P23A03 | male | 24 | PTB | | 3.65 | 164 | 11 | 28 |
| ZBYBWS01P23A04 | male | 69 | PTB | | 8 | 137 | 13 | 31 |
| ZBYBWS01P23A05 | female | 30 | PTB | | 3.51 | 134 | 65 | 48 |
| ZBYBWS01P23A06 | female | 54 | PTB | | 2.38 | 131 | 14 | 25 |
| ZBYBWS01P23A07 | female | 20 | PTB | | 2.39 | 119 | 12 | 16 |
| ZBYBWS01P23A09 | male | 21 | PTB | | 11.85 | 138 | 15 | 20 |
| ZBYBWS01P23A10 | male | 77 | PTB | | 4.37 | 115 | 16 | 28 |
| ZBYBWS01P23A11 | male | 58 | PTB | | 8.18 | 136 | 28 | 25 |
| ZBYBWS01P23A12 | male | 17 | PTB & EPTB | | 10.25 | 107 | 27 | 17 |
| ZBYBWS01P23B01 | male | 40 | PTB & EPTB | | 4.94 | 155 | 15 | 19 |
| ZBYBWS01P23B02 | male | 59 | PTB & EPTB | | NA | 114 | NA | NA |
| ZBYBWS01P23B03 | male | 81 | PTB | | NA | 136 | NA | NA |
| ZBYBWS01P23B04 | male | 84 | PTB | | NA | 92 | NA | NA |
| ZBYBWS01P23B12 | male | 73 | PTB | | 8.08 | 93 | 46 | 34 |
| ZBYBWS01P23C01 | female | 23 | PTB & EPTB | | NA | 97 | NA | NA |
| ZBYBWS01P23C02 | male | 17 | PTB & EPTB | | NA | 111 | NA | NA |
| ZBYBWS01P23C03 | male | 58 | EPTB | | NA | 153 | NA | NA |
| ZBYBWS01P23C07 | male | 41 | PTB | | NA | 161 | NA | NA |
| ZBYBWS01P23C08 | female | 47 | PTB | | NA | 128 | 9 | 15 |
| ZBYBWS01P23C09 | male | 41 | PTB | | 12.93 | 115 | 77 | 128 |
| ZBYBWS01P23C10 | male | 67 | PTB | | 12.93 | 93 | 4 | 9 |
| ZBYBWS01P23C11 | female | 62 | PTB | | NA | 117 | NA | NA |
| ZBYBWS01P23D02 | male | 71 | PTB | | NA | 111 | NA | NA |
| ZBYBWS01P23D05 | male | 17 | PTB & EPTB | | NA | 79 | NA | NA |
| ZBYBWS01P23D11 | female | 35 | PTB | | 2.71 | 128 | 11 | 17 |
| ZBYBWS01P23D12 | female | 30 | PTB | | 3.6 | 125 | 13 | 14 |
| ZBYBWS01P23E03 | male | 58 | PTB | | 3.14 | 143 | 17 | 22 |
| ZBYBWS01P23E04 | male | 47 | PTB | | 2.47 | 162 | 33 | 26 |
| ZBYBWS01P23E05 | male | 18 | PTB & EPTB | | 6.98 | 153 | 11 | 17 |
| ZBYBWS01P23E06 | female | 32 | PTB | | 6.67 | 105 | NA | NA |
| ZBYBWS01P23E07 | female | 28 | PTB & EPTB | | 6.08 | 124 | 4 | 12 |
| ZBYBWS01P23E08 | male | 29 | PTB | | 5.51 | 140 | 15 | 30 |
| ZBYBWS01P23E09 | male | 49 | PTB | | 3.65 | 109 | 20 | 22 |
| ZBYBWS01P23E11 | male | 61 | PTB | | 12 | 124 | 59 | 32 |
| ZBYBWS01P23E12 | male | 50 | PTB | | NA | 177 | NA | NA |
| ZBYBWS01P23F02 | female | 44 | PTB & EPTB | | 3.34 | 119 | 126 | 94 |
| ZBYBWS01P23F03 | male | 45 | PTB | | 3.53 | 161 | 27 | 24 |
| ZBYBWS01P23F04 | female | 31 | PTB & EPTB | | 2.54 | 152 | 17 | 18 |
| ZBYBWS01P23F06 | male | 26 | PTB | | 4.52 | 108 | 24 | 19 |
| ZBYBWS01P23F08 | female | 15 | PTB | | 2.01 | NA | 8 | 10 |
| ZBYBWS01P23F09 | male | 16 | PTB | | 5.2 | 135 | 19 | 27 |
| ZBYBWS01P23F10 | female | 21 | PTB | | 5.98 | 132 | 6 | 15 |
| ZBYBWS01P23F12 | male | 37 | PTB | | 9.57 | 121 | 48 | 50 |
| ZBYBWS01P23G01 | male | 54 | PTB | | 3.75 | 123 | 25 | 29 |
| ZBYBWS01P23G03 | male | 31 | PTB | | NA | 168 | NA | NA |
| ZBYBWS01P23G04 | female | 26 | PTB | | 10.06 | 96 | 27 | 58 |
| ZBYBWS01P23G06 | female | 28 | PTB | | 4.31 | 136 | 15 | 24 |
| ZBYBWS01P23G07 | male | 21 | PTB | | 5.11 | 172 | 34 | 28 |
| ZBYBWS01P23G08 | male | 19 | PTB | | 9.11 | 127 | 24 | 19 |
| ZBYBWS01P23G09 | female | 30 | PTB | | 3.45 | 137 | 19 | 24 |
| ZBYBWS01P23G11 | male | 61 | PTB | | 4.59 | 98 | 74 | 71 |
| ZBYBWS01P23G12 | female | 19 | PTB & EPTB | | NA | 132 | NA | NA |
| ZBYBWS01P23H01 | male | 52 | PTB | | 8.32 | NA | 19 | 23 |
| ZBYBWS01P23H02 | male | 64 | PTB | | 14.13 | 114 | 6 | 13 |
| ZBYBWS01P23H03 | male | 54 | PTB & EPTB | | 1.76 | 152 | 12 | 10 |
| ZBYBWS01P23H04 | female | 46 | PTB | | 5.32 | 138 | 12 | 23 |
| ZBYBWS01P23H06 | female | 27 | PTB | | 2.23 | NA | 8 | 12 |
| ZBYBWS01P23H08 | female | 23 | PTB | | NA | 119 | NA | NA |
| ZBYBWS01P23H09 | male | 22 | PTB | | NA | 117 | NA | NA |
| ZBYBWS01P23H10 | female | 55 | PTB | | NA | 137 | NA | NA |
| ZBYBWS01P23H12 | male | 44 | PTB | | 7.19 | 146 | 15 | 23 |
| ZBYBWS01P24A02 | female | 30 | PTB | | NA | 123 | NA | NA |
| ZBYBWS01P24A03 | male | 25 | PTB | | NA | NA | NA | NA |
| ZBYBWS01P24A04 | female | 61 | PTB | | 4.66 | 125 | 19 | 19 |
| ZBYBWS01P24A05 | male | 52 | PTB | | NA | 199 | NA | NA |
| ZBYBWS01P24A06 | male | 62 | PTB | | NA | 144 | NA | NA |
| ZBYBWS01P24A07 | female | 34 | PTB | | NA | 136 | NA | NA |
| ZBYBWS01P24A08 | female | 33 | PTB | | 7.21 | 150 | 11 | 20 |
| ZBYBWS01P24A09 | female | 27 | PTB | | 10.01 | 135 | 17 | 23 |
| ZBYBWS01P24A10 | male | 53 | PTB | | NA | 123 | NA | NA |
| ZBYBWS01P24A11 | female | 22 | PTB | | NA | 119 | NA | NA |
| ZBYBWS01P24A12 | female | 74 | PTB | | 2.57 | 118 | 12 | 23 |
| ZBYBWS01P24B01 | male | 39 | PTB | | NA | 116 | NA | NA |
| ZBYBWS01P24B02 | male | 23 | PTB | | 4.5 | 152 | 41 | 58 |
| ZBYBWS01P24B03 | male | 84 | PTB | | 5.21 | 91 | 0 | 0 |
| ZBYBWS01P24B04 | female | 50 | PTB | | 11.52 | 116 | 5 | 10 |
| ZBYBWS01P24B05 | male | 32 | EPTB | | 2.18 | 172 | 14 | 25 |
| ZBYBWS01P24B06 | male | 18 | PTB | | 7.71 | 98 | 13 | 11 |
| ZBYBWS01P24B07 | male | 29 | PTB | | NA | 159 | NA | NA |
| ZBYBWS01P24B08 | female | 20 | PTB | | 3.36 | 142 | 25 | 21 |
| ZBYBWS01P24B09 | female | 41 | EPTB | | NA | 128 | NA | NA |
| ZBYBWS01P24B10 | male | 62 | PTB | | NA | 126 | NA | NA |
| ZBYBWS01P24B11 | female | 54 | PTB | | NA | 143 | NA | NA |
| ZBYBWS01P24B12 | female | 33 | PTB | | NA | 140 | NA | NA |
| ZBYBWS01P24C01 | male | 32 | PTB | | 8.94 | 142 | 18 | 25 |
| ZBYBWS01P24C02 | male | 28 | EPTB | | NA | NA | NA | NA |
| ZBYBWS01P24C03 | male | 52 | PTB | | 8.1 | 64 | 15 | 19 |
| ZBYBWS01P24C06 | male | 59 | PTB | | 4.01 | 123 | 9 | 8 |
| ZBYBWS01P24C07 | female | 29 | PTB | | NA | 137 | NA | NA |
| ZBYBWS01P24C08 | male | 71 | PTB | | 8.94 | 105 | 8 | 16 |
| ZBYBWS01P24C09 | male | 27 | PTB & EPTB | | 5.43 | 127 | 21 | 24 |
| ZBYBWS01P24D01 | female | 49 | PTB | | 2.11 | 129 | 11 | 17 |
| ZBYBWS01P24D02 | female | 24 | PTB | | 8.96 | 124 | 13 | 13 |
| ZBYBWS01P24D04 | female | 31 | PTB | | NA | 96 | NA | NA |
| ZBYBWS01P24D05 | male | 49 | PTB | | NA | 144 | NA | NA |
| ZBYBWS01P24D06 | male | 20 | PTB | | 14.79 | 113 | 16 | 15 |
| ZBYBWS01P24D07 | female | 59 | PTB | | 1.32 | 120 | 18 | 18 |
| ZBYBWS01P24D08 | male | 74 | PTB | | 3.47 | 143 | 20 | 31 |
| ZBYBWS01P24D09 | male | 39 | PTB | | 6.17 | 147 | 15 | 22 |
| ZBYBWS01P24D10 | male | 46 | PTB & EPTB | | 4.62 | 131 | 50 | 68 |
| ZBYBWS01P24D11 | male | 26 | PTB | | NA | 161 | NA | NA |
| ZBYBWS01P24D12 | male | 48 | PTB | | 4.72 | 99 | 9 | 12 |
| ZBYBWS01P24E01 | male | 37 | PTB | | 4.42 | NA | 34 | 24 |
| ZBYBWS01P24E02 | female | 37 | PTB | | NA | 115 | NA | NA |
| ZBYBWS01P24E03 | male | 28 | PTB | | 3.08 | 135 | 28 | 24 |
| ZBYBWS01P24E05 | male | 49 | PTB | | 5.05 | 124 | 15 | 22 |
| ZBYBWS01P24E07 | male | 61 | EPTB | | NA | NA | NA | NA |
| ZBYBWS01P24E08 | female | 45 | PTB & EPTB | | 3.32 | 119 | 19 | 15 |
| ZBYBWS01P24E10 | male | 92 | PTB | | 2.01 | 130 | 21 | 32 |
| ZBYBWS01P24E12 | female | 75 | PTB | | 2.63 | 114 | 10 | 17 |
| ZBYBWS01P24F01 | female | 20 | PTB & EPTB | | 5.21 | 150 | 14 | 20 |
| ZBYBWS01P24F02 | female | 46 | PTB | | NA | 138 | 52 | 55 |
| ZBYBWS01P24F03 | male | 82 | PTB & EPTB | | NA | 85 | 11 | 28 |
| ZBYBWS01P24F04 | female | 26 | PTB | | 2.93 | 135 | 9 | 18 |
| ZBYBWS01P24F05 | male | 54 | PTB & EPTB | | NA | 135 | 63 | 35 |
| ZBYBWS01P24F06 | female | 25 | PTB | | 5.55 | 110 | 11 | 22 |
| ZBYBWS01P24F07 | male | 26 | EPTB | | 7 | 137 | 6 | 11 |
| ZBYBWS01P24F08 | female | 68 | PTB | | 8.6 | 97 | 5 | 13 |
| ZBYBWS01P24F09 | male | 32 | PTB | | NA | 168 | NA | NA |
| ZBYBWS01P24F11 | female | 31 | PTB | | NA | 112 | 44 | 21 |
| ZBYBWS01P24F12 | male | 64 | PTB & EPTB | | NA | 136 | 47 | 22 |
| ZBYBWS01P24G03 | female | 17 | EPTB | | NA | 94 | 55 | 46 |
| ZBYBWS01P24G07 | male | 20 | PTB | | NA | NA | 46 | 27 |
| ZBYBWS01P24G08 | female | 39 | PTB & EPTB | | NA | 113 | 35 | 61 |
| ZBYBWS01P24G10 | female | 36 | PTB | | NA | 153 | 10 | 23 |
| ZBYBWS01P24G11 | male | 55 | PTB | | NA | 161 | 30 | 29 |
| ZBYBWS01P24H09 | female | 32 | PTB | | NA | 78 | NA | NA |
| ZBYBWS01P24H12 | male | 41 | PTB & EPTB | | NA | 138 | 14 | 11 |
| ZBYBWS01P27A03 | male | 19 | PTB | | NA | 109 | 5 | 18 |
| ZBYBWS01P27A04 | male | 36 | EPTB | | NA | NA | NA | NA |
| ZBYBWS01P27A05 | female | 23 | PTB | | NA | 138 | 16 | 20 |
| ZBYBWS01P27A08 | female | 35 | EPTB | | NA | 137 | 14 | 17 |
| ZBYBWS01P27A09 | female | 25 | PTB & EPTB | | NA | 85 | 7 | 14 |
| ZBYBWS01P27B02 | male | 63 | EPTB | | NA | 115 | 32 | 20 |
| ZBYBWS01P27B03 | female | 80 | EPTB | | NA | 89 | 24 | 39 |
| ZBYBWS01P27B08 | female | 43 | PTB & EPTB | | NA | 117 | 37 | 32 |
| ZBYBWS01P27B11 | male | 28 | PTB | | NA | 141 | 22 | 22 |
| ZBYBWS01P27B12 | male | 74 | EPTB | | NA | 110 | 18 | 22 |
| ZBYBWS01P27C03 | female | 37 | PTB & EPTB | | NA | 110 | 18 | 44 |
| ZBYBWS01P27C04 | male | 38 | PTB | | NA | 91 | 8 | 12 |
| ZBYBWS01P27C05 | male | 23 | EPTB | | NA | 138 | 27 | 25 |
| ZBYBWS01P27C08 | male | 83 | EPTB | | NA | 155 | 12 | 17 |
| ZBYBWS01P27C12 | male | 25 | PTB & EPTB | | NA | 111 | 28 | 40 |
| ZBYBWS01P27D01 | male | 20 | PTB | | NA | 138 | 13 | 16 |
| ZBYBWS01P27D07 | female | 67 | PTB & EPTB | | NA | 102 | 29 | 34 |
| ZBYBWS01P27D08 | female | 50 | PTB | | NA | 140 | 15 | 24 |
| ZBYBWS01P27E08 | male | 18 | PTB | | NA | 108 | 3 | 15 |
| ZBYBWS01P27E12 | male | 62 | PTB | | NA | 134 | 8 | 19 |
| ZBYBWS01P27F02 | male | 40 | PTB | | NA | 129 | 38 | 37 |
| ZBYBWS01P27F06 | female | 78 | PTB | | NA | 124 | 12 | 19 |
| ZBYBWS01P27F10 | male | 37 | PTB | | NA | 112 | 10 | 11 |
| ZBYBWS01P27G03 | male | 62 | PTB | | NA | 121 | NA | NA |
| ZBYBWS01P27G04 | female | 29 | EPTB | | NA | 108 | 6 | 14 |
| ZBYBWS01P27G05 | male | 50 | PTB | | NA | 138 | 32 | 19 |
| ZBYBWS01P27G06 | male | 18 | PTB | | NA | 153 | NA | NA |
| ZBYBWS01P27H06 | male | 43 | PTB & EPTB | | NA | 123 | 8 | 10 |
| ZBYBWS01P27H09 | male | 49 | EPTB | | NA | NA | 16 | 12 |
| ZBYBWS01P28A02 | male | 22 | PTB & EPTB | | NA | NA | NA | NA |
| ZBYBWS01P28A08 | male | 23 | PTB & EPTB | | NA | 127 | 35 | 26 |
| ZBYBWS01P28B01 | male | 60 | PTB | | NA | 141 | 15 | 30 |
| ZBYBWS02P02A03 | female | 71 | PTB | | 1.34 | 127 | 17 | 34 |
| ZBYBWS02P02A04 | female | 75 | PTB | | 2.99 | 111 | 53 | 173 |
| ZBYBWS02P02A06 | female | 62 | PTB | | NA | 137 | NA | NA |
| ZBYBWS02P02A08 | female | 41 | PTB | | 3.92 | NA | 7 | 12 |
| ZBYBWS02P02A09 | male | 36 | PTB | | 4.98 | 118 | 59 | 46 |
| ZBYBWS02P02A10 | male | 54 | PTB | | 3.04 | NA | 13 | 18 |
| ZBYBWS02P02A11 | female | 59 | PTB & EPTB | | 3.53 | 135 | 23 | 35 |
| ZBYBWS02P02B01 | female | 26 | PTB | | NA | 115 | NA | NA |
| ZBYBWS02P02B02 | female | 66 | PTB & EPTB | | 3.23 | 108 | 9 | 16 |
| ZBYBWS02P02B04 | male | 42 | PTB | | NA | 146 | NA | NA |
| ZBYBWS02P02B05 | female | 20 | PTB | | 1.93 | 99 | 7 | 11 |
| ZBYBWS02P02B07 | male | 18 | PTB | | NA | 163 | NA | NA |
| ZBYBWS02P02B08 | male | 16 | PTB | | NA | 151 | NA | NA |
| ZBYBWS02P02B12 | female | 68 | PTB & EPTB | | 16.25 | 95 | 7 | 50 |
| ZBYBWS02P02C01 | male | 42 | PTB | | NA | 188 | NA | NA |
| ZBYBWS02P02C02 | female | 72 | PTB | | NA | 106 | NA | NA |
| ZBYBWS02P02C04 | male | 40 | PTB | | 4.42 | 125 | 34 | 30 |
| ZBYBWS02P02C05 | male | 16 | PTB | | NA | 146 | NA | NA |
| ZBYBWS02P02C06 | male | 75 | PTB | | 6.98 | 122 | 19 | 24 |
| ZBYBWS02P02C07 | female | 51 | PTB | | 4.5 | 135 | 61 | 53 |
| ZBYBWS02P02C08 | female | 54 | PTB | | NA | 133 | NA | NA |
| ZBYBWS02P02C09 | male | 53 | PTB | | 6.31 | 146 | 14 | 20 |
| ZBYBWS02P02C10 | male | 61 | PTB | | 13.91 | 125 | 23 | 22 |
| ZBYBWS02P02C11 | male | 40 | PTB | | NA | 156 | NA | NA |
| ZBYBWS02P02D01 | female | 25 | PTB | | 2.11 | 149 | 19 | 26 |
| ZBYBWS02P02D03 | male | 51 | PTB | | 6.05 | 90 | 49 | 47 |
| ZBYBWS02P02D04 | female | 31 | PTB | | NA | 106 | NA | NA |
| ZBYBWS02P02D05 | male | 66 | PTB | | 2.3 | 111 | 9 | 13 |
| ZBYBWS02P02D06 | female | 45 | PTB | | NA | 115 | NA | NA |
| ZBYBWS02P02D07 | female | 67 | PTB | | NA | 117 | NA | NA |
| ZBYBWS02P02D08 | male | 48 | PTB | | 6.01 | 125 | 40 | 27 |
| ZBYBWS02P02D09 | male | 35 | PTB | | 5.7 | 143 | 13 | 27 |
| ZBYBWS02P02D11 | female | 39 | PTB | | NA | 115 | NA | NA |
| ZBYBWS02P02D12 | female | 33 | PTB | | NA | 143 | NA | NA |
| ZBYBWS02P02E01 | female | 33 | PTB | | 10.03 | 115 | 15 | 26 |
| ZBYBWS02P02E02 | female | 30 | PTB | | 3.63 | 147 | 32 | 40 |
| ZBYBWS02P02E03 | male | 73 | PTB | | 3.1 | 97 | 28 | 30 |
| ZBYBWS02P02E04 | male | 36 | PTB | | 4.19 | 150 | 17 | 24 |
| ZBYBWS02P02E05 | male | 52 | PTB | | 3.41 | 152 | 22 | 23 |
| ZBYBWS02P02E06 | male | 27 | PTB & EPTB | | 11.33 | 137 | 18 | 20 |
| ZBYBWS02P02E07 | female | 37 | PTB | | 7.96 | 115 | 5 | 13 |
| ZBYBWS02P02E08 | female | 55 | PTB | | 3.7 | 124 | 7 | 14 |
| ZBYBWS02P02E11 | male | 66 | PTB | | 2.9 | 140 | 62 | 38 |
| ZBYBWS02P02E12 | female | 44 | PTB | | 3.81 | 120 | 8 | 21 |
| ZBYBWS02P02F01 | male | 73 | PTB | | 3.22 | 144 | 19 | 34 |
| ZBYBWS02P02F02 | female | 63 | EPTB | | 3.39 | 144 | 15 | 29 |
| ZBYBWS02P02F03 | male | 28 | PTB | | NA | 156 | NA | NA |
| ZBYBWS02P02F04 | female | 49 | PTB | | 3.95 | 133 | 16 | 23 |
| ZBYBWS02P02F05 | male | 74 | PTB | | 5.09 | 105 | 20 | 54 |
| ZBYBWS02P02F06 | female | 31 | PTB | | 2.86 | 125 | 19 | 17 |
| ZBYBWS02P02F08 | male | 25 | PTB | | NA | 154 | NA | NA |
| ZBYBWS01P16H05 | male | 50 | PTB & EPTB | | 3.74 | 108 | 6 | 12 |
| ZBYBWS02P02F12 | male | 42 | PTB | | 3.87 | 108 | 40 | 34 |
| ZBYBWS02P02G01 | female | 60 | PTB | | 13.82 | 131 | 12 | 18 |
| ZBYBWS02P02G02 | male | 20 | PTB | | NA | 154 | NA | NA |
| ZBYBWS02P02G03 | male | 54 | PTB | | 4.4 | 160 | 12 | 21 |
| ZBYBWS02P02G04 | female | 29 | PTB | | 1.92 | 139 | 18 | 23 |
| ZBYBWS02P02G07 | female | 46 | PTB | | NA | 122 | NA | NA |
| ZBYBWS02P02G09 | male | 25 | PTB | | NA | 155 | NA | NA |
| ZBYBWS02P02G11 | female | 30 | PTB & EPTB | | NA | 129 | NA | NA |
| ZBYBWS02P02G12 | female | 46 | PTB | | 13.3 | 91 | 10 | 14 |
| ZBYBWS02P02H01 | female | 30 | PTB & EPTB | | NA | 127 | NA | NA |
| ZBYBWS02P02H02 | male | 22 | PTB & EPTB | | 10.39 | 117 | 101 | 81 |
| ZBYBWS02P02H03 | male | 30 | PTB | | NA | NA | NA | NA |
| ZBYBWS02P02H04 | male | 34 | PTB | | 2.46 | 142 | 18 | 19 |
| ZBYBWS02P02H05 | male | 28 | PTB | | 3.31 | 132 | 13 | 21 |
| ZBYBWS02P02H06 | female | 35 | PTB | | 3.38 | 110 | 16 | 24 |
| ZBYBWS02P02H07 | male | 47 | PTB | | 4.45 | 150 | 10 | 20 |
| ZBYBWS02P02H08 | male | 61 | PTB | | NA | 117 | NA | NA |
| ZBYBWS02P02H09 | male | 60 | PTB | | NA | NA | NA | NA |
| ZBYBWS02P02H10 | male | 26 | PTB & EPTB | | 5.94 | 124 | 27 | 29 |
| ZBYBWS02P05A03 | female | 54 | EPTB | | NA | NA | NA | NA |
| ZBYBWS02P05A04 | female | 22 | PTB | | 2.45 | 124 | 14 | 19 |
| ZBYBWS02P05A05 | male | 42 | PTB & EPTB | | 3.65 | 97 | 28 | 54 |
| ZBYBWS02P05A06 | female | 48 | PTB | | 2.73 | 136 | 25 | 39 |
| ZBYBWS02P05A08 | female | 27 | PTB | | NA | 134 | NA | NA |
| ZBYBWS02P05A09 | female | 58 | PTB | | 2.47 | 98 | 19 | 23 |
| ZBYBWS02P05A10 | male | 58 | PTB | | NA | 145 | NA | NA |
| ZBYBWS02P05B01 | male | 65 | PTB | | 1.22 | 71 | 8 | 15 |
| ZBYBWS02P05B02 | male | 19 | PTB | | NA | 177 | NA | NA |
| ZBYBWS02P05B04 | male | 64 | PTB | | 4.6 | 154 | 76 | 92 |
| ZBYBWS02P05B07 | male | 44 | PTB | | 9.05 | 122 | 63 | 182 |
| ZBYBWS02P05B10 | female | 35 | PTB | | 3.34 | 119 | 16 | 30 |
| ZBYBWS02P05B11 | male | 44 | PTB | | 9.14 | 126 | 21 | 28 |
| ZBYBWS02P05B12 | male | 25 | PTB | | 17.71 | 108 | 8 | 28 |
| ZBYBWS02P05C01 | male | 41 | PTB | | 5.43 | 118 | 26 | 15 |
| ZBYBWS02P05C03 | female | 79 | PTB | | 2.16 | 107 | 8 | 19 |
| ZBYBWS02P05C04 | male | 74 | PTB | | NA | 100 | NA | NA |
| ZBYBWS02P05C05 | male | 45 | PTB | | NA | 153 | NA | NA |
| ZBYBWS02P05C06 | female | 45 | PTB & EPTB | | 4.65 | 115 | 24 | 37 |
| ZBYBWS02P05C07 | male | 50 | PTB | | 2.96 | 146 | 10 | 15 |
| ZBYBWS02P05C08 | male | 18 | PTB | | NA | 149 | NA | NA |
| ZBYBWS02P05C09 | male | 19 | PTB | | NA | 131 | NA | NA |
| ZBYBWS02P05C10 | male | 17 | PTB | | NA | 151 | NA | NA |
| ZBYBWS02P05C11 | male | 19 | PTB & EPTB | | 12.42 | 122 | 33 | 46 |
| ZBYBWS02P05C12 | male | 33 | PTB | | 6.73 | 146 | 9 | 16 |
| ZBYBWS02P05D01 | female | 37 | PTB | | NA | 128 | NA | NA |
| ZBYBWS02P05D02 | male | 50 | PTB | | 3.79 | 125 | 20 | 24 |
| ZBYBWS02P05D03 | male | 20 | PTB | | NA | 170 | NA | NA |
| ZBYBWS02P05D04 | male | 16 | PTB & EPTB | | 3.34 | 107 | 10 | 37 |
| ZBYBWS02P05D05 | male | 30 | PTB | | NA | 137 | NA | NA |
| ZBYBWS02P05D06 | female | 25 | PTB | | 5.28 | 99 | 20 | 21 |
| ZBYBWS02P05D07 | male | 21 | PTB | | NA | 146 | NA | NA |
| ZBYBWS02P05D08 | female | 28 | PTB | | 3.43 | 112 | 13 | 80 |
| ZBYBWS02P05D09 | female | 44 | PTB | | 2.76 | 110 | 10 | 28 |
| ZBYBWS02P05D10 | male | 19 | PTB | | NA | 155 | NA | NA |
| ZBYBWS02P05D11 | male | 23 | PTB | | NA | 159 | NA | NA |
| ZBYBWS02P05D12 | female | 41 | PTB | | 4.57 | 117 | 20 | 23 |
| ZBYBWS02P05E02 | male | 51 | PTB | | 1.03 | 106 | 39 | 40 |
| ZBYBWS02P05E03 | male | 15 | EPTB | | 3.01 | 126 | 47 | 27 |
| ZBYBWS02P05E05 | female | 19 | PTB | | NA | 131 | NA | NA |
| ZBYBWS02P05E06 | male | 75 | PTB | | NA | 140 | NA | NA |
| ZBYBWS02P05E07 | male | 51 | PTB | | NA | 167 | NA | NA |
| ZBYBWS02P05E08 | male | 50 | PTB | | 5.81 | 121 | 4 | 17 |
| ZBYBWS02P05E09 | male | 21 | PTB & EPTB | | NA | 134 | 12 | 13 |
| ZBYBWS02P05E10 | female | 66 | PTB | | NA | 123 | NA | NA |
| ZBYBWS02P05E11 | male | 42 | PTB | | 4.3 | 157 | 25 | 23 |
| ZBYBWS02P05F01 | female | 45 | PTB | | NA | 128 | NA | NA |
| ZBYBWS02P05F03 | male | 55 | PTB | | NA | NA | NA | NA |
| ZBYBWS02P05F04 | female | 28 | PTB | | 8.92 | 127 | 36 | 44 |
| ZBYBWS02P05F05 | female | 48 | PTB | | NA | 128 | NA | NA |
| ZBYBWS02P05F06 | female | 19 | PTB | | 2.82 | 127 | 34 | 36 |
| ZBYBWS02P05F07 | female | 24 | PTB | | 3.9 | 138 | 9 | 14 |
| ZBYBWS02P05F08 | male | 23 | PTB | | 11.85 | 102 | 1 | 9 |
| ZBYBWS02P05F10 | female | 43 | PTB | | 3.31 | 128 | 12 | 21 |
| ZBYBWS02P05F11 | male | 68 | PTB | | 2.37 | 92 | 19 | 33 |
| ZBYBWS02P05F12 | female | 49 | PTB & EPTB | | 4.06 | 111 | 17 | 24 |
| ZBYBWS02P05G01 | female | 33 | PTB | | NA | 149 | NA | NA |
| ZBYBWS02P05G02 | female | 29 | PTB | | 5.29 | 118 | 20 | 24 |
| ZBYBWS02P05G03 | female | 30 | PTB | | 3.05 | 135 | 21 | 21 |
| ZBYBWS02P05G04 | male | 25 | PTB | | 4.72 | 153 | 46 | 27 |
| ZBYBWS02P05G05 | male | 18 | PTB | | NA | 164 | 26 | 22 |
| ZBYBWS02P05G06 | male | 59 | PTB | | NA | 158 | NA | NA |
| ZBYBWS02P05G07 | female | 48 | PTB | | NA | 130 | NA | NA |
| ZBYBWS02P05G08 | male | 52 | PTB | | NA | 163 | 21 | 20 |
| ZBYBWS02P05G09 | female | 27 | PTB | | NA | 144 | NA | NA |
| ZBYBWS02P05G11 | female | 60 | PTB | | 4.35 | 142 | 9 | 18 |
| ZBYBWS02P05G12 | male | 20 | PTB | | 2.37 | 157 | 25 | 48 |
| ZBYBWS02P05H01 | female | 23 | PTB | | NA | 112 | NA | NA |
| ZBYBWS02P05H03 | male | 27 | PTB | | 3.78 | 141 | 15 | 15 |
| ZBYBWS02P05H05 | male | 41 | PTB | | NA | 156 | NA | NA |
| ZBYBWS02P05H06 | female | 21 | EPTB | | NA | NA | NA | NA |
| ZBYBWS02P05H07 | male | 35 | PTB | | 1.79 | 167 | 48 | 33 |
| ZBYBWS02P05H08 | male | 48 | PTB | | 6.05 | 141 | 10 | 21 |
| ZBYBWS02P05H09 | male | 75 | PTB | | 5.24 | 120 | 16 | 27 |
| ZBYBWS02P05H10 | male | 42 | PTB | | NA | 114 | 12 | 11 |
| ZBYBWS02P05H11 | female | 28 | PTB | | 2.5 | 125 | 10 | 17 |
| ZBYBWS02P05H12 | male | 28 | PTB | | 5.98 | 101 | 40 | 26 |
| ZBYBWS02P11G07 | male | 26 | PTB | | NA | 157 | NA | NA |
| ZBYBWS02P11G08 | male | 47 | PTB & EPTB | | 7.83 | 117 | 22 | 33 |
| ZBYBWS02P11G09 | female | 31 | PTB | | 5.52 | 121 | 9 | 16 |
| ZBYBWS02P11G11 | male | 67 | PTB | | NA | 138 | 11 | 18 |
| ZBYBWS02P11G12 | female | 21 | PTB | | 77.5 | 138 | 13 | 19 |
| ZBYBWS02P11H01 | male | 35 | PTB | | 6.49 | 165 | 10 | 9 |
| ZBYBWS02P11H02 | male | 22 | PTB | | 4.27 | 130 | 20 | 23 |
| ZBYBWS02P11H03 | male | 40 | PTB | | NA | 158 | NA | NA |
| ZBYBWS02P11H04 | male | 24 | PTB | | NA | 155 | 7 | 21 |
| ZBYBWS02P11H06 | female | 24 | PTB | | NA | 150 | NA | NA |
| ZBYBWS02P11H07 | male | 20 | PTB & EPTB | | 5.66 | 140 | 5 | 10 |
| ZBYBWS02P11H08 | male | 17 | PTB | | NA | 165 | NA | NA |
| ZBYBWS02P11H09 | female | 59 | PTB | | 2.15 | 138 | 44 | 32 |
| ZBYBWS02P11H11 | female | 56 | PTB | | 7.78 | 129 | 62 | 47 |
| ZBYBWS02P12A05 | male | 30 | PTB | | 8.17 | 139 | 34 | 24 |
| ZBYBWS02P12A06 | female | 30 | PTB & EPTB | | NA | 105 | 17 | 18 |
| ZBYBWS02P12A08 | male | 40 | PTB | | NA | 138 | NA | NA |
| ZBYBWS02P12A09 | male | 32 | EPTB | | NA | 157 | 15 | 13 |
| ZBYBWS01P20C04 | female | 27 | PTB | | 3.41 | 114 | 20 | 19 |
| ZBYBWS01P16G04 | female | 67 | PTB | | 6.96 | 128 | 10 | 13 |
| ZBYBWS02P12B01 | male | 49 | PTB | | 17 | 139 | 4 | 17 |
| ZBYBWS02P12B02 | male | 44 | PTB | | NA | 119 | NA | NA |
| ZBYBWS01P16G08 | male | 71 | PTB | | 5.27 | 95 | 24 | 29 |
| ZBYBWS01P20A08 | female | 28 | PTB | | NA | 133 | 12 | 17 |
| ZBYBWS02P12B06 | female | 63 | PTB | | 4.41 | 123 | 25 | 19 |
| ZBYBWS02P12B07 | male | 47 | PTB | | NA | 164 | NA | NA |
| Detailed clinical data of healthy controls | | | | | | | | |
| ZBYBWS01P01A02 | female | 30 | health | |  |  |  |  |
| ZBYBWS01P01A03 | female | 55 | health | |  |  |  |  |
| ZBYBWS01P01A04 | male | 33 | health | |  |  |  |  |
| ZBYBWS01P01A05 | male | 42 | health | |  |  |  |  |
| ZBYBWS01P01A06 | male | 53 | health | |  |  |  |  |
| ZBYBWS01P01A07 | female | 25 | health | |  |  |  |  |
| ZBYBWS01P01A08 | male | 44 | health | |  |  |  |  |
| ZBYBWS01P01A09 | male | 24 | health | |  |  |  |  |
| ZBYBWS01P01A10 | male | 45 | health | |  |  |  |  |
| ZBYBWS01P01A11 | male | 38 | health | |  |  |  |  |
| ZBYBWS01P01A12 | male | 31 | health | |  |  |  |  |
| ZBYBWS01P01B01 | male | 44 | health | |  |  |  |  |
| ZBYBWS01P01B02 | male | 33 | health | |  |  |  |  |
| ZBYBWS01P01B03 | female | 37 | health | |  |  |  |  |
| ZBYBWS01P01B04 | female | 33 | health | |  |  |  |  |
| ZBYBWS01P01B08 | male | 54 | health | |  |  |  |  |
| ZBYBWS01P01B09 | female | 30 | health | |  |  |  |  |
| ZBYBWS01P01B10 | female | 36 | health | |  |  |  |  |
| ZBYBWS01P01B11 | female | 38 | health | |  |  |  |  |
| ZBYBWS01P01B12 | male | 48 | health | |  |  |  |  |
| ZBYBWS01P01C01 | male | 48 | health | |  |  |  |  |
| ZBYBWS01P01C02 | male | 30 | health | |  |  |  |  |
| ZBYBWS01P01C04 | male | 43 | health | |  |  |  |  |
| ZBYBWS01P01C05 | male | 47 | health | |  |  |  |  |
| ZBYBWS01P01C06 | male | 53 | health | |  |  |  |  |
| ZBYBWS01P01C07 | male | 42 | health | |  |  |  |  |
| ZBYBWS01P01C08 | male | 48 | health | |  |  |  |  |
| ZBYBWS01P01C09 | female | 43 | health | |  |  |  |  |
| ZBYBWS01P01C10 | male | 21 | health | |  |  |  |  |
| ZBYBWS01P01C11 | female | 25 | health | |  |  |  |  |
| ZBYBWS01P01C12 | female | 33 | health | |  |  |  |  |
| ZBYBWS01P01D01 | male | 42 | health | |  |  |  |  |
| ZBYBWS01P01D02 | male | 42 | health | |  |  |  |  |
| ZBYBWS01P01D03 | female | 36 | health | |  |  |  |  |
| ZBYBWS01P01D04 | male | 30 | health | |  |  |  |  |
| ZBYBWS01P01D05 | female | 34 | health | |  |  |  |  |
| ZBYBWS01P01D06 | male | 50 | health | |  |  |  |  |
| ZBYBWS01P01D07 | male | 35 | health | |  |  |  |  |
| ZBYBWS01P01D08 | female | 45 | health | |  |  |  |  |
| ZBYBWS01P01D09 | female | 42 | health | |  |  |  |  |
| ZBYBWS01P01D10 | male | 40 | health | |  |  |  |  |
| ZBYBWS01P01D11 | male | 32 | health | |  |  |  |  |
| ZBYBWS01P01D12 | male | 30 | health | |  |  |  |  |
| ZBYBWS01P01E01 | female | 38 | health | |  |  |  |  |
| ZBYBWS01P01E02 | female | 34 | health | |  |  |  |  |
| ZBYBWS01P01E03 | male | 35 | health | |  |  |  |  |
| ZBYBWS01P01E04 | female | 47 | health | |  |  |  |  |
| ZBYBWS01P01E05 | female | 21 | health | |  |  |  |  |
| ZBYBWS01P01E06 | male | 39 | health | |  |  |  |  |
| ZBYBWS01P01E07 | male | 36 | health | |  |  |  |  |
| ZBYBWS01P01E08 | male | 59 | health | |  |  |  |  |
| ZBYBWS01P01E09 | female | 29 | health | |  |  |  |  |
| ZBYBWS01P01E10 | male | 30 | health | |  |  |  |  |
| ZBYBWS01P01E11 | male | 21 | health | |  |  |  |  |
| ZBYBWS01P01E12 | male | 24 | health | |  |  |  |  |
| ZBYBWS01P01F01 | female | 30 | health | |  |  |  |  |
| ZBYBWS01P01F02 | male | 39 | health | |  |  |  |  |
| ZBYBWS01P01F03 | male | 42 | health | |  |  |  |  |
| ZBYBWS01P01F04 | male | 48 | health | |  |  |  |  |
| ZBYBWS01P01F05 | male | 51 | health | |  |  |  |  |
| ZBYBWS01P01F06 | female | 27 | health | |  |  |  |  |
| ZBYBWS01P01F07 | male | 24 | health | |  |  |  |  |
| ZBYBWS01P01F08 | male | 35 | health | |  |  |  |  |
| ZBYBWS01P01F09 | female | 27 | health | |  |  |  |  |
| ZBYBWS01P01F10 | male | 49 | health | |  |  |  |  |
| ZBYBWS01P01F11 | female | 31 | health | |  |  |  |  |
| ZBYBWS01P01F12 | female | 26 | health | |  |  |  |  |
| ZBYBWS01P01G01 | male | 37 | health | |  |  |  |  |
| ZBYBWS01P01G02 | male | 45 | health | |  |  |  |  |
| ZBYBWS01P01G03 | male | 41 | health | |  |  |  |  |
| ZBYBWS01P01G04 | male | 34 | health | |  |  |  |  |
| ZBYBWS01P01G05 | female | 47 | health | |  |  |  |  |
| ZBYBWS01P01G06 | male | 61 | health | |  |  |  |  |
| ZBYBWS01P01G07 | male | 36 | health | |  |  |  |  |
| ZBYBWS01P01G08 | female | 39 | health | |  |  |  |  |
| ZBYBWS01P01G09 | male | 21 | health | |  |  |  |  |
| ZBYBWS01P01G10 | male | 46 | health | |  |  |  |  |
| ZBYBWS01P01G11 | male | 48 | health | |  |  |  |  |
| ZBYBWS01P01G12 | male | 34 | health | |  |  |  |  |
| ZBYBWS01P01H01 | male | 38 | health | |  |  |  |  |
| ZBYBWS01P01H02 | male | 34 | health | |  |  |  |  |
| ZBYBWS01P01H03 | male | 29 | health | |  |  |  |  |
| ZBYBWS01P01H04 | male | 24 | health | |  |  |  |  |
| ZBYBWS01P01H05 | female | 30 | health | |  |  |  |  |
| ZBYBWS01P01H06 | male | 35 | health | |  |  |  |  |
| ZBYBWS01P01H07 | male | 30 | health | |  |  |  |  |
| ZBYBWS01P01H08 | female | 28 | health | |  |  |  |  |
| ZBYBWS01P01H09 | male | 46 | health | |  |  |  |  |
| ZBYBWS01P01H10 | female | 31 | health | |  |  |  |  |
| ZBYBWS01P01H11 | female | 26 | health | |  |  |  |  |
| ZBYBWS01P02A02 | female | 43 | health | |  |  |  |  |
| ZBYBWS01P02A03 | female | 40 | health | |  |  |  |  |
| ZBYBWS01P02A04 | female | 29 | health | |  |  |  |  |
| ZBYBWS01P02A05 | female | 43 | health | |  |  |  |  |
| ZBYBWS01P02A06 | male | 24 | health | |  |  |  |  |
| ZBYBWS01P02A07 | male | 34 | health | |  |  |  |  |
| ZBYBWS01P02A08 | female | 43 | health | |  |  |  |  |
| ZBYBWS01P02A09 | male | 47 | health | |  |  |  |  |
| ZBYBWS01P02A10 | male | 42 | health | |  |  |  |  |
| ZBYBWS01P02A11 | male | 30 | health | |  |  |  |  |
| ZBYBWS01P02A12 | male | 38 | health | |  |  |  |  |
| ZBYBWS01P02B01 | male | 33 | health | |  |  |  |  |
| ZBYBWS01P02B02 | male | 29 | health | |  |  |  |  |
| ZBYBWS01P02B03 | male | 42 | health | |  |  |  |  |
| ZBYBWS01P02B04 | female | 28 | health | |  |  |  |  |
| ZBYBWS01P02B05 | female | 38 | health | |  |  |  |  |
| ZBYBWS01P02B06 | male | 30 | health | |  |  |  |  |
| ZBYBWS01P02B07 | male | 48 | health | |  |  |  |  |
| ZBYBWS01P02B08 | male | 37 | health | |  |  |  |  |
| ZBYBWS01P02B09 | female | 31 | health | |  |  |  |  |
| ZBYBWS01P02B10 | female | 24 | health | |  |  |  |  |
| ZBYBWS01P02B11 | male | 38 | health | |  |  |  |  |
| ZBYBWS01P02B12 | male | 28 | health | |  |  |  |  |
| ZBYBWS01P02C01 | male | 29 | health | |  |  |  |  |
| ZBYBWS01P02C02 | male | 29 | health | |  |  |  |  |
| ZBYBWS01P02C03 | male | 32 | health | |  |  |  |  |
| ZBYBWS01P02C04 | female | 54 | health | |  |  |  |  |
| ZBYBWS01P02C05 | female | 31 | health | |  |  |  |  |
| ZBYBWS01P02C06 | female | 30 | health | |  |  |  |  |
| ZBYBWS01P02C07 | male | 28 | health | |  |  |  |  |
| ZBYBWS01P02C08 | male | 26 | health | |  |  |  |  |
| ZBYBWS01P02C09 | female | 27 | health | |  |  |  |  |
| ZBYBWS01P02C10 | male | 44 | health | |  |  |  |  |
| ZBYBWS01P02C11 | female | 32 | health | |  |  |  |  |
| ZBYBWS01P02C12 | male | 31 | health | |  |  |  |  |
| ZBYBWS01P02D01 | male | 55 | health | |  |  |  |  |
| ZBYBWS01P02D02 | female | 45 | health | |  |  |  |  |
| ZBYBWS01P02D03 | female | 32 | health | |  |  |  |  |
| ZBYBWS01P02D04 | male | 55 | health | |  |  |  |  |
| ZBYBWS01P02D05 | female | 53 | health | |  |  |  |  |
| ZBYBWS01P02D06 | male | 53 | health | |  |  |  |  |
| ZBYBWS01P02D07 | female | 43 | health | |  |  |  |  |
| ZBYBWS01P02D08 | female | 47 | health | |  |  |  |  |
| ZBYBWS01P02D09 | male | 42 | health | |  |  |  |  |
| ZBYBWS01P02D10 | male | 26 | health | |  |  |  |  |
| ZBYBWS01P02D11 | female | 25 | health | |  |  |  |  |
| ZBYBWS01P02D12 | male | 45 | health | |  |  |  |  |
| ZBYBWS01P02E01 | female | 28 | health | |  |  |  |  |
| ZBYBWS01P02E02 | male | 25 | health | |  |  |  |  |
| ZBYBWS01P02E03 | male | 31 | health | |  |  |  |  |
| ZBYBWS01P02E04 | male | 32 | health | |  |  |  |  |
| ZBYBWS01P02E05 | male | 54 | health | |  |  |  |  |
| ZBYBWS01P02E06 | female | 66 | health | |  |  |  |  |
| ZBYBWS01P02E07 | male | 59 | health | |  |  |  |  |
| ZBYBWS01P02E09 | male | 28 | health | |  |  |  |  |
| ZBYBWS01P02E10 | female | 48 | health | |  |  |  |  |
| ZBYBWS01P02E11 | male | 59 | health | |  |  |  |  |
| ZBYBWS01P02E12 | male | 30 | health | |  |  |  |  |
| ZBYBWS01P02F01 | female | 26 | health | |  |  |  |  |
| ZBYBWS01P02F02 | female | 49 | health | |  |  |  |  |
| ZBYBWS01P02F03 | male | 53 | health | |  |  |  |  |
| ZBYBWS01P02F04 | female | 30 | health | |  |  |  |  |
| ZBYBWS01P02F05 | female | 35 | health | |  |  |  |  |
| ZBYBWS01P02F06 | male | 52 | health | |  |  |  |  |
| ZBYBWS01P02F07 | female | 60 | health | |  |  |  |  |
| ZBYBWS01P02F08 | female | 75 | health | |  |  |  |  |
| ZBYBWS01P02F09 | male | 42 | health | |  |  |  |  |
| ZBYBWS01P02F10 | female | 27 | health | |  |  |  |  |
| ZBYBWS01P02F11 | male | 52 | health | |  |  |  |  |
| ZBYBWS01P02F12 | female | 61 | health | |  |  |  |  |
| ZBYBWS01P02G01 | female | 35 | health | |  |  |  |  |
| ZBYBWS01P02G02 | female | 40 | health | |  |  |  |  |
| ZBYBWS01P02G03 | female | 41 | health | |  |  |  |  |
| ZBYBWS01P02G04 | male | 79 | health | |  |  |  |  |
| ZBYBWS01P02G05 | female | 55 | health | |  |  |  |  |
| ZBYBWS01P02G06 | female | 25 | health | |  |  |  |  |
| ZBYBWS01P02G07 | male | 53 | health | |  |  |  |  |
| ZBYBWS01P02G08 | female | 76 | health | |  |  |  |  |
| ZBYBWS01P02G09 | male | 31 | health | |  |  |  |  |
| ZBYBWS01P02G10 | female | 30 | health | |  |  |  |  |
| ZBYBWS01P02G11 | female | 30 | health | |  |  |  |  |
| ZBYBWS01P05A02 | male | 25 | health | |  |  |  |  |
| ZBYBWS01P05A04 | male | 30 | health | |  |  |  |  |
| ZBYBWS01P05A05 | female | 30 | health | |  |  |  |  |
| ZBYBWS01P05A06 | male | 30 | health | |  |  |  |  |
| ZBYBWS01P05A08 | female | 29 | health | |  |  |  |  |
| ZBYBWS01P05A09 | male | 28 | health | |  |  |  |  |
| ZBYBWS01P05A10 | male | 57 | health | |  |  |  |  |
| ZBYBWS01P05A11 | female | 26 | health | |  |  |  |  |
| ZBYBWS01P05A12 | male | 29 | health | |  |  |  |  |
| ZBYBWS01P05B01 | male | 37 | health | |  |  |  |  |
| ZBYBWS01P05B02 | female | 39 | health | |  |  |  |  |
| ZBYBWS01P05B03 | female | 25 | health | |  |  |  |  |
| ZBYBWS01P05B04 | female | 30 | health | |  |  |  |  |
| ZBYBWS01P05B05 | male | 27 | health | |  |  |  |  |
| ZBYBWS01P05B06 | male | 37 | health | |  |  |  |  |
| ZBYBWS01P05B07 | female | 26 | health | |  |  |  |  |
| ZBYBWS01P05B08 | male | 33 | health | |  |  |  |  |
| ZBYBWS01P05B09 | female | 24 | health | |  |  |  |  |
| ZBYBWS01P05B10 | female | 22 | health | |  |  |  |  |
| ZBYBWS01P05B12 | female | 31 | health | |  |  |  |  |
| ZBYBWS01P05C01 | male | 53 | health | |  |  |  |  |
| ZBYBWS01P05C02 | male | 35 | health | |  |  |  |  |
| ZBYBWS01P05C03 | male | 34 | health | |  |  |  |  |
| ZBYBWS01P05C04 | male | 23 | health | |  |  |  |  |
| ZBYBWS01P05C05 | female | 35 | health | |  |  |  |  |
| ZBYBWS01P05C06 | female | 28 | health | |  |  |  |  |
| ZBYBWS01P05C07 | female | 27 | health | |  |  |  |  |
| ZBYBWS01P05C08 | female | 48 | health | |  |  |  |  |
| ZBYBWS01P05C09 | male | 28 | health | |  |  |  |  |
| ZBYBWS01P05C10 | female | 35 | health | |  |  |  |  |
| ZBYBWS01P05C11 | male | 28 | health | |  |  |  |  |
| ZBYBWS01P05C12 | male | 23 | health | |  |  |  |  |
| ZBYBWS01P05D01 | male | 24 | health | |  |  |  |  |
| ZBYBWS01P05D02 | male | 35 | health | |  |  |  |  |
| ZBYBWS01P05D03 | female | 28 | health | |  |  |  |  |
| ZBYBWS01P05D04 | female | 43 | health | |  |  |  |  |
| ZBYBWS01P05D05 | male | 33 | health | |  |  |  |  |
| ZBYBWS01P05D06 | male | 27 | health | |  |  |  |  |
| ZBYBWS01P05D07 | female | 27 | health | |  |  |  |  |
| ZBYBWS01P05D08 | female | 39 | health | |  |  |  |  |
| ZBYBWS01P05D09 | female | 26 | health | |  |  |  |  |
| ZBYBWS01P05D10 | male | 30 | health | |  |  |  |  |
| ZBYBWS01P05D11 | female | 30 | health | |  |  |  |  |
| ZBYBWS01P05D12 | female | 32 | health | |  |  |  |  |
| ZBYBWS01P05E01 | female | 23 | health | |  |  |  |  |
| ZBYBWS01P05E02 | female | 42 | health | |  |  |  |  |
| ZBYBWS01P05E04 | female | 24 | health | |  |  |  |  |
| ZBYBWS01P05E05 | male | 25 | health | |  |  |  |  |
| ZBYBWS01P05E06 | male | 28 | health | |  |  |  |  |
| ZBYBWS01P05E07 | female | 27 | health | |  |  |  |  |
| ZBYBWS01P05E08 | male | 45 | health | |  |  |  |  |
| ZBYBWS01P05E09 | male | 36 | health | |  |  |  |  |
| ZBYBWS01P05E10 | female | 26 | health | |  |  |  |  |
| ZBYBWS01P05E11 | male | 42 | health | |  |  |  |  |
| ZBYBWS01P05E12 | male | 66 | health | |  |  |  |  |
| ZBYBWS01P05F01 | male | 29 | health | |  |  |  |  |
| ZBYBWS01P05F02 | male | 29 | health | |  |  |  |  |
| ZBYBWS01P05F03 | male | 30 | health | |  |  |  |  |
| ZBYBWS01P05F04 | male | 34 | health | |  |  |  |  |
| ZBYBWS01P05F05 | male | 31 | health | |  |  |  |  |
| ZBYBWS01P05F06 | female | 31 | health | |  |  |  |  |
| ZBYBWS01P05F07 | female | 25 | health | |  |  |  |  |
| ZBYBWS01P05F08 | female | 29 | health | |  |  |  |  |
| ZBYBWS01P05F09 | female | 49 | health | |  |  |  |  |
| ZBYBWS01P05F10 | male | 31 | health | |  |  |  |  |
| ZBYBWS01P05F11 | male | 46 | health | |  |  |  |  |
| ZBYBWS01P05F12 | male | 27 | health | |  |  |  |  |
| ZBYBWS01P05G01 | male | 28 | health | |  |  |  |  |
| ZBYBWS01P05G02 | female | 28 | health | |  |  |  |  |
| ZBYBWS01P05G03 | male | 31 | health | |  |  |  |  |
| ZBYBWS01P05G04 | male | 28 | health | |  |  |  |  |
| ZBYBWS01P05G05 | male | 33 | health | |  |  |  |  |
| ZBYBWS01P05G06 | female | 30 | health | |  |  |  |  |
| ZBYBWS01P05G07 | male | 25 | health | |  |  |  |  |
| ZBYBWS01P05G08 | male | 30 | health | |  |  |  |  |
| ZBYBWS01P05G09 | male | 29 | health | |  |  |  |  |
| ZBYBWS01P05G10 | male | 32 | health | |  |  |  |  |
| ZBYBWS01P05G11 | female | 29 | health | |  |  |  |  |
| ZBYBWS01P05G12 | male | 23 | health | |  |  |  |  |
| ZBYBWS01P05H01 | male | 21 | health | |  |  |  |  |
| ZBYBWS01P05H02 | male | 24 | health | |  |  |  |  |
| ZBYBWS01P05H03 | male | 28 | health | |  |  |  |  |
| ZBYBWS01P05H04 | male | 25 | health | |  |  |  |  |
| ZBYBWS01P05H05 | male | 30 | health | |  |  |  |  |
| ZBYBWS01P05H06 | male | 30 | health | |  |  |  |  |
| ZBYBWS01P05H07 | female | 31 | health | |  |  |  |  |
| ZBYBWS01P05H08 | male | 25 | health | |  |  |  |  |
| ZBYBWS01P05H09 | male | 38 | health | |  |  |  |  |
| ZBYBWS01P05H10 | male | 25 | health | |  |  |  |  |
| ZBYBWS01P05H11 | male | 70 | health | |  |  |  |  |
| ZBYBWS01P05H12 | female | 39 | health | |  |  |  |  |
| ZBYBWS01P06A02 | male | 30 | health | |  |  |  |  |
| ZBYBWS01P06A03 | male | 17 | health | |  |  |  |  |
| ZBYBWS01P06A04 | male | 47 | health | |  |  |  |  |
| ZBYBWS01P06A05 | male | 24 | health | |  |  |  |  |
| ZBYBWS01P06A06 | female | 25 | health | |  |  |  |  |
| ZBYBWS01P06A07 | female | 40 | health | |  |  |  |  |
| ZBYBWS01P06A08 | female | 25 | health | |  |  |  |  |
| ZBYBWS01P06A09 | male | 31 | health | |  |  |  |  |
| ZBYBWS01P06A10 | female | 22 | health | |  |  |  |  |
| ZBYBWS01P06A11 | female | 34 | health | |  |  |  |  |
| ZBYBWS01P06A12 | female | 38 | health | |  |  |  |  |
| ZBYBWS01P06B01 | male | 58 | health | |  |  |  |  |
| ZBYBWS01P06B02 | female | 38 | health | |  |  |  |  |
| ZBYBWS01P06B03 | female | 40 | health | |  |  |  |  |
| ZBYBWS01P06B04 | male | 70 | health | |  |  |  |  |
| ZBYBWS01P06B05 | male | 33 | health | |  |  |  |  |
| ZBYBWS01P06B06 | female | 47 | health | |  |  |  |  |
| ZBYBWS01P06B07 | female | 44 | health | |  |  |  |  |
| ZBYBWS01P06B08 | female | 26 | health | |  |  |  |  |
| ZBYBWS01P06B09 | female | 31 | health | |  |  |  |  |
| ZBYBWS01P06B10 | male | 45 | health | |  |  |  |  |
| ZBYBWS01P06B11 | female | 36 | health | |  |  |  |  |
| ZBYBWS01P06B12 | male | 65 | health | |  |  |  |  |
| ZBYBWS01P06C01 | male | 47 | health | |  |  |  |  |
| ZBYBWS01P06C02 | female | 31 | health | |  |  |  |  |
| ZBYBWS01P06C03 | female | 40 | health | |  |  |  |  |
| ZBYBWS01P06C04 | female | 61 | health | |  |  |  |  |
| ZBYBWS01P06C06 | female | 28 | health | |  |  |  |  |
| ZBYBWS01P06C07 | male | 29 | health | |  |  |  |  |
| ZBYBWS01P06C08 | male | 58 | health | |  |  |  |  |
| ZBYBWS01P06C09 | female | 29 | health | |  |  |  |  |
| ZBYBWS01P06C10 | male | 47 | health | |  |  |  |  |
| ZBYBWS01P06C11 | male | 34 | health | |  |  |  |  |
| ZBYBWS01P06C12 | female | 35 | health | |  |  |  |  |
| ZBYBWS01P06D01 | male | 29 | health | |  |  |  |  |
| ZBYBWS01P06D02 | female | 23 | health | |  |  |  |  |
| ZBYBWS01P06D03 | female | 27 | health | |  |  |  |  |
| ZBYBWS01P06D04 | male | 27 | health | |  |  |  |  |
| ZBYBWS01P06D05 | female | 30 | health | |  |  |  |  |
| ZBYBWS01P06D06 | male | 35 | health | |  |  |  |  |
| ZBYBWS01P06D07 | female | 29 | health | |  |  |  |  |
| ZBYBWS01P06D08 | male | 34 | health | |  |  |  |  |
| ZBYBWS01P06D10 | female | 25 | health | |  |  |  |  |
| ZBYBWS01P06D11 | male | 30 | health | |  |  |  |  |
| ZBYBWS01P06D12 | male | 24 | health | |  |  |  |  |
| ZBYBWS01P06E01 | male | 39 | health | |  |  |  |  |
| ZBYBWS01P06E02 | female | 31 | health | |  |  |  |  |
| ZBYBWS01P06E03 | male | 45 | health | |  |  |  |  |
| ZBYBWS01P06E04 | male | 49 | health | |  |  |  |  |
| ZBYBWS01P06E05 | female | 43 | health | |  |  |  |  |
| ZBYBWS01P06E06 | female | 46 | health | |  |  |  |  |
| ZBYBWS01P06E07 | male | 42 | health | |  |  |  |  |
| ZBYBWS01P06E08 | female | 53 | health | |  |  |  |  |
| ZBYBWS01P06E09 | male | 44 | health | |  |  |  |  |
| ZBYBWS01P06E10 | male | 43 | health | |  |  |  |  |
| ZBYBWS01P06E11 | female | 34 | health | |  |  |  |  |
| ZBYBWS01P06E12 | male | 53 | health | |  |  |  |  |
| ZBYBWS01P06F01 | female | 38 | health | |  |  |  |  |
| ZBYBWS01P06F02 | male | 59 | health | |  |  |  |  |
| ZBYBWS01P06F03 | male | 24 | health | |  |  |  |  |
| ZBYBWS01P06F04 | male | 32 | health | |  |  |  |  |
| ZBYBWS01P06F05 | female | 25 | health | |  |  |  |  |
| ZBYBWS01P06F06 | male | 32 | health | |  |  |  |  |
| ZBYBWS01P06F07 | male | 48 | health | |  |  |  |  |
| ZBYBWS01P06F08 | female | 45 | health | |  |  |  |  |
| ZBYBWS01P06F09 | male | 52 | health | |  |  |  |  |
| ZBYBWS01P06F10 | male | 44 | health | |  |  |  |  |
| ZBYBWS01P06F11 | male | 41 | health | |  |  |  |  |
| ZBYBWS01P06F12 | male | 53 | health | |  |  |  |  |
| ZBYBWS01P06G01 | male | 37 | health | |  |  |  |  |
| ZBYBWS01P06G02 | male | 29 | health | |  |  |  |  |
| ZBYBWS01P06G03 | male | 26 | health | |  |  |  |  |
| ZBYBWS01P06G04 | female | 35 | health | |  |  |  |  |
| ZBYBWS01P06G05 | male | 47 | health | |  |  |  |  |
| ZBYBWS01P06G06 | female | 28 | health | |  |  |  |  |
| ZBYBWS01P06G07 | female | 32 | health | |  |  |  |  |
| ZBYBWS01P06G08 | female | 35 | health | |  |  |  |  |
| ZBYBWS01P06G09 | female | 30 | health | |  |  |  |  |
| ZBYBWS01P06G10 | female | 35 | health | |  |  |  |  |
| ZBYBWS01P06G11 | female | 45 | health | |  |  |  |  |
| ZBYBWS01P06G12 | male | 35 | health | |  |  |  |  |
| ZBYBWS01P06H01 | male | 38 | health | |  |  |  |  |
| ZBYBWS01P06H02 | female | 32 | health | |  |  |  |  |
| ZBYBWS01P06H03 | male | 40 | health | |  |  |  |  |
| ZBYBWS01P06H04 | female | 39 | health | |  |  |  |  |
| ZBYBWS01P06H05 | female | 35 | health | |  |  |  |  |
| ZBYBWS01P06H06 | male | 25 | health | |  |  |  |  |
| ZBYBWS01P06H07 | female | 31 | health | |  |  |  |  |
| ZBYBWS01P06H08 | male | 29 | health | |  |  |  |  |
| ZBYBWS01P06H09 | male | 26 | health | |  |  |  |  |
| ZBYBWS01P06H10 | female | 46 | health | |  |  |  |  |
| ZBYBWS01P06H11 | female | 53 | health | |  |  |  |  |
| ZBYBWS01P06H12 | female | 27 | health | |  |  |  |  |
| ZBYBWS01P09A02 | female | 35 | health | |  |  |  |  |
| ZBYBWS01P09A03 | female | 33 | health | |  |  |  |  |
| ZBYBWS01P09A05 | male | 51 | health | |  |  |  |  |
| ZBYBWS01P09A06 | female | 34 | health | |  |  |  |  |
| ZBYBWS01P09A07 | female | 25 | health | |  |  |  |  |
| ZBYBWS01P09A08 | male | 53 | health | |  |  |  |  |
| ZBYBWS01P09A09 | female | 36 | health | |  |  |  |  |
| ZBYBWS01P09A11 | male | 42 | health | |  |  |  |  |
| ZBYBWS01P09A12 | male | 36 | health | |  |  |  |  |
| ZBYBWS01P09B01 | male | 35 | health | |  |  |  |  |
| ZBYBWS01P09B02 | female | 30 | health | |  |  |  |  |
| ZBYBWS01P09B03 | female | 35 | health | |  |  |  |  |
| ZBYBWS01P09B04 | female | 38 | health | |  |  |  |  |
| ZBYBWS01P09B05 | female | 33 | health | |  |  |  |  |
| ZBYBWS01P09B06 | female | 44 | health | |  |  |  |  |
| ZBYBWS01P09B07 | male | 33 | health | |  |  |  |  |
| ZBYBWS01P09B08 | male | 44 | health | |  |  |  |  |
| ZBYBWS01P09B09 | male | 47 | health | |  |  |  |  |
| ZBYBWS01P09B10 | female | 35 | health | |  |  |  |  |
| ZBYBWS01P09B11 | male | 30 | health | |  |  |  |  |
| ZBYBWS01P09B12 | female | 26 | health | |  |  |  |  |
| ZBYBWS01P09C01 | male | 33 | health | |  |  |  |  |
| ZBYBWS01P09C02 | male | 39 | health | |  |  |  |  |
| ZBYBWS01P09C03 | male | 34 | health | |  |  |  |  |
| ZBYBWS01P09C04 | female | 30 | health | |  |  |  |  |
| ZBYBWS01P09C05 | female | 31 | health | |  |  |  |  |
| ZBYBWS01P09C06 | male | 31 | health | |  |  |  |  |
| ZBYBWS01P09C07 | female | 27 | health | |  |  |  |  |
| ZBYBWS01P09C09 | male | 27 | health | |  |  |  |  |
| ZBYBWS01P09C10 | male | 26 | health | |  |  |  |  |
| ZBYBWS01P09C11 | male | 32 | health | |  |  |  |  |
| ZBYBWS01P09C12 | female | 33 | health | |  |  |  |  |
| ZBYBWS01P09D01 | female | 30 | health | |  |  |  |  |
| ZBYBWS01P09D03 | female | 32 | health | |  |  |  |  |
| ZBYBWS01P09D04 | female | 21 | health | |  |  |  |  |
| ZBYBWS01P09D05 | female | 40 | health | |  |  |  |  |
| ZBYBWS01P09D06 | female | 31 | health | |  |  |  |  |
| ZBYBWS01P09D07 | female | 31 | health | |  |  |  |  |
| ZBYBWS01P09D08 | male | 46 | health | |  |  |  |  |
| ZBYBWS01P09D09 | male | 37 | health | |  |  |  |  |
| ZBYBWS01P09D10 | female | 33 | health | |  |  |  |  |
| ZBYBWS01P09D12 | male | 66 | health | |  |  |  |  |
| ZBYBWS01P09E01 | female | 40 | health | |  |  |  |  |
| ZBYBWS01P09E02 | female | 30 | health | |  |  |  |  |
| ZBYBWS01P09E03 | male | 41 | health | |  |  |  |  |
| ZBYBWS01P09E04 | female | 34 | health | |  |  |  |  |
| ZBYBWS01P09E06 | female | 43 | health | |  |  |  |  |
| ZBYBWS01P09E08 | female | 25 | health | |  |  |  |  |
| ZBYBWS01P09E10 | male | 52 | health | |  |  |  |  |
| ZBYBWS01P09E11 | male | 28 | health | |  |  |  |  |
| ZBYBWS01P09E12 | male | 50 | health | |  |  |  |  |
| ZBYBWS01P09F01 | male | 39 | health | |  |  |  |  |
| ZBYBWS01P09F02 | female | 31 | health | |  |  |  |  |
| ZBYBWS01P09F03 | male | 41 | health | |  |  |  |  |
| ZBYBWS01P09F05 | female | 42 | health | |  |  |  |  |
| ZBYBWS01P09F06 | male | 28 | health | |  |  |  |  |
| ZBYBWS01P09F07 | male | 43 | health | |  |  |  |  |
| ZBYBWS01P09F08 | male | 43 | health | |  |  |  |  |
| ZBYBWS01P09F09 | male | 38 | health | |  |  |  |  |
| ZBYBWS01P09F11 | male | 52 | health | |  |  |  |  |
| ZBYBWS01P09F12 | male | 47 | health | |  |  |  |  |
| ZBYBWS01P09G01 | male | 27 | health | |  |  |  |  |
| ZBYBWS01P09G02 | male | 28 | health | |  |  |  |  |
| ZBYBWS01P09G03 | male | 44 | health | |  |  |  |  |
| ZBYBWS01P09G05 | male | 30 | health | |  |  |  |  |
| ZBYBWS01P09G06 | male | 52 | health | |  |  |  |  |
| ZBYBWS01P09G07 | male | 28 | health | |  |  |  |  |
| ZBYBWS01P09G08 | female | 34 | health | |  |  |  |  |
| ZBYBWS01P09G09 | male | 50 | health | |  |  |  |  |
| ZBYBWS01P09G10 | male | 28 | health | |  |  |  |  |
| ZBYBWS01P09G11 | female | 28 | health | |  |  |  |  |
| ZBYBWS01P09G12 | male | 57 | health | |  |  |  |  |
| ZBYBWS01P09H01 | female | 23 | health | |  |  |  |  |
| ZBYBWS01P09H02 | female | 23 | health | |  |  |  |  |
| ZBYBWS01P09H03 | female | 31 | health | |  |  |  |  |
| ZBYBWS01P09H04 | female | 31 | health | |  |  |  |  |
| ZBYBWS01P09H05 | female | 45 | health | |  |  |  |  |
| ZBYBWS01P09H06 | male | 29 | health | |  |  |  |  |
| ZBYBWS01P09H07 | female | 31 | health | |  |  |  |  |
| ZBYBWS01P09H08 | female | 28 | health | |  |  |  |  |
| ZBYBWS01P09H09 | male | 40 | health | |  |  |  |  |
| ZBYBWS01P09H10 | male | 26 | health | |  |  |  |  |
| ZBYBWS01P09H11 | male | 25 | health | |  |  |  |  |
| ZBYBWS01P09H12 | female | 30 | health | |  |  |  |  |
| ZBYBWS01P10A02 | male | 29 | health | |  |  |  |  |
| ZBYBWS01P10A03 | male | 42 | health | |  |  |  |  |
| ZBYBWS01P10A04 | female | 46 | health | |  |  |  |  |
| ZBYBWS01P10A05 | male | 44 | health | |  |  |  |  |
| ZBYBWS01P10A06 | female | 34 | health | |  |  |  |  |
| ZBYBWS01P10A07 | male | 42 | health | |  |  |  |  |
| ZBYBWS01P10A08 | female | 33 | health | |  |  |  |  |
| ZBYBWS01P10A09 | male | 64 | health | |  |  |  |  |
| ZBYBWS01P10A10 | male | 56 | health | |  |  |  |  |
| ZBYBWS01P10A11 | female | 44 | health | |  |  |  |  |
| ZBYBWS01P10A12 | female | 30 | health | |  |  |  |  |
| ZBYBWS01P10B01 | female | 41 | health | |  |  |  |  |
| ZBYBWS01P10B02 | male | 37 | health | |  |  |  |  |
| ZBYBWS01P10B04 | male | 28 | health | |  |  |  |  |
| ZBYBWS01P10B06 | male | 51 | health | |  |  |  |  |
| ZBYBWS01P10B07 | female | 30 | health | |  |  |  |  |
| ZBYBWS01P10B08 | female | 41 | health | |  |  |  |  |
| ZBYBWS01P10B09 | female | 28 | health | |  |  |  |  |
| ZBYBWS01P10B10 | female | 36 | health | |  |  |  |  |
| ZBYBWS01P10B11 | male | 52 | health | |  |  |  |  |
| ZBYBWS01P10B12 | male | 47 | health | |  |  |  |  |
| ZBYBWS01P10C01 | male | 28 | health | |  |  |  |  |
| ZBYBWS01P10C02 | male | 53 | health | |  |  |  |  |
| ZBYBWS01P10C03 | male | 54 | health | |  |  |  |  |
| ZBYBWS01P10C04 | female | 39 | health | |  |  |  |  |
| ZBYBWS01P10C05 | female | 53 | health | |  |  |  |  |
| ZBYBWS01P10C06 | male | 48 | health | |  |  |  |  |
| ZBYBWS01P10C07 | male | 45 | health | |  |  |  |  |
| ZBYBWS01P10C08 | male | 24 | health | |  |  |  |  |
| ZBYBWS01P10C09 | male | 28 | health | |  |  |  |  |
| ZBYBWS01P10C10 | male | 46 | health | |  |  |  |  |
| ZBYBWS01P10D04 | male | 60 | health | |  |  |  |  |
| ZBYBWS01P10D08 | male | 29 | health | |  |  |  |  |
| ZBYBWS01P10D10 | female | 28 | health | |  |  |  |  |
| ZBYBWS01P10D11 | male | 43 | health | |  |  |  |  |
| ZBYBWS01P10D12 | female | 49 | health | |  |  |  |  |
| ZBYBWS01P10E01 | male | 56 | health | |  |  |  |  |
| ZBYBWS01P10E02 | male | 38 | health | |  |  |  |  |
| ZBYBWS01P10E03 | male | 58 | health | |  |  |  |  |
| ZBYBWS01P10E04 | male | 46 | health | |  |  |  |  |
| ZBYBWS01P10E05 | male | 32 | health | |  |  |  |  |
| ZBYBWS01P10E06 | female | 28 | health | |  |  |  |  |
| ZBYBWS01P10E07 | female | 44 | health | |  |  |  |  |
| ZBYBWS01P10E08 | male | 48 | health | |  |  |  |  |
| ZBYBWS01P10E09 | male | 34 | health | |  |  |  |  |
| ZBYBWS01P10E10 | female | 38 | health | |  |  |  |  |
| ZBYBWS01P10E11 | female | 45 | health | |  |  |  |  |
| ZBYBWS01P10E12 | male | 48 | health | |  |  |  |  |
| ZBYBWS01P10F01 | female | 40 | health | |  |  |  |  |
| ZBYBWS01P10F02 | female | 29 | health | |  |  |  |  |
| ZBYBWS01P10F03 | female | 60 | health | |  |  |  |  |
| ZBYBWS01P10F04 | female | 47 | health | |  |  |  |  |
| ZBYBWS01P10F05 | male | 35 | health | |  |  |  |  |
| ZBYBWS01P10F06 | male | 45 | health | |  |  |  |  |
| ZBYBWS01P10F07 | female | 24 | health | |  |  |  |  |
| ZBYBWS01P10F08 | male | 49 | health | |  |  |  |  |
| ZBYBWS01P10F09 | male | 25 | health | |  |  |  |  |
| ZBYBWS01P10F10 | female | 30 | health | |  |  |  |  |
| ZBYBWS01P10F11 | female | 39 | health | |  |  |  |  |
| ZBYBWS01P10F12 | female | 59 | health | |  |  |  |  |
| ZBYBWS01P10G01 | male | 29 | health | |  |  |  |  |
| ZBYBWS01P10G02 | male | 50 | health | |  |  |  |  |
| ZBYBWS01P10G03 | male | 39 | health | |  |  |  |  |
| ZBYBWS01P10G04 | male | 30 | health | |  |  |  |  |
| ZBYBWS01P10G05 | male | 26 | health | |  |  |  |  |
| ZBYBWS01P10G06 | female | 37 | health | |  |  |  |  |
| ZBYBWS01P10G07 | female | 29 | health | |  |  |  |  |
| ZBYBWS01P10G08 | female | 37 | health | |  |  |  |  |
| ZBYBWS01P10G09 | male | 47 | health | |  |  |  |  |
| ZBYBWS01P10G10 | female | 32 | health | |  |  |  |  |
| ZBYBWS01P10G11 | male | 30 | health | |  |  |  |  |
| ZBYBWS01P10G12 | male | 36 | health | |  |  |  |  |
| ZBYBWS01P10H01 | female | 34 | health | |  |  |  |  |
| ZBYBWS01P10H02 | male | 54 | health | |  |  |  |  |
| ZBYBWS01P10H03 | male | 42 | health | |  |  |  |  |
| ZBYBWS01P10H04 | male | 35 | health | |  |  |  |  |
| ZBYBWS01P10H05 | female | 22 | health | |  |  |  |  |
| ZBYBWS01P10H06 | female | 29 | health | |  |  |  |  |
| ZBYBWS01P10H07 | male | 34 | health | |  |  |  |  |
| ZBYBWS01P10H08 | female | 34 | health | |  |  |  |  |
| ZBYBWS01P10H09 | male | 46 | health | |  |  |  |  |
| ZBYBWS01P10H10 | male | 47 | health | |  |  |  |  |
| ZBYBWS01P10H11 | female | 43 | health | |  |  |  |  |
| ZBYBWS01P10H12 | female | 26 | health | |  |  |  |  |
| ZBYBWS01P13A02 | male | 60 | health | |  |  |  |  |
| ZBYBWS01P13A03 | female | 33 | health | |  |  |  |  |
| ZBYBWS01P13A04 | male | 30 | health | |  |  |  |  |
| ZBYBWS01P13A05 | female | 37 | health | |  |  |  |  |
| ZBYBWS01P13A06 | male | 55 | health | |  |  |  |  |
| ZBYBWS01P13A07 | male | 31 | health | |  |  |  |  |
| ZBYBWS01P13A08 | male | 59 | health | |  |  |  |  |
| ZBYBWS01P13A09 | male | 40 | health | |  |  |  |  |
| ZBYBWS01P13A10 | female | 64 | health | |  |  |  |  |
| ZBYBWS01P13A11 | male | 40 | health | |  |  |  |  |
| ZBYBWS01P13A12 | male | 58 | health | |  |  |  |  |
| ZBYBWS01P13B01 | male | 39 | health | |  |  |  |  |
| ZBYBWS01P13B02 | male | 32 | health | |  |  |  |  |
| ZBYBWS01P13B03 | male | 27 | health | |  |  |  |  |
| ZBYBWS01P13B04 | female | 30 | health | |  |  |  |  |
| ZBYBWS01P13B05 | male | 50 | health | |  |  |  |  |
| ZBYBWS01P13B06 | male | 25 | health | |  |  |  |  |
| ZBYBWS01P13B07 | male | 33 | health | |  |  |  |  |
| ZBYBWS01P13B08 | male | 26 | health | |  |  |  |  |
| ZBYBWS01P13B09 | male | 39 | health | |  |  |  |  |
| ZBYBWS01P13B10 | male | 45 | health | |  |  |  |  |
| ZBYBWS01P13B11 | female | 41 | health | |  |  |  |  |
| ZBYBWS01P13B12 | male | 29 | health | |  |  |  |  |
| ZBYBWS01P13C01 | male | 30 | health | |  |  |  |  |
| ZBYBWS01P13C02 | female | 26 | health | |  |  |  |  |
| ZBYBWS01P13C03 | female | 45 | health | |  |  |  |  |
| ZBYBWS01P13C04 | male | 35 | health | |  |  |  |  |
| ZBYBWS01P13C05 | female | 30 | health | |  |  |  |  |
| ZBYBWS01P13C06 | female | 53 | health | |  |  |  |  |
| ZBYBWS01P13C07 | female | 45 | health | |  |  |  |  |
| ZBYBWS01P13C08 | male | 53 | health | |  |  |  |  |
| ZBYBWS01P13C10 | male | 33 | health | |  |  |  |  |
| ZBYBWS01P13C11 | female | 25 | health | |  |  |  |  |
| ZBYBWS01P13C12 | female | 28 | health | |  |  |  |  |
| ZBYBWS01P13D01 | male | 42 | health | |  |  |  |  |
| ZBYBWS01P13D02 | female | 31 | health | |  |  |  |  |
| ZBYBWS01P13D03 | male | 51 | health | |  |  |  |  |
| ZBYBWS01P13D04 | female | 34 | health | |  |  |  |  |
| ZBYBWS01P13D05 | male | 46 | health | |  |  |  |  |
| ZBYBWS01P13D06 | male | 26 | health | |  |  |  |  |
| ZBYBWS01P13D07 | male | 35 | health | |  |  |  |  |
| ZBYBWS01P13D08 | male | 40 | health | |  |  |  |  |
| ZBYBWS01P13D09 | male | 50 | health | |  |  |  |  |
| ZBYBWS01P13D10 | female | 42 | health | |  |  |  |  |
| ZBYBWS01P13D11 | male | 34 | health | |  |  |  |  |
| ZBYBWS01P13D12 | male | 47 | health | |  |  |  |  |
| ZBYBWS01P13E01 | female | 25 | health | |  |  |  |  |
| ZBYBWS01P13E02 | male | 42 | health | |  |  |  |  |
| ZBYBWS01P13E03 | female | 28 | health | |  |  |  |  |
| ZBYBWS01P13E04 | male | 49 | health | |  |  |  |  |
| ZBYBWS01P13E05 | female | 30 | health | |  |  |  |  |
| ZBYBWS01P13E06 | female | 48 | health | |  |  |  |  |
| ZBYBWS01P13E07 | female | 29 | health | |  |  |  |  |
| ZBYBWS01P13E08 | female | 50 | health | |  |  |  |  |
| ZBYBWS01P13E09 | female | 33 | health | |  |  |  |  |
| ZBYBWS01P13E10 | male | 40 | health | |  |  |  |  |
| ZBYBWS01P13E11 | male | 45 | health | |  |  |  |  |
| ZBYBWS01P13E12 | female | 42 | health | |  |  |  |  |
| ZBYBWS01P13F01 | female | 30 | health | |  |  |  |  |
| ZBYBWS01P13F02 | female | 36 | health | |  |  |  |  |
| ZBYBWS01P13F03 | female | 35 | health | |  |  |  |  |
| ZBYBWS01P13F04 | female | 30 | health | |  |  |  |  |
| ZBYBWS01P13F05 | female | 46 | health | |  |  |  |  |
| ZBYBWS01P13F06 | male | 46 | health | |  |  |  |  |
| ZBYBWS01P13F07 | male | 30 | health | |  |  |  |  |
| ZBYBWS01P13F08 | female | 58 | health | |  |  |  |  |
| ZBYBWS01P13F09 | male | 52 | health | |  |  |  |  |
| ZBYBWS01P13F10 | female | 24 | health | |  |  |  |  |
| ZBYBWS01P13F11 | male | 41 | health | |  |  |  |  |
| ZBYBWS01P13F12 | male | 37 | health | |  |  |  |  |
| ZBYBWS01P13G01 | female | 34 | health | |  |  |  |  |
| ZBYBWS01P13G02 | male | 31 | health | |  |  |  |  |
| ZBYBWS01P13G03 | female | 38 | health | |  |  |  |  |
| ZBYBWS01P13G04 | female | 25 | health | |  |  |  |  |
| ZBYBWS01P13G05 | female | 28 | health | |  |  |  |  |
| ZBYBWS01P13G06 | female | 80 | health | |  |  |  |  |
| ZBYBWS01P13G07 | female | 43 | health | |  |  |  |  |
| ZBYBWS01P13G08 | female | 23 | health | |  |  |  |  |
| ZBYBWS01P13G09 | male | 33 | health | |  |  |  |  |
| ZBYBWS01P13G10 | male | 32 | health | |  |  |  |  |
| ZBYBWS01P13G11 | male | 68 | health | |  |  |  |  |
| ZBYBWS01P13G12 | female | 53 | health | |  |  |  |  |
| ZBYBWS01P13H01 | female | 34 | health | |  |  |  |  |
| ZBYBWS01P13H02 | female | 21 | health | |  |  |  |  |
| ZBYBWS01P13H03 | female | 33 | health | |  |  |  |  |
| ZBYBWS01P13H04 | female | 27 | health | |  |  |  |  |
| ZBYBWS01P13H05 | female | 30 | health | |  |  |  |  |
| ZBYBWS01P13H06 | male | 71 | health | |  |  |  |  |
| ZBYBWS01P13H07 | male | 29 | health | |  |  |  |  |
| ZBYBWS01P13H08 | male | 68 | health | |  |  |  |  |
| ZBYBWS01P13H09 | male | 27 | health | |  |  |  |  |
| ZBYBWS01P13H10 | male | 22 | health | |  |  |  |  |
| ZBYBWS01P13H11 | female | 33 | health | |  |  |  |  |
| ZBYBWS01P13H12 | female | 77 | health | |  |  |  |  |
| ZBYBWS01P14A02 | female | 34 | health | |  |  |  |  |
| ZBYBWS01P14A03 | male | 61 | health | |  |  |  |  |
| ZBYBWS01P14A04 | male | 28 | health | |  |  |  |  |
| ZBYBWS01P14A05 | female | 20 | health | |  |  |  |  |
| ZBYBWS01P14A06 | male | 34 | health | |  |  |  |  |
| ZBYBWS01P14A07 | female | 26 | health | |  |  |  |  |
| ZBYBWS01P14A08 | male | 62 | health | |  |  |  |  |
| ZBYBWS01P14A09 | female | 48 | health | |  |  |  |  |
| ZBYBWS01P14A10 | male | 34 | health | |  |  |  |  |
| ZBYBWS01P14A11 | male | 38 | health | |  |  |  |  |
| ZBYBWS01P14A12 | female | 43 | health | |  |  |  |  |
| ZBYBWS01P14B01 | female | 44 | health | |  |  |  |  |
| ZBYBWS01P14B02 | male | 59 | health | |  |  |  |  |
| ZBYBWS01P14B03 | male | 30 | health | |  |  |  |  |
| ZBYBWS01P14B04 | male | 64 | health | |  |  |  |  |
| ZBYBWS01P14B05 | female | 24 | health | |  |  |  |  |
| ZBYBWS01P14B06 | male | 43 | health | |  |  |  |  |
| ZBYBWS01P14B07 | male | 27 | health | |  |  |  |  |
| ZBYBWS01P14B08 | male | 32 | health | |  |  |  |  |
| ZBYBWS01P14B09 | male | 60 | health | |  |  |  |  |
| ZBYBWS01P14B10 | female | 34 | health | |  |  |  |  |
| ZBYBWS01P14B11 | female | 40 | health | |  |  |  |  |
| ZBYBWS01P14B12 | female | 25 | health | |  |  |  |  |
| ZBYBWS01P14C01 | female | 48 | health | |  |  |  |  |
| ZBYBWS01P14C02 | male | 48 | health | |  |  |  |  |
| ZBYBWS01P14C03 | female | 52 | health | |  |  |  |  |
| ZBYBWS01P14C04 | male | 47 | health | |  |  |  |  |
| ZBYBWS01P14C05 | female | 28 | health | |  |  |  |  |
| ZBYBWS01P14C06 | male | 29 | health | |  |  |  |  |
| ZBYBWS01P14C07 | male | 63 | health | |  |  |  |  |
| ZBYBWS01P14C08 | female | 48 | health | |  |  |  |  |
| ZBYBWS01P14C09 | female | 26 | health | |  |  |  |  |
| ZBYBWS01P14C10 | male | 32 | health | |  |  |  |  |
| ZBYBWS01P14C11 | female | 60 | health | |  |  |  |  |
| ZBYBWS01P14C12 | male | 76 | health | |  |  |  |  |
| ZBYBWS01P14D01 | male | 73 | health | |  |  |  |  |
| ZBYBWS01P14D02 | female | 27 | health | |  |  |  |  |
| ZBYBWS01P14D03 | male | 54 | health | |  |  |  |  |
| ZBYBWS01P14D04 | male | 63 | health | |  |  |  |  |
| ZBYBWS01P14D05 | female | 24 | health | |  |  |  |  |
| ZBYBWS01P14D06 | male | 50 | health | |  |  |  |  |
| ZBYBWS01P14D07 | male | 42 | health | |  |  |  |  |
| ZBYBWS01P14D08 | male | 28 | health | |  |  |  |  |
| ZBYBWS01P14D09 | female | 30 | health | |  |  |  |  |
| ZBYBWS01P14D10 | male | 36 | health | |  |  |  |  |
| ZBYBWS01P14D11 | female | 33 | health | |  |  |  |  |
| ZBYBWS01P14D12 | female | 29 | health | |  |  |  |  |
| ZBYBWS01P14E01 | female | 27 | health | |  |  |  |  |
| ZBYBWS01P14E02 | male | 30 | health | |  |  |  |  |
| ZBYBWS01P14E03 | female | 32 | health | |  |  |  |  |
| ZBYBWS01P14E04 | male | 23 | health | |  |  |  |  |
| ZBYBWS01P14E05 | female | 33 | health | |  |  |  |  |
| ZBYBWS01P14E06 | female | 26 | health | |  |  |  |  |
| ZBYBWS01P14E07 | female | 30 | health | |  |  |  |  |
| ZBYBWS01P14E08 | male | 33 | health | |  |  |  |  |
| ZBYBWS01P14E09 | female | 33 | health | |  |  |  |  |
| ZBYBWS01P14E10 | male | 36 | health | |  |  |  |  |
| ZBYBWS01P14E11 | female | 37 | health | |  |  |  |  |
| ZBYBWS01P14E12 | female | 24 | health | |  |  |  |  |
| ZBYBWS01P14F01 | female | 36 | health | |  |  |  |  |
| ZBYBWS01P14F02 | female | 37 | health | |  |  |  |  |
| ZBYBWS01P14F03 | male | 25 | health | |  |  |  |  |
| ZBYBWS01P14F04 | female | 39 | health | |  |  |  |  |
| ZBYBWS01P14F05 | female | 27 | health | |  |  |  |  |
| ZBYBWS01P14F06 | male | 29 | health | |  |  |  |  |
| ZBYBWS01P14F07 | female | 27 | health | |  |  |  |  |
| ZBYBWS01P14F08 | female | 36 | health | |  |  |  |  |
| ZBYBWS01P14F09 | female | 30 | health | |  |  |  |  |
| ZBYBWS01P14F10 | male | 31 | health | |  |  |  |  |
| ZBYBWS01P14F11 | female | 38 | health | |  |  |  |  |
| ZBYBWS01P14F12 | female | 31 | health | |  |  |  |  |
| ZBYBWS01P14G01 | male | 24 | health | |  |  |  |  |
| ZBYBWS01P14G02 | female | 56 | health | |  |  |  |  |
| ZBYBWS01P14G03 | female | 27 | health | |  |  |  |  |
| ZBYBWS01P14G04 | female | 45 | health | |  |  |  |  |
| ZBYBWS01P14G05 | female | 32 | health | |  |  |  |  |
| ZBYBWS01P14G06 | female | 30 | health | |  |  |  |  |
| ZBYBWS01P14G07 | male | 26 | health | |  |  |  |  |
| ZBYBWS01P14G08 | male | 24 | health | |  |  |  |  |
| ZBYBWS01P14G09 | female | 25 | health | |  |  |  |  |
| ZBYBWS01P14G10 | male | 25 | health | |  |  |  |  |
| ZBYBWS01P14G11 | female | 53 | health | |  |  |  |  |
| ZBYBWS01P14G12 | male | 26 | health | |  |  |  |  |
| ZBYBWS01P14H01 | female | 39 | health | |  |  |  |  |
| ZBYBWS01P14H02 | female | 42 | health | |  |  |  |  |
| ZBYBWS01P14H03 | female | 43 | health | |  |  |  |  |
| ZBYBWS01P14H04 | male | 46 | health | |  |  |  |  |
| ZBYBWS01P14H05 | male | 61 | health | |  |  |  |  |
| ZBYBWS01P14H06 | female | 46 | health | |  |  |  |  |
| ZBYBWS01P14H07 | male | 29 | health | |  |  |  |  |
| ZBYBWS01P14H08 | female | 38 | health | |  |  |  |  |
| ZBYBWS01P14H09 | female | 35 | health | |  |  |  |  |
| ZBYBWS01P14H10 | female | 38 | health | |  |  |  |  |
| ZBYBWS01P14H11 | male | 22 | health | |  |  |  |  |
| ZBYBWS01P14H12 | male | 25 | health | |  |  |  |  |
| ZBYBWS01P17A02 | male | 24 | health | |  |  |  |  |
| ZBYBWS01P17A03 | male | 30 | health | |  |  |  |  |
| ZBYBWS01P17A04 | female | 30 | health | |  |  |  |  |
| ZBYBWS01P17A05 | female | 54 | health | |  |  |  |  |
| ZBYBWS01P17A06 | male | 31 | health | |  |  |  |  |
| ZBYBWS01P17A07 | male | 28 | health | |  |  |  |  |
| ZBYBWS01P17A08 | female | 43 | health | |  |  |  |  |
| ZBYBWS01P17A09 | male | 43 | health | |  |  |  |  |
| ZBYBWS01P17A10 | female | 25 | health | |  |  |  |  |
| ZBYBWS01P17A11 | male | 38 | health | |  |  |  |  |
| ZBYBWS01P17A12 | male | 29 | health | |  |  |  |  |
| ZBYBWS01P17B01 | male | 25 | health | |  |  |  |  |
| ZBYBWS01P17B02 | male | 27 | health | |  |  |  |  |
| ZBYBWS01P17B03 | female | 53 | health | |  |  |  |  |
| ZBYBWS01P17B04 | female | 27 | health | |  |  |  |  |
| ZBYBWS01P17B05 | male | 22 | health | |  |  |  |  |
| ZBYBWS01P17B06 | female | 46 | health | |  |  |  |  |
| ZBYBWS01P17B07 | male | 64 | health | |  |  |  |  |
| ZBYBWS01P17B08 | male | 37 | health | |  |  |  |  |
| ZBYBWS01P17B09 | female | 24 | health | |  |  |  |  |
| ZBYBWS01P17B10 | female | 29 | health | |  |  |  |  |
| ZBYBWS01P17B11 | female | 36 | health | |  |  |  |  |
| ZBYBWS01P17B12 | male | 64 | health | |  |  |  |  |
| ZBYBWS01P17C01 | male | 37 | health | |  |  |  |  |
| ZBYBWS01P17C02 | female | 45 | health | |  |  |  |  |
| ZBYBWS01P17C03 | male | 26 | health | |  |  |  |  |
| ZBYBWS01P17C04 | female | 32 | health | |  |  |  |  |
| ZBYBWS01P17C05 | male | 44 | health | |  |  |  |  |
| ZBYBWS01P17C06 | male | 26 | health | |  |  |  |  |
| ZBYBWS01P17C07 | female | 27 | health | |  |  |  |  |
| ZBYBWS01P17C08 | female | 28 | health | |  |  |  |  |
| ZBYBWS01P17C09 | male | 32 | health | |  |  |  |  |
| ZBYBWS01P17C10 | male | 31 | health | |  |  |  |  |
| ZBYBWS01P17C11 | male | 34 | health | |  |  |  |  |
| ZBYBWS01P17C12 | male | 35 | health | |  |  |  |  |
| ZBYBWS01P17D01 | female | 47 | health | |  |  |  |  |
| ZBYBWS01P17D02 | male | 46 | health | |  |  |  |  |
| ZBYBWS01P17D03 | male | 28 | health | |  |  |  |  |
| ZBYBWS01P17D04 | male | 31 | health | |  |  |  |  |
| ZBYBWS01P17D05 | male | 34 | health | |  |  |  |  |
| ZBYBWS01P17D06 | male | 52 | health | |  |  |  |  |
| ZBYBWS01P17D07 | male | 36 | health | |  |  |  |  |
| ZBYBWS01P17D08 | male | 29 | health | |  |  |  |  |
| ZBYBWS01P17D09 | male | 27 | health | |  |  |  |  |
| ZBYBWS01P17D10 | female | 30 | health | |  |  |  |  |
| ZBYBWS01P17D11 | male | 39 | health | |  |  |  |  |
| ZBYBWS01P17D12 | male | 41 | health | |  |  |  |  |
| ZBYBWS01P17E01 | female | 46 | health | |  |  |  |  |
| ZBYBWS01P17E02 | female | 34 | health | |  |  |  |  |
| ZBYBWS01P17E03 | male | 38 | health | |  |  |  |  |
| ZBYBWS01P17E04 | female | 23 | health | |  |  |  |  |
| ZBYBWS01P17E05 | female | 32 | health | |  |  |  |  |
| ZBYBWS01P17E06 | female | 58 | health | |  |  |  |  |
| ZBYBWS01P17E07 | male | 54 | health | |  |  |  |  |
| ZBYBWS01P17E08 | male | 45 | health | |  |  |  |  |
| ZBYBWS01P17E09 | female | 46 | health | |  |  |  |  |
| ZBYBWS01P17E10 | male | 41 | health | |  |  |  |  |
| ZBYBWS01P17E11 | female | 35 | health | |  |  |  |  |
| ZBYBWS01P17E12 | female | 25 | health | |  |  |  |  |
| ZBYBWS01P17F01 | male | 23 | health | |  |  |  |  |
| ZBYBWS01P17F02 | female | 41 | health | |  |  |  |  |
| ZBYBWS01P17F03 | female | 34 | health | |  |  |  |  |
| ZBYBWS01P17F04 | female | 50 | health | |  |  |  |  |
| ZBYBWS01P17F05 | female | 37 | health | |  |  |  |  |
| ZBYBWS01P17F06 | female | 36 | health | |  |  |  |  |
| ZBYBWS01P17F07 | female | 32 | health | |  |  |  |  |
| ZBYBWS01P17F08 | female | 35 | health | |  |  |  |  |
| ZBYBWS01P17F09 | female | 36 | health | |  |  |  |  |
| ZBYBWS01P17F10 | female | 28 | health | |  |  |  |  |
| ZBYBWS01P17F11 | male | 42 | health | |  |  |  |  |
| ZBYBWS01P17F12 | female | 44 | health | |  |  |  |  |
| ZBYBWS01P17G01 | male | 52 | health | |  |  |  |  |
| ZBYBWS01P17G02 | female | 29 | health | |  |  |  |  |
| ZBYBWS01P17G03 | female | 49 | health | |  |  |  |  |
| ZBYBWS01P17G04 | male | 34 | health | |  |  |  |  |
| ZBYBWS01P17G05 | male | 32 | health | |  |  |  |  |
| ZBYBWS01P17G06 | male | 41 | health | |  |  |  |  |
| ZBYBWS01P17G07 | male | 25 | health | |  |  |  |  |
| ZBYBWS01P17G08 | female | 28 | health | |  |  |  |  |
| ZBYBWS01P17G09 | male | 30 | health | |  |  |  |  |
| ZBYBWS01P17G10 | male | 24 | health | |  |  |  |  |
| ZBYBWS01P17G11 | male | 24 | health | |  |  |  |  |
| ZBYBWS01P17G12 | female | 27 | health | |  |  |  |  |
| ZBYBWS01P17H01 | male | 32 | health | |  |  |  |  |
| ZBYBWS01P17H03 | male | 42 | health | |  |  |  |  |
| ZBYBWS01P17H04 | male | 27 | health | |  |  |  |  |
| ZBYBWS01P17H05 | male | 34 | health | |  |  |  |  |
| ZBYBWS01P17H06 | male | 51 | health | |  |  |  |  |
| ZBYBWS01P17H07 | female | 26 | health | |  |  |  |  |
| ZBYBWS01P17H08 | female | 45 | health | |  |  |  |  |
| ZBYBWS01P17H09 | female | 22 | health | |  |  |  |  |
| ZBYBWS01P17H10 | male | 33 | health | |  |  |  |  |
| ZBYBWS01P17H11 | female | 43 | health | |  |  |  |  |
| ZBYBWS01P17H12 | male | 35 | health | |  |  |  |  |
| ZBYBWS01P18A02 | female | 50 | health | |  |  |  |  |
| ZBYBWS01P18A03 | female | 40 | health | |  |  |  |  |
| ZBYBWS01P18A04 | female | 36 | health | |  |  |  |  |
| ZBYBWS01P18A05 | female | 52 | health | |  |  |  |  |
| ZBYBWS01P18A06 | male | 34 | health | |  |  |  |  |
| ZBYBWS01P18A07 | female | 36 | health | |  |  |  |  |
| ZBYBWS01P18A08 | male | 27 | health | |  |  |  |  |
| ZBYBWS01P18A09 | male | 39 | health | |  |  |  |  |
| ZBYBWS01P18A10 | male | 38 | health | |  |  |  |  |
| ZBYBWS01P18A11 | male | 31 | health | |  |  |  |  |
| ZBYBWS01P18A12 | male | 32 | health | |  |  |  |  |
| ZBYBWS01P18B01 | male | 47 | health | |  |  |  |  |
| ZBYBWS01P18B02 | female | 49 | health | |  |  |  |  |
| ZBYBWS01P18B03 | female | 29 | health | |  |  |  |  |
| ZBYBWS01P18B04 | male | 43 | health | |  |  |  |  |
| ZBYBWS01P18B05 | female | 48 | health | |  |  |  |  |
| ZBYBWS01P18B06 | male | 43 | health | |  |  |  |  |
| ZBYBWS01P18B07 | female | 40 | health | |  |  |  |  |
| ZBYBWS01P18B08 | male | 33 | health | |  |  |  |  |
| ZBYBWS01P18B09 | male | 46 | health | |  |  |  |  |
| ZBYBWS01P18B10 | female | 46 | health | |  |  |  |  |
| ZBYBWS01P18B11 | female | 33 | health | |  |  |  |  |
| ZBYBWS01P18B12 | female | 40 | health | |  |  |  |  |
| ZBYBWS01P18C01 | male | 58 | health | |  |  |  |  |
| ZBYBWS01P18C02 | female | 54 | health | |  |  |  |  |
| ZBYBWS01P18C03 | male | 23 | health | |  |  |  |  |
| ZBYBWS01P18C04 | female | 30 | health | |  |  |  |  |
| ZBYBWS01P18C05 | male | 25 | health | |  |  |  |  |
| ZBYBWS01P18C06 | male | 30 | health | |  |  |  |  |
| ZBYBWS01P18C07 | female | 35 | health | |  |  |  |  |
| ZBYBWS01P18C08 | female | 28 | health | |  |  |  |  |
| ZBYBWS01P18C09 | male | 33 | health | |  |  |  |  |
| ZBYBWS01P18C10 | female | 25 | health | |  |  |  |  |
| ZBYBWS01P18C11 | male | 31 | health | |  |  |  |  |
| ZBYBWS01P18C12 | female | 29 | health | |  |  |  |  |
| ZBYBWS01P18D01 | male | 44 | health | |  |  |  |  |
| ZBYBWS01P18D02 | female | 41 | health | |  |  |  |  |
| ZBYBWS01P18D03 | male | 46 | health | |  |  |  |  |
| ZBYBWS01P18D04 | female | 28 | health | |  |  |  |  |
| ZBYBWS01P18D05 | female | 30 | health | |  |  |  |  |
| ZBYBWS01P18D06 | female | 22 | health | |  |  |  |  |
| ZBYBWS01P18D07 | male | 28 | health | |  |  |  |  |
| ZBYBWS01P18D08 | female | 35 | health | |  |  |  |  |
| ZBYBWS01P18D09 | female | 30 | health | |  |  |  |  |
| ZBYBWS01P18D10 | female | 26 | health | |  |  |  |  |
| ZBYBWS01P18D11 | female | 29 | health | |  |  |  |  |
| ZBYBWS01P18D12 | female | 32 | health | |  |  |  |  |
| ZBYBWS01P18E01 | female | 45 | health | |  |  |  |  |
| ZBYBWS01P18E02 | male | 51 | health | |  |  |  |  |
| ZBYBWS01P18E03 | male | 30 | health | |  |  |  |  |
| ZBYBWS01P18E04 | female | 39 | health | |  |  |  |  |
| ZBYBWS01P18E05 | male | 24 | health | |  |  |  |  |
| ZBYBWS01P18E06 | male | 32 | health | |  |  |  |  |
| ZBYBWS01P18E07 | male | 40 | health | |  |  |  |  |
| ZBYBWS01P18E08 | female | 31 | health | |  |  |  |  |
| ZBYBWS01P18E09 | male | 25 | health | |  |  |  |  |
| ZBYBWS01P18E10 | female | 24 | health | |  |  |  |  |
| ZBYBWS01P18E11 | male | 31 | health | |  |  |  |  |
| ZBYBWS01P18E12 | male | 29 | health | |  |  |  |  |
| ZBYBWS01P18F01 | female | 51 | health | |  |  |  |  |
| ZBYBWS01P18F02 | female | 29 | health | |  |  |  |  |
| ZBYBWS01P18F03 | female | 36 | health | |  |  |  |  |
| ZBYBWS01P18F04 | male | 24 | health | |  |  |  |  |
| ZBYBWS01P18F05 | male | 46 | health | |  |  |  |  |
| ZBYBWS01P18F06 | female | 36 | health | |  |  |  |  |
| ZBYBWS01P18F07 | female | 29 | health | |  |  |  |  |
| ZBYBWS01P18F08 | male | 28 | health | |  |  |  |  |
| ZBYBWS01P18F09 | female | 34 | health | |  |  |  |  |
| ZBYBWS01P18F10 | female | 27 | health | |  |  |  |  |
| ZBYBWS01P18F11 | male | 37 | health | |  |  |  |  |
| ZBYBWS01P18F12 | female | 29 | health | |  |  |  |  |
| ZBYBWS01P18G01 | male | 26 | health | |  |  |  |  |
| ZBYBWS01P18G02 | female | 30 | health | |  |  |  |  |
| ZBYBWS01P18G03 | male | 29 | health | |  |  |  |  |
| ZBYBWS01P18G04 | male | 33 | health | |  |  |  |  |
| ZBYBWS01P18G05 | female | 30 | health | |  |  |  |  |
| ZBYBWS01P18G06 | female | 31 | health | |  |  |  |  |
| ZBYBWS01P18G07 | female | 43 | health | |  |  |  |  |
| ZBYBWS01P18G08 | male | 35 | health | |  |  |  |  |
| ZBYBWS01P18G09 | male | 25 | health | |  |  |  |  |
| ZBYBWS01P18G10 | female | 27 | health | |  |  |  |  |
| ZBYBWS01P18G11 | male | 61 | health | |  |  |  |  |
| ZBYBWS01P18G12 | male | 53 | health | |  |  |  |  |
| ZBYBWS01P18H01 | female | 31 | health | |  |  |  |  |
| ZBYBWS01P18H02 | male | 33 | health | |  |  |  |  |
| ZBYBWS01P18H03 | male | 35 | health | |  |  |  |  |
| ZBYBWS01P18H04 | female | 34 | health | |  |  |  |  |
| ZBYBWS01P18H05 | female | 31 | health | |  |  |  |  |
| ZBYBWS01P18H06 | female | 26 | health | |  |  |  |  |
| ZBYBWS01P18H07 | female | 28 | health | |  |  |  |  |
| ZBYBWS01P18H08 | male | 30 | health | |  |  |  |  |
| ZBYBWS01P18H09 | female | 31 | health | |  |  |  |  |
| ZBYBWS01P18H10 | male | 22 | health | |  |  |  |  |
| ZBYBWS01P18H11 | male | 30 | health | |  |  |  |  |
| ZBYBWS01P18H12 | female | 31 | health | |  |  |  |  |
| ZBYBWS01P21A02 | male | 80 | health | |  |  |  |  |
| ZBYBWS01P21A03 | female | 26 | health | |  |  |  |  |
| ZBYBWS01P21A04 | female | 50 | health | |  |  |  |  |
| ZBYBWS01P21A05 | female | 29 | health | |  |  |  |  |
| ZBYBWS01P21A06 | male | 28 | health | |  |  |  |  |
| ZBYBWS01P21A07 | male | 31 | health | |  |  |  |  |
| ZBYBWS01P21A08 | female | 31 | health | |  |  |  |  |
| ZBYBWS01P21A09 | female | 34 | health | |  |  |  |  |
| ZBYBWS01P21A10 | female | 32 | health | |  |  |  |  |
| ZBYBWS01P21A11 | female | 29 | health | |  |  |  |  |
| ZBYBWS01P21A12 | male | 43 | health | |  |  |  |  |
| ZBYBWS01P21B01 | female | 29 | health | |  |  |  |  |
| ZBYBWS01P21B02 | male | 77 | health | |  |  |  |  |
| ZBYBWS01P21B03 | male | 50 | health | |  |  |  |  |
| ZBYBWS01P21B04 | male | 28 | health | |  |  |  |  |
| ZBYBWS01P21B05 | male | 41 | health | |  |  |  |  |
| ZBYBWS01P21B06 | female | 24 | health | |  |  |  |  |
| ZBYBWS01P21B07 | female | 30 | health | |  |  |  |  |
| ZBYBWS01P21B08 | female | 38 | health | |  |  |  |  |
| ZBYBWS01P21B09 | female | 48 | health | |  |  |  |  |
| ZBYBWS01P21B10 | male | 40 | health | |  |  |  |  |
| ZBYBWS01P21B11 | female | 39 | health | |  |  |  |  |
| ZBYBWS01P21B12 | female | 32 | health | |  |  |  |  |
| ZBYBWS01P21C01 | male | 50 | health | |  |  |  |  |
| ZBYBWS01P21C02 | male | 23 | health | |  |  |  |  |
| ZBYBWS01P21C03 | male | 28 | health | |  |  |  |  |
| ZBYBWS01P21C04 | female | 27 | health | |  |  |  |  |
| ZBYBWS01P21C06 | female | 45 | health | |  |  |  |  |
| ZBYBWS01P21C07 | male | 33 | health | |  |  |  |  |
| ZBYBWS01P21C08 | male | 27 | health | |  |  |  |  |
| ZBYBWS01P21C09 | male | 37 | health | |  |  |  |  |
| ZBYBWS01P21C10 | female | 38 | health | |  |  |  |  |
| ZBYBWS01P21C11 | male | 42 | health | |  |  |  |  |
| ZBYBWS01P21C12 | female | 34 | health | |  |  |  |  |
| ZBYBWS01P21D01 | male | 46 | health | |  |  |  |  |
| ZBYBWS01P21D02 | female | 47 | health | |  |  |  |  |
| ZBYBWS01P21D03 | female | 38 | health | |  |  |  |  |
| ZBYBWS01P21D04 | male | 41 | health | |  |  |  |  |
| ZBYBWS01P21D05 | male | 49 | health | |  |  |  |  |
| ZBYBWS01P21D06 | male | 31 | health | |  |  |  |  |
| ZBYBWS01P21D07 | male | 28 | health | |  |  |  |  |
| ZBYBWS01P21D08 | male | 25 | health | |  |  |  |  |
| ZBYBWS01P21D09 | female | 32 | health | |  |  |  |  |
| ZBYBWS01P21D10 | male | 31 | health | |  |  |  |  |
| ZBYBWS01P21D11 | female | 41 | health | |  |  |  |  |
| ZBYBWS01P21D12 | male | 26 | health | |  |  |  |  |
| ZBYBWS01P21E01 | female | 39 | health | |  |  |  |  |
| ZBYBWS01P21E02 | male | 44 | health | |  |  |  |  |
| ZBYBWS01P21E03 | male | 56 | health | |  |  |  |  |
| ZBYBWS01P21E04 | female | 25 | health | |  |  |  |  |
| ZBYBWS01P21E05 | male | 23 | health | |  |  |  |  |
| ZBYBWS01P21E06 | male | 30 | health | |  |  |  |  |
| ZBYBWS01P21E07 | male | 38 | health | |  |  |  |  |
| ZBYBWS01P21E08 | male | 35 | health | |  |  |  |  |
| ZBYBWS01P21E09 | male | 53 | health | |  |  |  |  |
| ZBYBWS01P21E10 | male | 24 | health | |  |  |  |  |
| ZBYBWS01P21E11 | male | 29 | health | |  |  |  |  |
| ZBYBWS01P21E12 | male | 49 | health | |  |  |  |  |
| ZBYBWS01P21F01 | male | 28 | health | |  |  |  |  |
| ZBYBWS01P21F02 | male | 41 | health | |  |  |  |  |
| ZBYBWS01P21F03 | male | 39 | health | |  |  |  |  |
| ZBYBWS01P21F04 | male | 30 | health | |  |  |  |  |
| ZBYBWS01P21F05 | female | 30 | health | |  |  |  |  |
| ZBYBWS01P21F06 | female | 36 | health | |  |  |  |  |
| ZBYBWS01P21F07 | male | 53 | health | |  |  |  |  |
| ZBYBWS01P21F08 | male | 33 | health | |  |  |  |  |
| ZBYBWS01P21F09 | male | 27 | health | |  |  |  |  |
| ZBYBWS01P21F10 | male | 41 | health | |  |  |  |  |
| ZBYBWS01P21F11 | female | 31 | health | |  |  |  |  |
| ZBYBWS01P21F12 | male | 32 | health | |  |  |  |  |
| ZBYBWS01P21G01 | male | 25 | health | |  |  |  |  |
| ZBYBWS01P21G02 | male | 32 | health | |  |  |  |  |
| ZBYBWS01P21G03 | female | 24 | health | |  |  |  |  |
| ZBYBWS01P21G04 | male | 40 | health | |  |  |  |  |
| ZBYBWS01P21G05 | female | 35 | health | |  |  |  |  |
| ZBYBWS01P21G06 | male | 49 | health | |  |  |  |  |
| ZBYBWS01P21G07 | male | 48 | health | |  |  |  |  |
| ZBYBWS01P21G08 | female | 33 | health | |  |  |  |  |
| ZBYBWS01P21G09 | female | 36 | health | |  |  |  |  |
| ZBYBWS01P21G10 | male | 32 | health | |  |  |  |  |
| ZBYBWS01P21G11 | male | 26 | health | |  |  |  |  |
| ZBYBWS01P21G12 | female | 42 | health | |  |  |  |  |
| ZBYBWS01P21H02 | female | 25 | health | |  |  |  |  |
| ZBYBWS01P21H04 | female | 59 | health | |  |  |  |  |
| ZBYBWS01P21H06 | male | 50 | health | |  |  |  |  |
| ZBYBWS01P21H10 | male | 28 | health | |  |  |  |  |
| ZBYBWS01P21H11 | male | 25 | health | |  |  |  |  |
| ZBYBWS01P22A02 | male | 50 | health | |  |  |  |  |
| ZBYBWS01P22A03 | female | 25 | health | |  |  |  |  |
| ZBYBWS01P22A04 | male | 31 | health | |  |  |  |  |
| ZBYBWS01P22A05 | male | 27 | health | |  |  |  |  |
| ZBYBWS01P22A06 | male | 39 | health | |  |  |  |  |
| ZBYBWS01P22A08 | male | 42 | health | |  |  |  |  |
| ZBYBWS01P22A09 | female | 34 | health | |  |  |  |  |
| ZBYBWS01P22A10 | female | 28 | health | |  |  |  |  |
| ZBYBWS01P22A11 | female | 39 | health | |  |  |  |  |
| ZBYBWS01P22A12 | male | 31 | health | |  |  |  |  |
| ZBYBWS01P22B01 | male | 49 | health | |  |  |  |  |
| ZBYBWS01P22B02 | male | 53 | health | |  |  |  |  |
| ZBYBWS01P22B03 | female | 48 | health | |  |  |  |  |
| ZBYBWS01P22B04 | female | 51 | health | |  |  |  |  |
| ZBYBWS01P22B05 | male | 41 | health | |  |  |  |  |
| ZBYBWS01P22B07 | female | 36 | health | |  |  |  |  |
| ZBYBWS01P22B10 | female | 29 | health | |  |  |  |  |
| ZBYBWS01P22C01 | male | 52 | health | |  |  |  |  |
| ZBYBWS01P22C02 | female | 30 | health | |  |  |  |  |
| ZBYBWS01P22C03 | female | 24 | health | |  |  |  |  |
| ZBYBWS01P22C05 | male | 43 | health | |  |  |  |  |
| ZBYBWS01P22C06 | male | 40 | health | |  |  |  |  |
| ZBYBWS01P22C07 | male | 39 | health | |  |  |  |  |
| ZBYBWS01P22C08 | female | 28 | health | |  |  |  |  |
| ZBYBWS01P22C09 | female | 24 | health | |  |  |  |  |
| ZBYBWS01P22C10 | male | 40 | health | |  |  |  |  |
| ZBYBWS01P22C11 | male | 32 | health | |  |  |  |  |
| ZBYBWS01P22C12 | male | 46 | health | |  |  |  |  |
| ZBYBWS01P22D01 | female | 36 | health | |  |  |  |  |
| ZBYBWS01P22D03 | female | 48 | health | |  |  |  |  |
| ZBYBWS01P22D04 | male | 47 | health | |  |  |  |  |
| ZBYBWS01P22D05 | male | 47 | health | |  |  |  |  |
| ZBYBWS01P22D06 | male | 38 | health | |  |  |  |  |
| ZBYBWS01P22D07 | male | 44 | health | |  |  |  |  |
| ZBYBWS01P22D08 | female | 26 | health | |  |  |  |  |
| ZBYBWS01P22D09 | male | 44 | health | |  |  |  |  |
| ZBYBWS01P22D10 | male | 33 | health | |  |  |  |  |
| ZBYBWS01P22D11 | male | 39 | health | |  |  |  |  |
| ZBYBWS01P22D12 | male | 53 | health | |  |  |  |  |
| ZBYBWS01P22E01 | male | 28 | health | |  |  |  |  |
| ZBYBWS01P22E02 | female | 46 | health | |  |  |  |  |
| ZBYBWS01P22E04 | male | 49 | health | |  |  |  |  |
| ZBYBWS01P22E05 | male | 32 | health | |  |  |  |  |
| ZBYBWS01P22E06 | male | 30 | health | |  |  |  |  |
| ZBYBWS01P22E07 | male | 53 | health | |  |  |  |  |
| ZBYBWS01P22E08 | female | 42 | health | |  |  |  |  |
| ZBYBWS01P22E09 | female | 37 | health | |  |  |  |  |
| ZBYBWS01P22E10 | male | 53 | health | |  |  |  |  |
| ZBYBWS01P22E11 | female | 47 | health | |  |  |  |  |
| ZBYBWS01P22E12 | female | 39 | health | |  |  |  |  |
| ZBYBWS01P22F01 | male | 46 | health | |  |  |  |  |
| ZBYBWS01P22F02 | female | 31 | health | |  |  |  |  |
| ZBYBWS01P22F03 | male | 67 | health | |  |  |  |  |
| ZBYBWS01P22F04 | female | 34 | health | |  |  |  |  |
| ZBYBWS01P22F05 | male | 40 | health | |  |  |  |  |
| ZBYBWS01P22F06 | male | 45 | health | |  |  |  |  |
| ZBYBWS01P22F08 | male | 51 | health | |  |  |  |  |
| ZBYBWS01P22F09 | male | 28 | health | |  |  |  |  |
| ZBYBWS01P22F10 | female | 25 | health | |  |  |  |  |
| ZBYBWS01P22F11 | female | 25 | health | |  |  |  |  |
| ZBYBWS01P22F12 | male | 43 | health | |  |  |  |  |
| ZBYBWS01P22G01 | female | 29 | health | |  |  |  |  |
| ZBYBWS01P22G02 | male | 52 | health | |  |  |  |  |
| ZBYBWS01P22G03 | male | 23 | health | |  |  |  |  |
| ZBYBWS01P22G05 | female | 41 | health | |  |  |  |  |
| ZBYBWS01P22G06 | male | 38 | health | |  |  |  |  |
| ZBYBWS01P22G07 | female | 30 | health | |  |  |  |  |
| ZBYBWS01P22G08 | female | 37 | health | |  |  |  |  |
| ZBYBWS01P22G09 | female | 34 | health | |  |  |  |  |
| ZBYBWS01P22G10 | female | 42 | health | |  |  |  |  |
| ZBYBWS01P22G11 | female | 38 | health | |  |  |  |  |
| ZBYBWS01P22G12 | female | 55 | health | |  |  |  |  |
| ZBYBWS01P22H01 | male | 48 | health | |  |  |  |  |
| ZBYBWS01P22H02 | male | 47 | health | |  |  |  |  |
| ZBYBWS01P22H03 | male | 56 | health | |  |  |  |  |
| ZBYBWS01P22H04 | male | 46 | health | |  |  |  |  |
| ZBYBWS01P22H05 | male | 27 | health | |  |  |  |  |
| ZBYBWS01P22H06 | male | 53 | health | |  |  |  |  |
| ZBYBWS01P22H07 | male | 50 | health | |  |  |  |  |
| ZBYBWS01P22H08 | male | 25 | health | |  |  |  |  |
| ZBYBWS01P22H09 | female | 23 | health | |  |  |  |  |
| ZBYBWS01P22H10 | female | 42 | health | |  |  |  |  |
| ZBYBWS01P22H11 | female | 44 | health | |  |  |  |  |
| ZBYBWS01P22H12 | male | 40 | health | |  |  |  |  |
| ZBYBWS01P25A02 | female | 44 | health | |  |  |  |  |
| ZBYBWS01P25A03 | female | 34 | health | |  |  |  |  |
| ZBYBWS01P25A04 | female | 39 | health | |  |  |  |  |
| ZBYBWS01P25A05 | female | 35 | health | |  |  |  |  |
| ZBYBWS01P25A06 | male | 36 | health | |  |  |  |  |
| ZBYBWS01P25A07 | male | 37 | health | |  |  |  |  |
| ZBYBWS01P25A08 | male | 41 | health | |  |  |  |  |
| ZBYBWS01P25A09 | female | 39 | health | |  |  |  |  |
| ZBYBWS01P25A10 | female | 31 | health | |  |  |  |  |
| ZBYBWS01P25A11 | male | 58 | health | |  |  |  |  |
| ZBYBWS01P25A12 | male | 47 | health | |  |  |  |  |
| ZBYBWS01P25B01 | female | 37 | health | |  |  |  |  |
| ZBYBWS01P25B02 | male | 37 | health | |  |  |  |  |
| ZBYBWS01P25B03 | female | 28 | health | |  |  |  |  |
| ZBYBWS01P25B04 | female | 45 | health | |  |  |  |  |
| ZBYBWS01P25B05 | female | 35 | health | |  |  |  |  |
| ZBYBWS01P25B06 | female | 25 | health | |  |  |  |  |
| ZBYBWS01P25B07 | female | 32 | health | |  |  |  |  |
| ZBYBWS01P25B08 | female | 40 | health | |  |  |  |  |
| ZBYBWS01P25B09 | female | 23 | health | |  |  |  |  |
| ZBYBWS01P25B10 | female | 30 | health | |  |  |  |  |
| ZBYBWS01P25B11 | male | 24 | health | |  |  |  |  |
| ZBYBWS01P25B12 | female | 45 | health | |  |  |  |  |
| ZBYBWS01P25C01 | female | 34 | health | |  |  |  |  |
| ZBYBWS01P25C02 | male | 39 | health | |  |  |  |  |
| ZBYBWS01P25C03 | female | 53 | health | |  |  |  |  |
| ZBYBWS01P25C04 | female | 42 | health | |  |  |  |  |
| ZBYBWS01P25C05 | male | 28 | health | |  |  |  |  |
| ZBYBWS01P25C06 | female | 38 | health | |  |  |  |  |
| ZBYBWS01P25C07 | male | 50 | health | |  |  |  |  |
| ZBYBWS01P25C08 | female | 21 | health | |  |  |  |  |
| ZBYBWS01P25C09 | female | 23 | health | |  |  |  |  |
| ZBYBWS01P25C10 | male | 48 | health | |  |  |  |  |
| ZBYBWS01P25C11 | female | 47 | health | |  |  |  |  |
| ZBYBWS01P25C12 | female | 32 | health | |  |  |  |  |
| ZBYBWS01P25D01 | female | 26 | health | |  |  |  |  |
| ZBYBWS01P25D02 | male | 40 | health | |  |  |  |  |
| ZBYBWS01P25D03 | female | 36 | health | |  |  |  |  |
| ZBYBWS01P25D04 | female | 30 | health | |  |  |  |  |
| ZBYBWS01P25D05 | male | 52 | health | |  |  |  |  |
| ZBYBWS01P25D06 | male | 24 | health | |  |  |  |  |
| ZBYBWS01P25D07 | male | 52 | health | |  |  |  |  |
| ZBYBWS01P25D08 | female | 35 | health | |  |  |  |  |
| ZBYBWS01P25D09 | female | 29 | health | |  |  |  |  |
| ZBYBWS01P25D10 | male | 37 | health | |  |  |  |  |
| ZBYBWS01P25D11 | male | 27 | health | |  |  |  |  |
| ZBYBWS01P25D12 | male | 46 | health | |  |  |  |  |
| ZBYBWS01P25E01 | male | 46 | health | |  |  |  |  |
| ZBYBWS01P25E02 | female | 38 | health | |  |  |  |  |
| ZBYBWS01P25E03 | male | 66 | health | |  |  |  |  |
| ZBYBWS01P25E04 | male | 49 | health | |  |  |  |  |
| ZBYBWS01P25E05 | male | 80 | health | |  |  |  |  |
| ZBYBWS01P25E06 | male | 53 | health | |  |  |  |  |
| ZBYBWS01P25E07 | male | 40 | health | |  |  |  |  |
| ZBYBWS01P25E08 | male | 65 | health | |  |  |  |  |
| ZBYBWS01P25E09 | male | 42 | health | |  |  |  |  |
| ZBYBWS01P25E10 | male | 40 | health | |  |  |  |  |
| ZBYBWS01P25E11 | male | 37 | health | |  |  |  |  |
| ZBYBWS01P25E12 | male | 51 | health | |  |  |  |  |
| ZBYBWS01P25F01 | male | 39 | health | |  |  |  |  |
| ZBYBWS01P25F02 | female | 43 | health | |  |  |  |  |
| ZBYBWS01P25F03 | male | 40 | health | |  |  |  |  |
| ZBYBWS01P25F04 | female | 32 | health | |  |  |  |  |
| ZBYBWS01P25F05 | male | 69 | health | |  |  |  |  |
| ZBYBWS01P25F06 | male | 39 | health | |  |  |  |  |
| ZBYBWS01P25F07 | male | 39 | health | |  |  |  |  |
| ZBYBWS01P25F08 | male | 28 | health | |  |  |  |  |
| ZBYBWS01P25F09 | male | 37 | health | |  |  |  |  |
| ZBYBWS01P25F10 | male | 48 | health | |  |  |  |  |
| ZBYBWS01P25F11 | male | 63 | health | |  |  |  |  |
| ZBYBWS01P25F12 | male | 44 | health | |  |  |  |  |
| ZBYBWS01P25G01 | male | 51 | health | |  |  |  |  |
| ZBYBWS01P25G02 | male | 51 | health | |  |  |  |  |
| ZBYBWS01P25G03 | male | 47 | health | |  |  |  |  |
| ZBYBWS01P25G04 | male | 73 | health | |  |  |  |  |
| ZBYBWS01P25G05 | male | 62 | health | |  |  |  |  |
| ZBYBWS01P25G06 | male | 49 | health | |  |  |  |  |
| ZBYBWS01P25G07 | male | 34 | health | |  |  |  |  |
| ZBYBWS01P25G08 | male | 67 | health | |  |  |  |  |
| ZBYBWS01P25G09 | male | 60 | health | |  |  |  |  |
| ZBYBWS01P25G10 | male | 40 | health | |  |  |  |  |
| ZBYBWS01P25G11 | male | 67 | health | |  |  |  |  |
| ZBYBWS01P25G12 | male | 37 | health | |  |  |  |  |
| ZBYBWS01P25H01 | male | 52 | health | |  |  |  |  |
| ZBYBWS01P25H03 | male | 49 | health | |  |  |  |  |
| ZBYBWS01P25H04 | male | 45 | health | |  |  |  |  |
| ZBYBWS01P25H05 | male | 48 | health | |  |  |  |  |
| ZBYBWS01P25H06 | male | 29 | health | |  |  |  |  |
| ZBYBWS01P25H07 | female | 42 | health | |  |  |  |  |
| ZBYBWS01P25H08 | male | 27 | health | |  |  |  |  |
| ZBYBWS01P25H09 | female | 39 | health | |  |  |  |  |
| ZBYBWS01P25H11 | female | 34 | health | |  |  |  |  |
| ZBYBWS01P25H12 | female | 49 | health | |  |  |  |  |
| ZBYBWS01P26A02 | male | 41 | health | |  |  |  |  |
| ZBYBWS01P26A03 | male | 49 | health | |  |  |  |  |
| ZBYBWS01P26A04 | male | 36 | health | |  |  |  |  |
| ZBYBWS01P26A05 | female | 42 | health | |  |  |  |  |
| ZBYBWS01P26A06 | male | 28 | health | |  |  |  |  |
| ZBYBWS01P26A09 | female | 60 | health | |  |  |  |  |
| ZBYBWS01P26A10 | female | 47 | health | |  |  |  |  |
| ZBYBWS01P26A11 | male | 49 | health | |  |  |  |  |
| ZBYBWS01P26A12 | male | 47 | health | |  |  |  |  |
| ZBYBWS01P26B01 | female | 53 | health | |  |  |  |  |
| ZBYBWS01P26B02 | female | 54 | health | |  |  |  |  |
| ZBYBWS01P26B03 | female | 27 | health | |  |  |  |  |
| ZBYBWS01P26B04 | male | 47 | health | |  |  |  |  |
| ZBYBWS01P26B05 | female | 51 | health | |  |  |  |  |
| ZBYBWS01P26B06 | female | 64 | health | |  |  |  |  |
| ZBYBWS01P26B07 | female | 37 | health | |  |  |  |  |
| ZBYBWS01P26B08 | female | 39 | health | |  |  |  |  |
| ZBYBWS01P26B09 | female | 40 | health | |  |  |  |  |
| ZBYBWS01P26B10 | male | 45 | health | |  |  |  |  |
| ZBYBWS01P26B11 | female | 44 | health | |  |  |  |  |
| ZBYBWS01P26B12 | female | 23 | health | |  |  |  |  |
| ZBYBWS01P26C01 | male | 39 | health | |  |  |  |  |
| ZBYBWS01P26C02 | male | 41 | health | |  |  |  |  |
| ZBYBWS01P26C03 | male | 38 | health | |  |  |  |  |
| ZBYBWS01P26C04 | female | 38 | health | |  |  |  |  |
| ZBYBWS01P26C05 | female | 43 | health | |  |  |  |  |
| ZBYBWS01P26C06 | female | 42 | health | |  |  |  |  |
| ZBYBWS01P26C08 | female | 55 | health | |  |  |  |  |
| ZBYBWS01P26C09 | female | 42 | health | |  |  |  |  |
| ZBYBWS01P26C10 | female | 22 | health | |  |  |  |  |
| ZBYBWS01P26C11 | male | 35 | health | |  |  |  |  |
| ZBYBWS01P26C12 | female | 59 | health | |  |  |  |  |
| ZBYBWS01P26D01 | male | 32 | health | |  |  |  |  |
| ZBYBWS01P26D02 | female | 41 | health | |  |  |  |  |
| ZBYBWS01P26D03 | female | 49 | health | |  |  |  |  |
| ZBYBWS01P26D04 | male | 57 | health | |  |  |  |  |
| ZBYBWS01P26D05 | female | 49 | health | |  |  |  |  |
| ZBYBWS01P26D06 | male | 49 | health | |  |  |  |  |
| ZBYBWS01P26D07 | male | 64 | health | |  |  |  |  |
| ZBYBWS01P26D08 | female | 60 | health | |  |  |  |  |
| ZBYBWS01P26D09 | male | 62 | health | |  |  |  |  |
| ZBYBWS01P26D10 | female | 60 | health | |  |  |  |  |
| ZBYBWS01P26D11 | female | 50 | health | |  |  |  |  |
| ZBYBWS01P26D12 | male | 52 | health | |  |  |  |  |
| ZBYBWS01P26E01 | female | 60 | health | |  |  |  |  |
| ZBYBWS01P26E02 | female | 39 | health | |  |  |  |  |
| ZBYBWS01P26E03 | male | 56 | health | |  |  |  |  |
| ZBYBWS01P26E04 | female | 40 | health | |  |  |  |  |
| ZBYBWS01P26E05 | male | 48 | health | |  |  |  |  |
| ZBYBWS01P26E06 | female | 45 | health | |  |  |  |  |
| ZBYBWS01P26E07 | female | 52 | health | |  |  |  |  |
| ZBYBWS01P26E08 | female | 46 | health | |  |  |  |  |
| ZBYBWS01P26E09 | female | 48 | health | |  |  |  |  |
| ZBYBWS01P26E10 | male | 49 | health | |  |  |  |  |
| ZBYBWS01P26E11 | female | 38 | health | |  |  |  |  |
| ZBYBWS01P26E12 | female | 50 | health | |  |  |  |  |
| ZBYBWS01P26F01 | female | 48 | health | |  |  |  |  |
| ZBYBWS01P26F02 | female | 27 | health | |  |  |  |  |
| ZBYBWS01P26F03 | male | 44 | health | |  |  |  |  |
| ZBYBWS01P26F04 | female | 25 | health | |  |  |  |  |
| ZBYBWS01P26F05 | female | 50 | health | |  |  |  |  |
| ZBYBWS01P26F06 | male | 23 | health | |  |  |  |  |
| ZBYBWS01P26F07 | male | 50 | health | |  |  |  |  |
| ZBYBWS01P26F08 | female | 46 | health | |  |  |  |  |
| ZBYBWS01P26F09 | female | 50 | health | |  |  |  |  |
| ZBYBWS01P26F10 | male | 50 | health | |  |  |  |  |
| ZBYBWS01P26F11 | male | 52 | health | |  |  |  |  |
| ZBYBWS01P26F12 | female | 49 | health | |  |  |  |  |
| ZBYBWS01P26G01 | female | 56 | health | |  |  |  |  |
| ZBYBWS01P26G02 | female | 33 | health | |  |  |  |  |
| ZBYBWS01P26G03 | male | 37 | health | |  |  |  |  |
| ZBYBWS01P26G04 | female | 64 | health | |  |  |  |  |
| ZBYBWS01P26G05 | male | 66 | health | |  |  |  |  |
| ZBYBWS01P26G06 | male | 65 | health | |  |  |  |  |
| ZBYBWS01P26G07 | female | 39 | health | |  |  |  |  |
| ZBYBWS01P26G08 | female | 44 | health | |  |  |  |  |
| ZBYBWS01P26G09 | male | 63 | health | |  |  |  |  |
| ZBYBWS01P26G10 | female | 48 | health | |  |  |  |  |
| ZBYBWS01P26G11 | female | 45 | health | |  |  |  |  |
| ZBYBWS01P26G12 | female | 57 | health | |  |  |  |  |
| ZBYBWS01P26H01 | male | 59 | health | |  |  |  |  |
| ZBYBWS01P26H02 | female | 49 | health | |  |  |  |  |
| ZBYBWS01P26H03 | female | 29 | health | |  |  |  |  |
| ZBYBWS01P26H04 | female | 56 | health | |  |  |  |  |
| ZBYBWS01P26H05 | female | 50 | health | |  |  |  |  |
| ZBYBWS01P26H06 | male | 41 | health | |  |  |  |  |
| ZBYBWS01P26H07 | female | 43 | health | |  |  |  |  |
| ZBYBWS01P26H08 | male | 43 | health | |  |  |  |  |
| ZBYBWS01P26H09 | male | 51 | health | |  |  |  |  |
| ZBYBWS02P07A02 | female | 25 | health | |  |  |  |  |
| ZBYBWS02P07A03 | male | 28 | health | |  |  |  |  |
| ZBYBWS02P07A04 | female | 28 | health | |  |  |  |  |
| ZBYBWS02P07A05 | male | 63 | health | |  |  |  |  |
| ZBYBWS02P07A06 | female | 23 | health | |  |  |  |  |
| ZBYBWS02P07A07 | male | 31 | health | |  |  |  |  |
| ZBYBWS02P07A08 | female | 26 | health | |  |  |  |  |
| ZBYBWS02P07A09 | male | 24 | health | |  |  |  |  |
| ZBYBWS02P07A10 | female | 35 | health | |  |  |  |  |
| ZBYBWS02P07A11 | male | 49 | health | |  |  |  |  |
| ZBYBWS02P07A12 | male | 40 | health | |  |  |  |  |
| ZBYBWS02P07B01 | male | 22 | health | |  |  |  |  |
| ZBYBWS02P07B02 | female | 22 | health | |  |  |  |  |
| ZBYBWS02P07B03 | male | 32 | health | |  |  |  |  |
| ZBYBWS02P07B04 | female | 37 | health | |  |  |  |  |
| ZBYBWS02P07B05 | male | 26 | health | |  |  |  |  |
| ZBYBWS02P07B06 | female | 30 | health | |  |  |  |  |
| ZBYBWS02P07B08 | male | 47 | health | |  |  |  |  |
| ZBYBWS02P07B09 | female | 49 | health | |  |  |  |  |
| ZBYBWS02P07B10 | female | 27 | health | |  |  |  |  |
| ZBYBWS02P07B11 | female | 28 | health | |  |  |  |  |
| ZBYBWS02P07B12 | female | 42 | health | |  |  |  |  |
| ZBYBWS02P07C01 | female | 36 | health | |  |  |  |  |
| ZBYBWS02P07C02 | male | 31 | health | |  |  |  |  |
| ZBYBWS02P07C03 | male | 60 | health | |  |  |  |  |
| ZBYBWS02P07C04 | male | 32 | health | |  |  |  |  |
| ZBYBWS02P07C05 | female | 41 | health | |  |  |  |  |
| ZBYBWS02P07C06 | male | 47 | health | |  |  |  |  |
| ZBYBWS02P07C07 | male | 36 | health | |  |  |  |  |
| ZBYBWS02P07C08 | female | 35 | health | |  |  |  |  |
| ZBYBWS02P07C09 | male | 34 | health | |  |  |  |  |
| ZBYBWS02P07C10 | female | 27 | health | |  |  |  |  |
| ZBYBWS02P07C11 | male | 46 | health | |  |  |  |  |
| ZBYBWS02P07C12 | male | 25 | health | |  |  |  |  |
| ZBYBWS02P07D01 | male | 37 | health | |  |  |  |  |
| ZBYBWS02P07D02 | male | 35 | health | |  |  |  |  |
| ZBYBWS02P07D03 | female | 21 | health | |  |  |  |  |
| ZBYBWS02P07D04 | male | 24 | health | |  |  |  |  |
| ZBYBWS02P07D05 | male | 54 | health | |  |  |  |  |
| ZBYBWS02P07D06 | male | 45 | health | |  |  |  |  |
| ZBYBWS02P07D07 | male | 61 | health | |  |  |  |  |
| ZBYBWS02P07D08 | female | 30 | health | |  |  |  |  |
| ZBYBWS02P07D09 | male | 49 | health | |  |  |  |  |
| ZBYBWS02P07D10 | male | 36 | health | |  |  |  |  |
| ZBYBWS02P07D11 | male | 31 | health | |  |  |  |  |
| ZBYBWS02P07D12 | male | 28 | health | |  |  |  |  |
| ZBYBWS02P07E01 | male | 33 | health | |  |  |  |  |
| ZBYBWS02P07E02 | male | 23 | health | |  |  |  |  |
| ZBYBWS02P07E03 | female | 31 | health | |  |  |  |  |
| ZBYBWS02P07E04 | male | 31 | health | |  |  |  |  |
| ZBYBWS02P07E05 | male | 39 | health | |  |  |  |  |
| ZBYBWS02P07E06 | male | 36 | health | |  |  |  |  |
| ZBYBWS02P07E07 | male | 59 | health | |  |  |  |  |
| ZBYBWS02P07E08 | female | 54 | health | |  |  |  |  |
| ZBYBWS02P07E09 | female | 59 | health | |  |  |  |  |
| ZBYBWS02P07E10 | male | 32 | health | |  |  |  |  |
| ZBYBWS02P07E11 | male | 45 | health | |  |  |  |  |
| ZBYBWS02P07E12 | male | 39 | health | |  |  |  |  |
| ZBYBWS02P07F01 | female | 37 | health | |  |  |  |  |
| ZBYBWS02P07F02 | male | 23 | health | |  |  |  |  |
| ZBYBWS02P07F03 | female | 55 | health | |  |  |  |  |
| ZBYBWS02P07F04 | female | 34 | health | |  |  |  |  |
| ZBYBWS02P07F05 | female | 40 | health | |  |  |  |  |
| ZBYBWS02P07F06 | female | 26 | health | |  |  |  |  |
| ZBYBWS02P07F07 | female | 35 | health | |  |  |  |  |
| ZBYBWS02P07F08 | female | 33 | health | |  |  |  |  |
| ZBYBWS02P07F09 | female | 40 | health | |  |  |  |  |
| ZBYBWS02P07F10 | male | 62 | health | |  |  |  |  |
| ZBYBWS02P07F11 | female | 49 | health | |  |  |  |  |
| ZBYBWS02P07F12 | female | 30 | health | |  |  |  |  |
| ZBYBWS02P07G01 | male | 48 | health | |  |  |  |  |
| ZBYBWS02P07G02 | male | 28 | health | |  |  |  |  |
| ZBYBWS02P07G03 | female | 54 | health | |  |  |  |  |
| ZBYBWS02P07G04 | female | 44 | health | |  |  |  |  |
| ZBYBWS02P07G05 | male | 54 | health | |  |  |  |  |
| ZBYBWS02P07G06 | male | 37 | health | |  |  |  |  |
| ZBYBWS02P07G07 | male | 47 | health | |  |  |  |  |
| ZBYBWS02P07G08 | male | 32 | health | |  |  |  |  |
| ZBYBWS02P07G09 | male | 43 | health | |  |  |  |  |
| ZBYBWS02P07G10 | male | 47 | health | |  |  |  |  |
| ZBYBWS02P07G11 | male | 38 | health | |  |  |  |  |
| ZBYBWS02P07G12 | male | 25 | health | |  |  |  |  |
| ZBYBWS02P07H01 | female | 22 | health | |  |  |  |  |
| ZBYBWS02P07H02 | female | 52 | health | |  |  |  |  |
| ZBYBWS02P07H03 | female | 22 | health | |  |  |  |  |
| ZBYBWS02P07H04 | female | 37 | health | |  |  |  |  |
| ZBYBWS02P07H05 | male | 33 | health | |  |  |  |  |
| ZBYBWS02P07H06 | male | 30 | health | |  |  |  |  |
| ZBYBWS02P07H07 | female | 28 | health | |  |  |  |  |
| ZBYBWS02P07H08 | female | 33 | health | |  |  |  |  |
| ZBYBWS02P07H09 | female | 44 | health | |  |  |  |  |
| ZBYBWS02P07H10 | male | 36 | health | |  |  |  |  |
| ZBYBWS02P07H11 | male | 30 | health | |  |  |  |  |
| ZBYBWS02P09A02 | female | 38 | health | |  |  |  |  |
| ZBYBWS02P09A03 | male | 24 | health | |  |  |  |  |
| ZBYBWS02P09A04 | male | 46 | health | |  |  |  |  |
| ZBYBWS02P09A05 | male | 42 | health | |  |  |  |  |
| ZBYBWS02P09A06 | female | 22 | health | |  |  |  |  |
| ZBYBWS02P09A07 | female | 21 | health | |  |  |  |  |
| ZBYBWS02P09A08 | female | 24 | health | |  |  |  |  |
| ZBYBWS02P09A09 | female | 21 | health | |  |  |  |  |
| ZBYBWS02P09A10 | female | 24 | health | |  |  |  |  |
| ZBYBWS02P09A11 | female | 27 | health | |  |  |  |  |
| ZBYBWS02P09A12 | female | 22 | health | |  |  |  |  |
| ZBYBWS02P09B01 | male | 21 | health | |  |  |  |  |
| ZBYBWS02P09B02 | male | 65 | health | |  |  |  |  |
| ZBYBWS02P09B03 | female | 23 | health | |  |  |  |  |
| ZBYBWS02P09B04 | male | 21 | health | |  |  |  |  |
| ZBYBWS02P09B05 | male | 23 | health | |  |  |  |  |
| ZBYBWS02P09B06 | female | 29 | health | |  |  |  |  |
| ZBYBWS02P09B07 | female | 43 | health | |  |  |  |  |
| ZBYBWS02P09B08 | male | 62 | health | |  |  |  |  |
| ZBYBWS02P09B09 | male | 28 | health | |  |  |  |  |
| ZBYBWS02P09B10 | male | 54 | health | |  |  |  |  |
| ZBYBWS02P09B11 | female | 25 | health | |  |  |  |  |
| ZBYBWS02P09B12 | female | 23 | health | |  |  |  |  |
| ZBYBWS02P09C01 | female | 39 | health | |  |  |  |  |
| ZBYBWS02P09C02 | female | 54 | health | |  |  |  |  |
| ZBYBWS02P09C03 | male | 31 | health | |  |  |  |  |
| ZBYBWS02P09C04 | male | 47 | health | |  |  |  |  |
| ZBYBWS02P09C05 | male | 49 | health | |  |  |  |  |
| ZBYBWS02P09C06 | female | 27 | health | |  |  |  |  |
| ZBYBWS02P09C07 | female | 47 | health | |  |  |  |  |
| ZBYBWS02P09C08 | female | 51 | health | |  |  |  |  |
| ZBYBWS02P09C09 | male | 52 | health | |  |  |  |  |
| ZBYBWS02P09C10 | female | 36 | health | |  |  |  |  |
| ZBYBWS02P09C11 | female | 44 | health | |  |  |  |  |
| ZBYBWS02P09C12 | female | 43 | health | |  |  |  |  |
| ZBYBWS02P09D01 | female | 43 | health | |  |  |  |  |
| ZBYBWS02P09D02 | female | 50 | health | |  |  |  |  |
| ZBYBWS02P09D03 | male | 31 | health | |  |  |  |  |
| ZBYBWS02P09D04 | female | 38 | health | |  |  |  |  |
| ZBYBWS02P09D05 | female | 54 | health | |  |  |  |  |
| ZBYBWS02P09D06 | female | 44 | health | |  |  |  |  |
| ZBYBWS02P09D07 | male | 37 | health | |  |  |  |  |
| ZBYBWS02P09D08 | female | 59 | health | |  |  |  |  |
| ZBYBWS02P09D09 | female | 34 | health | |  |  |  |  |
| ZBYBWS02P09D10 | female | 35 | health | |  |  |  |  |
| ZBYBWS02P09D11 | female | 48 | health | |  |  |  |  |
| ZBYBWS02P09D12 | female | 36 | health | |  |  |  |  |
| ZBYBWS02P09E01 | male | 42 | health | |  |  |  |  |
| ZBYBWS02P09E02 | female | 25 | health | |  |  |  |  |
| ZBYBWS02P09E03 | female | 34 | health | |  |  |  |  |
| ZBYBWS02P09E04 | female | 35 | health | |  |  |  |  |
| ZBYBWS02P09E05 | male | 34 | health | |  |  |  |  |
| ZBYBWS02P09E06 | female | 35 | health | |  |  |  |  |
| ZBYBWS02P09E07 | male | 59 | health | |  |  |  |  |
| ZBYBWS02P09E08 | male | 65 | health | |  |  |  |  |
| ZBYBWS02P09E09 | male | 32 | health | |  |  |  |  |
| ZBYBWS02P09E10 | female | 46 | health | |  |  |  |  |
| ZBYBWS02P09E11 | male | 25 | health | |  |  |  |  |
| ZBYBWS02P09E12 | female | 25 | health | |  |  |  |  |
| ZBYBWS02P09F01 | male | 36 | health | |  |  |  |  |
| ZBYBWS02P09F02 | male | 44 | health | |  |  |  |  |
| ZBYBWS02P09F03 | male | 35 | health | |  |  |  |  |
| ZBYBWS02P09F04 | female | 54 | health | |  |  |  |  |
| ZBYBWS02P09F05 | male | 49 | health | |  |  |  |  |
| ZBYBWS02P09F06 | male | 40 | health | |  |  |  |  |
| ZBYBWS02P09F07 | female | 51 | health | |  |  |  |  |
| ZBYBWS02P09F08 | female | 22 | health | |  |  |  |  |
| ZBYBWS02P09F09 | male | 18 | health | |  |  |  |  |
| ZBYBWS02P09F10 | male | 26 | health | |  |  |  |  |
| ZBYBWS02P09F11 | male | 35 | health | |  |  |  |  |
| ZBYBWS02P09F12 | female | 24 | health | |  |  |  |  |
| ZBYBWS02P09G01 | male | 40 | health | |  |  |  |  |
| ZBYBWS02P09G02 | male | 47 | health | |  |  |  |  |
| ZBYBWS02P09G03 | female | 31 | health | |  |  |  |  |
| ZBYBWS02P09G04 | female | 36 | health | |  |  |  |  |
| ZBYBWS02P09G05 | female | 27 | health | |  |  |  |  |
| ZBYBWS02P09G06 | male | 43 | health | |  |  |  |  |
| ZBYBWS02P09G07 | female | 25 | health | |  |  |  |  |
| ZBYBWS02P09G08 | female | 26 | health | |  |  |  |  |
| ZBYBWS02P09G09 | male | 45 | health | |  |  |  |  |
| ZBYBWS02P09G10 | female | 36 | health | |  |  |  |  |
| ZBYBWS02P09G11 | male | 34 | health | |  |  |  |  |
| ZBYBWS02P09G12 | female | 24 | health | |  |  |  |  |
| ZBYBWS02P09H01 | male | 30 | health | |  |  |  |  |
| ZBYBWS02P09H02 | female | 42 | health | |  |  |  |  |
| ZBYBWS02P09H03 | female | 39 | health | |  |  |  |  |
| ZBYBWS02P09H04 | male | 33 | health | |  |  |  |  |
| ZBYBWS02P09H05 | male | 55 | health | |  |  |  |  |
| ZBYBWS02P09H06 | male | 48 | health | |  |  |  |  |
| ZBYBWS02P09H07 | female | 38 | health | |  |  |  |  |
| ZBYBWS02P09H08 | male | 27 | health | |  |  |  |  |
| ZBYBWS02P09H09 | male | 25 | health | |  |  |  |  |
| ZBYBWS02P09H10 | female | 38 | health | |  |  |  |  |
| ZBYBWS02P09H11 | male | 64 | health | |  |  |  |  |
| ZBYBWS02P10A02 | male | 43 | health | |  |  |  |  |
| ZBYBWS02P10A03 | male | 52 | health | |  |  |  |  |
| ZBYBWS02P10A04 | female | 40 | health | |  |  |  |  |
| ZBYBWS02P10A05 | male | 56 | health | |  |  |  |  |
| ZBYBWS02P10A06 | male | 45 | health | |  |  |  |  |
| ZBYBWS02P10A07 | female | 42 | health | |  |  |  |  |
| ZBYBWS02P10A08 | male | 20 | health | |  |  |  |  |
| ZBYBWS02P10A09 | female | 43 | health | |  |  |  |  |
| ZBYBWS02P10A10 | male | 40 | health | |  |  |  |  |
| ZBYBWS02P10A11 | male | 29 | health | |  |  |  |  |
| ZBYBWS02P10A12 | female | 42 | health | |  |  |  |  |
| ZBYBWS02P10B01 | male | 43 | health | |  |  |  |  |
| ZBYBWS02P10B02 | male | 29 | health | |  |  |  |  |
| ZBYBWS02P10B03 | female | 34 | health | |  |  |  |  |
| ZBYBWS02P10B04 | male | 28 | health | |  |  |  |  |
| ZBYBWS02P10B05 | female | 44 | health | |  |  |  |  |
| ZBYBWS02P10B06 | female | 39 | health | |  |  |  |  |
| ZBYBWS02P10B07 | female | 48 | health | |  |  |  |  |
| ZBYBWS02P10B08 | female | 40 | health | |  |  |  |  |
| ZBYBWS02P10B09 | male | 35 | health | |  |  |  |  |
| ZBYBWS02P10B10 | male | 77 | health | |  |  |  |  |
| ZBYBWS02P10B11 | female | 45 | health | |  |  |  |  |
| ZBYBWS02P10B12 | female | 43 | health | |  |  |  |  |
| ZBYBWS02P10C01 | male | 31 | health | |  |  |  |  |
| ZBYBWS02P10C02 | male | 29 | health | |  |  |  |  |
| ZBYBWS02P10C03 | female | 40 | health | |  |  |  |  |
| ZBYBWS02P10C04 | female | 53 | health | |  |  |  |  |
| ZBYBWS02P10C05 | female | 35 | health | |  |  |  |  |
| ZBYBWS02P10C06 | male | 28 | health | |  |  |  |  |
| ZBYBWS02P10C07 | male | 31 | health | |  |  |  |  |
| ZBYBWS02P10C08 | female | 43 | health | |  |  |  |  |
| ZBYBWS02P10C09 | male | 45 | health | |  |  |  |  |
| ZBYBWS02P10C10 | female | 42 | health | |  |  |  |  |
| ZBYBWS02P10C11 | female | 29 | health | |  |  |  |  |
| ZBYBWS02P10C12 | male | 35 | health | |  |  |  |  |
| ZBYBWS02P10D01 | male | 45 | health | |  |  |  |  |
| ZBYBWS02P10D02 | female | 23 | health | |  |  |  |  |
| ZBYBWS02P10D03 | female | 45 | health | |  |  |  |  |
| ZBYBWS02P10D04 | female | 32 | health | |  |  |  |  |
| ZBYBWS02P10D05 | male | 56 | health | |  |  |  |  |
| ZBYBWS02P10D06 | female | 29 | health | |  |  |  |  |
| ZBYBWS02P10D07 | male | 28 | health | |  |  |  |  |
| ZBYBWS02P10D08 | female | 30 | health | |  |  |  |  |
| ZBYBWS02P10D09 | male | 27 | health | |  |  |  |  |
| ZBYBWS02P10D10 | female | 46 | health | |  |  |  |  |
| ZBYBWS02P10D11 | male | 40 | health | |  |  |  |  |
| ZBYBWS02P10D12 | female | 25 | health | |  |  |  |  |
| ZBYBWS02P10E01 | female | 49 | health | |  |  |  |  |
| ZBYBWS02P10E02 | female | 35 | health | |  |  |  |  |
| ZBYBWS02P10E03 | female | 33 | health | |  |  |  |  |
| ZBYBWS02P10E04 | male | 39 | health | |  |  |  |  |
| ZBYBWS02P10E05 | male | 43 | health | |  |  |  |  |
| ZBYBWS02P10E06 | female | 43 | health | |  |  |  |  |
| ZBYBWS02P10E07 | female | 53 | health | |  |  |  |  |
| ZBYBWS02P10E08 | female | 33 | health | |  |  |  |  |
| ZBYBWS02P10E09 | male | 34 | health | |  |  |  |  |
| ZBYBWS02P10E10 | male | 35 | health | |  |  |  |  |
| ZBYBWS02P10E11 | female | 34 | health | |  |  |  |  |
| ZBYBWS02P10E12 | male | 46 | health | |  |  |  |  |
| ZBYBWS02P10F01 | male | 45 | health | |  |  |  |  |
| ZBYBWS02P10F02 | male | 47 | health | |  |  |  |  |
| ZBYBWS02P10F03 | male | 51 | health | |  |  |  |  |
| ZBYBWS02P10F04 | male | 48 | health | |  |  |  |  |
| ZBYBWS02P10F05 | male | 36 | health | |  |  |  |  |
| ZBYBWS02P10F07 | male | 30 | health | |  |  |  |  |
| ZBYBWS02P10F08 | female | 48 | health | |  |  |  |  |
| ZBYBWS02P10F09 | male | 30 | health | |  |  |  |  |
| ZBYBWS02P10F10 | female | 30 | health | |  |  |  |  |
| ZBYBWS02P10F11 | female | 34 | health | |  |  |  |  |
| ZBYBWS02P10F12 | female | 52 | health | |  |  |  |  |
| ZBYBWS02P10G01 | female | 48 | health | |  |  |  |  |
| ZBYBWS02P10G02 | male | 46 | health | |  |  |  |  |
| ZBYBWS02P10G03 | male | 28 | health | |  |  |  |  |
| ZBYBWS02P10G04 | male | 34 | health | |  |  |  |  |
| ZBYBWS02P10G05 | female | 31 | health | |  |  |  |  |
| ZBYBWS02P10G06 | male | 35 | health | |  |  |  |  |
| ZBYBWS02P10G07 | female | 32 | health | |  |  |  |  |
| ZBYBWS02P10G08 | male | 25 | health | |  |  |  |  |
| ZBYBWS02P10G09 | female | 24 | health | |  |  |  |  |
| ZBYBWS02P10G10 | male | 53 | health | |  |  |  |  |
| ZBYBWS02P10G11 | male | 27 | health | |  |  |  |  |
| ZBYBWS02P10G12 | male | 47 | health | |  |  |  |  |
| ZBYBWS02P10H01 | male | 48 | health | |  |  |  |  |
| ZBYBWS02P10H02 | male | 49 | health | |  |  |  |  |
| ZBYBWS02P10H03 | male | 23 | health | |  |  |  |  |
| ZBYBWS02P10H04 | female | 31 | health | |  |  |  |  |
| ZBYBWS02P10H05 | female | 25 | health | |  |  |  |  |
| ZBYBWS02P10H06 | male | 23 | health | |  |  |  |  |
| ZBYBWS02P10H07 | female | 24 | health | |  |  |  |  |
| ZBYBWS02P10H08 | female | 24 | health | |  |  |  |  |
| ZBYBWS02P10H09 | male | 47 | health | |  |  |  |  |
| ZBYBWS02P10H10 | male | 24 | health | |  |  |  |  |
| ZBYBWS02P10H11 | male | 29 | health | |  |  |  |  |
