# Supplementary material for: Significance of LncRNA CASC8 genetic polymorphisms on the tuberculosis susceptibility in Chinese population
Source: J Clin Lab Anal. 2020 Feb 7;34(6):e23234. doi: 10.1002/jcla.23234 (PMC7307370; doi:10.1002/jcla.23234)
Supplement: Supplementary file 5 [file JCLA-34-e23234-s005.doc]

Supplementary Table： Raw data for Figrue 1 heatmap

| Probe ID | Sequence | Gene symbol | lncRNA transcript | LOG2FC | ATB 1# | ATB 2# | ATB 3# | Control 1# | Control 2# | Control 3# |
| --- | --- | --- | --- | --- | --- | --- | --- | --- | --- | --- |
| ASHGA5P019729 | TTAAAAAAAGCTTTGTGCTGATGAACATGGTCACCTTGAAAGGTGTGTTGAATATGGCAG | CASC8 | CASC8-201 | 3.086 | 7.105 | 6.757 | 6.207 | 3.447 | 3.871 | 3.494 |
| ASHGA5P018683 | AGCAATGATCAGTATCAGCTCTTTATGGAGAAAGAGCAGCTCTGGAATGAGTCATTTTTT | AC138776.1 | AC138776.1-201 | 2.643 | 5.282 | 5.128 | 5.795 | 2.750 | 2.780 | 2.747 |
| ASHGA5P030193 | TAAAAAGCCGTTTCAAGATGAAATCTTATGAGTCATTATCATGTCTTCTGAGGGCTGAGA | AC104041.1 | AC104041.1-202 | 2.249 | 7.203 | 7.223 | 7.900 | 5.355 | 4.759 | 5.468 |
| ASHGA5P047345 | GGGAACGTAACTGAAGCTCATTTTGTCTTAGTTATGGACTATGAGTCCTTAATAAATGTA | PARD3-AS1 | PARD3-AS1-201 | 2.196 | 5.147 | 5.356 | 5.439 | 3.538 | 2.786 | 3.028 |
| ASHGA5P040939 | CCAGGGCAAATTGTATGTGCTCCTTACTGGGTTTATTATAAGTGTCACATGTTTTTTATA | SLC9A3-AS1 | SLC9A3-AS1-211 | 2.187 | 13.400 | 13.529 | 13.892 | 11.243 | 11.559 | 11.457 |
| ASHGA5P027162 | TCTCTAGCTCGCCATCCACTGGTGATCTTGTTGATGTATCTGTTTGGTTACTTCCTGACT | LINC02386 | LINC02386-202 | 2.179 | 5.466 | 4.829 | 5.109 | 3.127 | 2.997 | 2.742 |
| ASHGA5P055877 | GCCAGAACATGGTGAATACAATCAAGTTCAGTGTATTTGATAATATCAATTTGGGCCCCA | DUXAP10 | DUXAP10-205 | 2.136 | 5.350 | 4.619 | 5.138 | 2.750 | 3.162 | 2.788 |
| ASHGA5P055877 | GCCAGAACATGGTGAATACAATCAAGTTCAGTGTATTTGATAATATCAATTTGGGCCCCA | DUXAP8 | DUXAP8-202 | 2.136 | 5.350 | 4.619 | 5.138 | 2.750 | 3.162 | 2.788 |
| ASHGA5P055877 | GCCAGAACATGGTGAATACAATCAAGTTCAGTGTATTTGATAATATCAATTTGGGCCCCA | DUXAP9 | DUXAP9-207 | 2.136 | 5.350 | 4.619 | 5.138 | 2.750 | 3.162 | 2.788 |
| ASHGA5P018683 | AGCAATGATCAGTATCAGCTCTTTATGGAGAAAGAGCAGCTCTGGAATGAGTCATTTTTT | AC138776.1 | AC138776.1-201 | 2.643 | 8.086 | 7.963 | 7.807 | 6.414 | 5.941 | 5.794 |
| ASHGA5P031697 | GAATCTTAACTGTATCCTTTTGAGAAAACAATGTACCCACATAAACTTCCTCCTTCTAAG | AC002519.1 | AC002519.1-201 | 1.839 | 6.841 | 7.120 | 7.361 | 4.942 | 5.450 | 5.411 |
| ASHGA5P018683 | AGCAATGATCAGTATCAGCTCTTTATGGAGAAAGAGCAGCTCTGGAATGAGTCATTTTTT | AC138776.1 | AC138776.1-201 | 2.643 | 8.409 | 8.207 | 8.150 | 6.768 | 6.343 | 6.296 |
| ASHGA5P026708 | CCAATGTATACCATTCTCAGTTGTCTATTTAAGGATTTCTTAGTGAGCTCCATGGTAAAA | AP003059.1 | AP003059.1-201 | 1.635 | 7.997 | 7.787 | 7.821 | 6.243 | 6.300 | 6.159 |
| ASHGA5P050748 | CCAGCCGCCTAGACGCTGGCACTATGGTCATGGCGGAGGGGACGGCAGTGCTGAGGCGGA | MOB4 | MOB4-208 | 1.498 | 8.252 | 8.436 | 8.465 | 6.625 | 7.189 | 6.846 |
| ASHGA5P013408 | CTAGATGAAGTGCTTGTCCTAGTACCTTAGCACATGGTTGCTGAATACATGAATGAAGAG | ANKRD36BP2 | ANKRD36BP2-201 | 1.360 | 10.793 | 10.629 | 10.713 | 9.663 | 9.103 | 9.288 |
| ASHGA5P040153 | TTGCCACTTATGTTCTTAGAAATACTTGCTTTAATTCACCAGAATCAGTTGCTCTGATAC | AC107223.1 | AC107223.1-216 | 1.302 | 7.154 | 6.975 | 6.960 | 6.015 | 5.594 | 5.575 |
| ASHGA5P042538 | GCTGAAGGATGGAGATACACTTTGGAGTAAGATTCATATGAACTTTCATAAATTCTTGGG | AL132996.1 | AL132996.1-202 | 1.289 | 4.139 | 4.046 | 4.131 | 2.986 | 2.758 | 2.705 |
| ASHGA5P034742 | TGAGTGAGTGAACTCTGTGCGAGATAAATTGGAGACAAATATGGACAACTCTCCCAAGGA | SPACA6 | SPACA6-208 | 1.174 | 7.871 | 7.825 | 7.727 | 6.782 | 6.650 | 6.468 |
| ASHGA5P031320 | AGCTGAAGAAGCTCTGGAAGATACCATTTGAAGTCTTGAGTGCTTGAACTCACAAAGAAA | MAFTRR | MAFTRR-201 | 1.055 | 4.420 | 4.624 | 4.726 | 3.458 | 3.504 | 3.644 |
| ASHGA5P046005 | TTTGTAAAATGTTGGCTGACGGCTAAGGGTGTTTATTCCTCTAAAATCACCTAATTAGAA | LINC02642 | LINC02642-201 | 1.052 | 3.999 | 4.034 | 4.002 | 2.978 | 3.108 | 2.794 |
| ASHGA5P020773 | CAAGGACACAGTGCTAGTATGAATGAATAATAATGGGGTTGTCTATGAAGAGGAGAGAAT | AF117829.1 | AF117829.1-201 | -1.041 | 4.724 | 4.732 | 4.859 | 5.852 | 5.957 | 5.628 |
| ASHGA5P031944 | CAACTCACATTAATGAATTCACTAAGTACCTAGCACTGAGAATAAAAAGCTAAAGCAGCA | AC092718.6 | AC092718.6-201 | -1.098 | 5.565 | 5.410 | 5.409 | 6.491 | 6.702 | 6.486 |
| ASHGA5P017232 | TTGTGCTGAGCAAGCCTCAGTGTATGGGCGCTGTTCATCCTCTGGTGCTGAAGCAGCCAA | AC018865.3 | AC018865.3-201 | -1.135 | 4.764 | 4.800 | 4.682 | 6.124 | 5.696 | 5.831 |
| ASHGA5P017232 | TTGTGCTGAGCAAGCCTCAGTGTATGGGCGCTGTTCATCCTCTGGTGCTGAAGCAGCCAA | AC140481.3 | AC140481.3-201 | -1.135 | 4.764 | 4.800 | 4.682 | 6.124 | 5.696 | 5.831 |
| ASHGA5P017232 | TTGTGCTGAGCAAGCCTCAGTGTATGGGCGCTGTTCATCCTCTGGTGCTGAAGCAGCCAA | CYP4F30P | CYP4F30P-201 | -1.135 | 4.764 | 4.800 | 4.682 | 6.124 | 5.696 | 5.831 |
| ASHGA5P017232 | TTGTGCTGAGCAAGCCTCAGTGTATGGGCGCTGTTCATCCTCTGGTGCTGAAGCAGCCAA | CYP4F62P | CYP4F62P-202 | -1.135 | 4.764 | 4.800 | 4.682 | 6.124 | 5.696 | 5.831 |
| ASHGA5P017232 | TTGTGCTGAGCAAGCCTCAGTGTATGGGCGCTGTTCATCCTCTGGTGCTGAAGCAGCCAA | FAR2P4 | FAR2P4-201 | -1.135 | 4.764 | 4.800 | 4.682 | 6.124 | 5.696 | 5.831 |
| ASHGA5P033754 | ATGTGAAAAAACTTTGGGACGATGAAGGCGTGAAGGCATGCTTTGAGAGATCCAACGAAT | GNAL | GNAL-209 | -1.153 | 5.040 | 4.834 | 5.080 | 5.947 | 6.339 | 6.129 |
| ASHGA5P036088 | ACCATTAGCACATAGCAGCAATTATTGACTAAATGGTGCTCTGGTTCCATGCCTTCCAAG | PAX8-AS1 | PAX8-AS1-225 | -1.204 | 5.089 | 5.066 | 4.988 | 6.286 | 6.219 | 6.250 |
| ASHGA5P047160 | CCTCTGTACCTGGACCAGGTAGAGACTTGATAAATATCTGTAGTTGTTAAATAAATGAAA | AL158166.2 | AL158166.2-201 | -1.215 | 3.000 | 2.981 | 2.800 | 4.044 | 4.075 | 4.307 |
| ASHGA5P020282 | GGGGAGACCCACACATATACTCACAGAAATGAGTTATTTAAAGAAGACGGTCCAGATTCC | PAX8-AS1 | PAX8-AS1-201 | -1.231 | 5.680 | 5.653 | 5.489 | 6.877 | 6.766 | 6.874 |
| ASHGA5P014521 | AAGGGAGAGTGGATTTGGCTGGGCCATCTGGATGGAAGGTCTGGTCTTCTCTCGTCTGAG | GNLY | GNLY-213 | -1.234 | 11.098 | 11.120 | 11.106 | 12.326 | 12.340 | 12.361 |
| ASHGA5P030665 | GTGTTAGATGGACGGAATTTGACAACTGTACTGCAGTCGTCAGAGAGTATCTTCTTCTTG | AC090181.1 | AC090181.1-201 | -1.255 | 5.719 | 5.280 | 5.272 | 6.714 | 6.666 | 6.654 |
| ASHGA5P019838 | CACCACAAATAGTTACAGACTAAATCATCCTTGATTCCTTCTTCTTCAAGTATTAGAGTC | AC095056.1 | AC095056.1-201 | -1.304 | 3.058 | 3.098 | 2.800 | 4.280 | 4.407 | 4.181 |
| ASHGA5P014521 | AAGGGAGAGTGGATTTGGCTGGGCCATCTGGATGGAAGGTCTGGTCTTCTCTCGTCTGAG | GNLY | GNLY-213 | -1.234 | 12.000 | 11.912 | 11.822 | 13.329 | 13.160 | 13.282 |
| ASHGA5P034869 | TCTCCATACATATCATGAGATATTCTTCACCAGAATAAGCTTTTGTTTCACTACCAAGAG | LINC00299 | LINC00299-203 | -1.368 | 5.411 | 5.602 | 5.643 | 6.951 | 6.911 | 6.898 |
| ASHGA5P018480 | GAAAAATCAGATTTTGTGAACAATTTGGCATGAGGAGAGAAAGGAACCTGAAGTTTTTAG | LINC00299 | LINC00299-201 | -1.370 | 6.715 | 6.711 | 6.554 | 8.114 | 7.844 | 8.132 |
| ASHGA5P058217 | TTTCAGGAACTGCTGAAGAAAATCACCAGAAAATCATACTGGTAGCCTTCAAGTGGCCAT | MIR646HG | MIR646HG-207 | -1.373 | 6.972 | 6.700 | 6.655 | 7.878 | 8.260 | 8.307 |
| ASHGA5P017716 | GCAGAGCCAGTGGAATGTCACCATCGCCCAGGTGGGGATTTTTGTGTGTTTTGTTCACTG | SMIM25 | SMIM25-204 | -1.412 | 8.097 | 8.055 | 8.147 | 9.192 | 9.811 | 9.533 |
| ASHGA5P055414 | ATCACAACTTTAAACCCCGTCCGTACTGAAGCCTGCGTCCGAATTTCTTCTGTTTTTGTG | AL590226.2 | AL590226.2-201 | -1.426 | 10.312 | 10.421 | 10.412 | 11.989 | 11.636 | 11.798 |
| ASHGA5P017067 | TCACTATCCTAGAGTAACTAATGGAAACAGCTTCAGGAAACAGACTTAAAGATGGGTTTC | AC010105.1 | AC010105.1-201 | -1.427 | 3.084 | 2.768 | 2.800 | 4.446 | 4.044 | 4.443 |
| ASHGA5P018480 | GAAAAATCAGATTTTGTGAACAATTTGGCATGAGGAGAGAAAGGAACCTGAAGTTTTTAG | LINC00299 | LINC00299-201 | -1.370 | 7.355 | 7.224 | 6.967 | 8.613 | 8.645 | 8.589 |
| ASHGA5P020282 | GGGGAGACCCACACATATACTCACAGAAATGAGTTATTTAAAGAAGACGGTCCAGATTCC | PAX8-AS1 | PAX8-AS1-201 | -1.231 | 5.891 | 6.024 | 5.623 | 7.496 | 7.055 | 7.300 |
| ASHGA5P018480 | GAAAAATCAGATTTTGTGAACAATTTGGCATGAGGAGAGAAAGGAACCTGAAGTTTTTAG | LINC00299 | LINC00299-201 | -1.370 | 7.114 | 7.023 | 7.274 | 8.642 | 8.698 | 8.571 |
| ASHGA5P036024 | TATTCTCACTATGAACATTTCAGAACTCTGAAGATCTGGACAGCTTTCTAACACCTGTTC | AC007278.2 | AC007278.2-201 | -1.504 | 6.409 | 6.210 | 5.798 | 7.615 | 7.720 | 7.595 |
| ASHGA5P055414 | ATCACAACTTTAAACCCCGTCCGTACTGAAGCCTGCGTCCGAATTTCTTCTGTTTTTGTG | AL590226.2 | AL590226.2-201 | -1.426 | 9.351 | 9.338 | 9.174 | 11.036 | 10.494 | 10.870 |
| ASHGA5P014553 | AGGCCGTGAGTCAGCCATAATGGCAGGTGAAGAAATTAATGAAGACTATCCAGTAGAAAT | C1D | C1D-205 | -1.559 | 7.758 | 7.894 | 8.021 | 9.312 | 9.561 | 9.478 |
| ASHGA5P034153 | CACTTATTATCCTTTCATTGTCTGCCAGATCTGTGGCATTGCTTTTTTCTCTGGTATTAG | AC008555.1 | AC008555.1-201 | -1.573 | 7.013 | 6.726 | 6.354 | 8.100 | 8.433 | 8.279 |
| ASHGA5P045272 | GCACCACATTGATTTACTGCACCAGGCTTTCTTCATTGTGATGATGTTCTCTCTCTTTTC | BANCR | BANCR-201 | -1.711 | 2.793 | 2.845 | 2.800 | 4.174 | 4.488 | 4.907 |
| ASHGA5P045235 | AACTGTCTACCTGGGAGATGTTGCTGTTAGTCTAACCTGTACCATTTTGTAAACCTGCAG | FAM95B1 | FAM95B1-212 | -1.837 | 7.589 | 7.336 | 6.868 | 9.123 | 9.170 | 9.011 |
| ASHGA5P040819 | TGTTTTGGACTGAGTTTAGTTCTCATTAGTAGGATGCAGGGGGTTTTTGATGGTGTCTTT | SAP30L-AS1 | SAP30L-AS1-204 | -1.909 | 2.932 | 2.761 | 2.800 | 4.330 | 5.192 | 4.697 |
| ASHGA5P029787 | GAGTGTTACTGGGTTTTGTGAATCACGGGCCTTGAAAGTATTATTAATGAATAAATGACA | MEG8 | MEG8-205 | -1.926 | 3.474 | 3.774 | 3.853 | 5.277 | 6.014 | 5.588 |
| ASHGA5P056389 | AGATGGACAGATTAGCCTGGATTAAGCAGATGGAGAAGCCGAGGATCAAGGATCGGGCCA | AC011246.1 | AC011246.1-201 | -2.184 | 3.731 | 3.094 | 2.800 | 5.164 | 5.596 | 5.418 |
| ASHGA5P054841 | AGCAGATGTGGAGCTACTCTAGTACGAACTGACCATGGGGTATCATGATGATGAAGATTC | TRIM51HP | TRIM51HP-201 | -2.292 | 3.230 | 2.776 | 2.800 | 4.990 | 5.249 | 5.443 |
| ASHGA5P050874 | TTCCTTTTGTATTAAAAATGCAGTGTTGGGGAGGTAACGCTGTATATTTCTGTTTGCAAG | AC104809.2 | AC104809.2-201 | -3.114 | 9.670 | 9.314 | 8.587 | 11.879 | 12.614 | 12.419 |
| ASHGA5P044870 | GTGAAGAATGAGGAGCTGTAGATTTTTAACGATTCTGATGTATTTGCATTTTTCCCAATG | AC022730.4 | AC022730.4-201 | -6.169 | 2.880 | 2.773 | 2.800 | 8.192 | 9.677 | 9.091 |

Note:

**Probe ID**: probe name in Agilent-045997 Arraystar human lncRNA microarray V3 Platform.

**Sequence**: *in situ* oligonucleotide sequence for each Probe ID.

**Gene symbol**: lncRNA gene name corresponding to probe sequence by BLASTing probe sequence to Fasta files from GENCODE Human database.

**LncRNA transcript**: one representative transcript for each lncRNA gene.

**LOG2FC**: log2 transformed value for fold change between ATB and Control group.

**ATB 1#～3# & Control 1#～3#:** Normalized signal intensity for each sample.
